# Identification and clinical validation of EMT-associated prognostic features based on hepatocellular carcinoma

**DOI:** 10.1186/s12935-021-02326-8

**Published:** 2021-11-24

**Authors:** Dafeng Xu, Yu Wang, Jincai Wu, Shixun Lin, Yonghai Chen, Jinfang Zheng

**Affiliations:** 1 Department of Hepatobiliary and Pancreatic Surgery, Hainan General Hospital, Hainan Affiliated Hospital of Hainan Medical University, Haikou, Hainan, China; 2 Geriatric Medicine Center, Hainan General Hospital, Hainan Affiliated Hospital of Hainan Medical University, Haikou, Hainan, China

**Keywords:** EMT, HCC, Prognosis, G6PD

## Abstract

**Background:**

The aim of this study was to construct a model based on the prognostic features associated with epithelial–mesenchymal transition (EMT) to explore the various mechanisms and therapeutic strategies available for the treatment of metastasis and invasion by hepatocellular carcinoma (HCC) cells.

**Methods:**

EMT-associated genes were identified, and their molecular subtypes were determined by consistent clustering analysis. The differentially expressed genes (DEGs) among the molecular subtypes were ascertained using the limma package and they were subjected to functional enrichment analysis. The immune cell scores of the molecular subtypes were evaluated using ESTIMATE, MCPcounter, and GSCA packages of R. A multi-gene prognostic model was constructed using lasso regression, and the immunotherapeutic effects of the model were analyzed using the Imvigor210 cohort. In addition, immunohistochemical analysis was performed on a cohort of HCC tissue to validate gene expression.

**Results:**

Based on the 59 EMT-associated genes identified, the 365—liver hepatocellular carcinoma (LIHC) samples were divided into two subtypes, C1 and C2. The C1 subtype mostly showed poor prognosis, had higher immune scores compared to the C2 subtype, and showed greater correlation with pathways of tumor progression. A four-gene signature construct was fabricated based on the 1130 DEGs among the subtypes. The construct was highly robust and showed stable predictive efficacy when validated using datasets from different platforms (HCCDB18 and GSE14520). Additionally, compared to currently existing models, our model demonstrated better performance. The results of the immunotherapy cohort showed that patients in the low-risk group have a better immune response, leading to a better patient’s prognosis. Immunohistochemical analysis revealed that the expression levels of the FTCD, PON1, and TMEM45A were significantly over-expressed in 41 normal samples compared to HCC samples, while that of the G6PD was significantly over-expressed in cancerous tissues.

**Conclusions:**

The four-gene signature construct fabricated based on the EMT-associated genes provides valuable information to further study the pathogenesis and clinical management of HCC.

**Supplementary Information:**

The online version contains supplementary material available at 10.1186/s12935-021-02326-8.

## Background

Liver cancer is the sixth most common type of cancer in the world and the third leading cause of cancer-related deaths worldwide, with high morbidity and mortality as well as an extremely poor prognosis [1]. Hepatocellular carcinoma (HCC) is the most common type of liver cancer, accounting for 85–90% of all primary liver cancers and causing 700,000 deaths worldwide each year; it is more prevalent and fatal in developing countries [2–4]. Until recently, Sorafenib, a kinase inhibitor drug, was the only systemic treatment option available for patients with advanced HCC. In 2020, atezolizumab and bevacizumab combination therapy turned into a new frontline standard of care for unresectable or metastatic HCC [5]. Despite advancement in treatment strategies in recent decades, the overall 5-year survival rate of patients with HCC is currently less than 12%. This is primarily due to the high recurrence rate and the intra- or extra-hepatic metastases. Most patients with HCC are diagnosed at the advanced stage and therefore, experience limited clinical benefit from treatment [6–9]. Since HCC has a rather poor prognosis and is highly resistant to most anticancer therapies, efforts have been made to unravel the complex molecular mechanisms underlying hepatocarcinogenesis and progression, including epithelial mesenchymal transition (EMT), tumor-stromal interactions, tumor microenvironment, tumor stem cells, and evasion of senescence [10]. A better understanding of these mechanisms can enable the development of new and more effective therapeutic and prognostic strategies, which is the need of the hour.

EMT is an important biological process in embryonic development, cell differentiation and reprogramming, and cancer progression [11, 12]. A growing body of evidence suggests that EMT confers tumor stem cell-like features, which results in treatment resistance and tumor recurrence [13]. Therefore, EMT is considered as one of the primary mechanisms determining cancer cell invasion and metastasis [14]. Much evidence suggests that EMT is associated with the invasion and progression of various malignancies, including HCC [15, 16]. EMT in HCC cells, similar to other tumors, appears to be driven by the aberrant activation of the Wnt/β-catenin signaling pathway [17–20], which increases hypoxia-induced EMT in HCC [21]. Mounting evidence suggests that EMT aids in cell proliferation, invasion, and metastasis during HCC progression, and contributes to chemotherapy resistance, thereby leading to poor patient prognosis [22–24]. In addition, EMT has also been found to positively correlate with resistance to sorafenib, cis-platin, and Adriamycin [25–27]. However, a pervious study has revealed that sorafenib inhibits the migration of HCC cells by inhibiting EMT, which is one of the potential mechanisms responsible for the antitumor effect of sorafenib in HCC [28]. Although the mechanisms underlying EMT in HCC have been extensively studied, the prognostic value and the biological role of EMT-associated genes have not been elucidated. Therefore, studying the molecular subtypes of HCC with respect to EMT, and evaluating their prognostic relevance, is of great importance to identify therapeutic targets and improve the prognosis of HCC patients.

In this study, we identified certain EMT-associated genes and constructed molecular subtypes of liver hepatocellular carcinoma (LIHC) models based on EMT. Subsequently, we evaluated the relationship between the molecular subtypes, and their prognostic and clinical features. A four-gene signature (*PON1*, *FTCD*, *G6PD*, and *TMEM45A*) prognostic risk model was constructed using the DEGs identified among the LIHC molecular subtypes and validated using the HCCDB and GEO gene expression datasets. On validation, we found that the constructed four-gene prognostic marker showed good performance, therefore proving useful for the prognostic classification of HCC patients and identification of new therapeutic targets for HCC.

## Materials and methods

### Data source and pre-processing

The expression data and clinical follow-up information coming from LIHC patient tissues were downloaded using TCGA, and the data were processed through the following steps: (1) Samples without clinical follow-up information were removed. (2) Ensemble was converted to Gene Symbol. (3) The middle value was taken in the presence of multiple Gene Symbol expressions.

The GSE14520 microarray dataset was downloaded from Gene Expression Omnibus (GEO), and the GEO dataset was processed with the following steps: (1) Samples without clinical follow-up information were removed. (2) Probes were converted to Gene Symbols. (3) Removal of probe corresponding to multiple genes. (4) The middle value was taken in the presence of multiple Gene Symbol expressions.

The HCCDB18 data were downloaded from the HCCDB18 database (http://lifeome.net/database/hccdb/home.html), and the RNA-Seq data were processed in the following steps: (1) Samples without clinical follow-up information were removed. (2) Samples without expression profile data were removed.

The genes of EMT-associated pathways (HALLMARK_EPITHELIAL_ MESENCHYMAL_TRANSITION) were downloaded from Molecular Signature Database v7.0 (MSigDB), and a total of 200 EMT-associated genes were collated and gathered.

After preprocessing three datasets, a total of 365 samples for TCGA-LIHC, 203 samples for HCCDB18, and 221 samples for GSE14520 were attained. The clinical statistical information of the samples is shown in Table 1.

**Table 1:** Cohorts information

Clinical features	TCGA-LIHC	HCCDB18	GSE14520
OS			
0	235	168	136
1	130	35	85
T stage			
T1	180		
T2	91		
T3	78		
T4	13		
TX	3		
N stage			
N0	248		
N1	4		
NX	113		
M stage			
M0	263		
M1	3		
MX	99		
Stage			
I	170		
II	84		
III	83		
IV	4		
X	24		
Grade			
G1	55		
G2	175		
G3	118		
G4	12		
GX	5		
Gender			
Male	246		
Female	119		
Age			
≤ 60	173		
> 60	192		
Recurrence			
Yes	198		
No	167		

### Identification of molecular subtypes using the ConsensusClusterPlus algorithm

The TCGA expression profile data were first filtered by the process of removing genes having expressions less than 1, which accounted for less than 50% of all samples, and a univariate COX analysis was performed to filter out unnecessary genes at a threshold of P < 0.05. EMT genes associated with prognosis were obtained, followed by the application of consistent clustering of TCGA samples using ConsensusClusterPlus (V1.48.0; parameters: reps = 100, pItem = 0.8, pFeature = 1, distance = “spearman”).

D2 and Euclidean distance were used as clustering algorithms and distance metrics respectively to obtain molecular subtypes. DEGs between molecular subtypes were calculated using the limma package and subjected to functional enrichment analysis. GSEA was used in the LIHC dataset to analyze the significantly enriched pathways in different groups, where the selected gene set was c2.cp.kegg.v7.0.symbols.gmt that contains the KEGG pathway. The GSEA input file containing the TCGA expression profile data and the molecular subtype were labeled as C1 or C2 group according to the sample labels. The enriched pathways were selected having a threshold of p < 0.05 and FDR < 0.25 as the basis.

### Construction of prognostic risk model based on EMT genes

#### Partitioning of the training and validation sets

A total of 365 samples in the TCGA dataset were subdivided into training and validation sets. In advance, all samples were randomly grouped 100 times with replacement and group sampling was performed in the ratio of training set: validation set = 1:1 to avoid random assignment bias affecting the stability of subsequent modeling. The most suitable training and validation sets were selected based on the following set conditions: (1) the two groups had to have similar age distribution, gender, follow-up time, and the proportion of patient deaths; (2) there was a similar number of dichotomous samples between the randomly grouped cohorts following gene expression profile clustering. The selection yielded 182 samples in the training set and 183 in the validation set.

The information on both the training and validation set samples of the TCGA data, shown in Table 2, were tested using the chi-square test and showed a validated grouping method having no significant difference between groups (p > 0.05).

**Table 2:** Sample information of TCGA training set and validation set

Clinical features	TCGA-LIHC train	TCGA-LIHC test	P
OS			
0	116	119	0.8822
1	66	64	
T stage			
T1	94	86	0.3129
T2	37	54	
T3	41	37	
T4	8	5	
TX	2	1	
N stage			
N0	123	125	0.5998
N1	3	1	
NX	56	57	
M stage			
M0	132	131	0.1966
M1	3	0	
MX	47	52	
Stage			
I	90	80	0.3149
II	34	50	
III	43	40	
IV	3	1	
X	12	12	
Grade			
G1	29	26	0.9548
G2	88	87	
G3	56	62	
G4	6	6	
GX	3	2	
Gender			
Male	119	127	0.48
Female	63	56	
Age			
≤ 60	87	86	0.9604
> 60	95	97	

### Lasso cox regression analysis

The lasso regression was employed on prognostic genes to reduce the number of genes found in the risk model. Lasso method is a type of shrinkage estimation that obtains a more refined model by constructing a penalty function that allows shrinking of coefficients, setting some coefficients to zero, and retaining the advantage of subset shrinkage. This is a biased estimate that deals with data multicollinearity that achieves variable selection, parameter estimation, and problem-solving capabilities for multicollinearity in regression analysis. The trajectory of each independent variable was analyzed by performing lasso cox regression using the R package glmnet. Subsequently, an optimal model was constructed using fivefold cross-validation to analyze the confidence intervals under each lambda and to find the number of targeted genes.

### Immunohistochemistry

To verify the expression of the candidate four genes, tissue microarrays (TMA) comprised of 41 HCC tissues and 41 normal samples were obtained from Shanghai Outdo Biotech Co., Ltd. (Shanghai, China). The studies were conducted in accordance with the International Ethical Guidelines for Biomedical Research Involving Human Subjects (CIOMS), and the research protocols were approved by the Ethics Committee of Hainan General Hospital, Hainan Affiliated Hospital of Hainan Medical University.

The TMA slides were dried overnight at 37 °C, dewaxed in xylene, and dehydrated in a gradient ethanol series. Antigen’s retrieval was performed by heating the tissue sections in a microwave oven inside a vessel filled with EDTA antigen retrieval buffer (pH 9.0). Subsequently, the tissue sections were immersed in 3% hydrogen peroxide for 25 min to block the activity of endogenous peroxides. Next, the TMA tissues were coated with 3% bovine serum albumin (BSA) and sealed at room temperature for 30 min to reduce non-specific staining. Then, the TMA slides were incubated with anti-TMEM45A (1:500 dilution; Sigma, HPA062101), anti-PON1 (1:100 dilution; Abcam, ab92466), anti-FTCD (1:250 dilution; Abcam, ab129005)and anti-G6PD (1:2000 dilution; Abcam, ab210702) overnight at 4 °C.

The tissues were rinsed with 0.01 mol/L phosphate buffer saline (PBS; pH = 7.4) for 5 min each. The tissues were incubated at room temperature for 50 min with horseradish peroxidase (HRP)-labeled goat anti-rabbit secondary antibody (1:200 dilution, ServiceBio, GB23303). Then, the tissues were washed in PBS and stained with 3,3-diaminobenzidine (DAB). Finally, the TMA sections were counterstained with Mayer’s hematoxylin, dehydrated, and fixed. To evaluate IHC staining, semi-quantitative scoring criteria were used.

The stained sections were scored by three pathologists who were blinded to the patients’ clinical characteristics. The scoring system was based on the proportion of positively stained cells in all tissues and the staining intensity of these positively stained cells. The staining intensity was classified as follows: 0 (negative), 1 (weak), 2 (moderate), or 3 (strong). The staining ratio of positive cells was classified as follows: 0 (< 5%), 1 (5–25%), 2 (26–50%), 3 (51–75%), or 4 (> 75%). According to the staining intensity and the proportion of positively stained cells, the tissues were graded as follows: 0–1 grade, negative (−); > 1–4, weakly positive (+); > 4–8, moderately positive (++), and > 8–12, strongly positive (+++).

## Results

 Identification of molecular subtypes using non-negative matrix factorization (NMF) algorithm

**Fig. 1:**
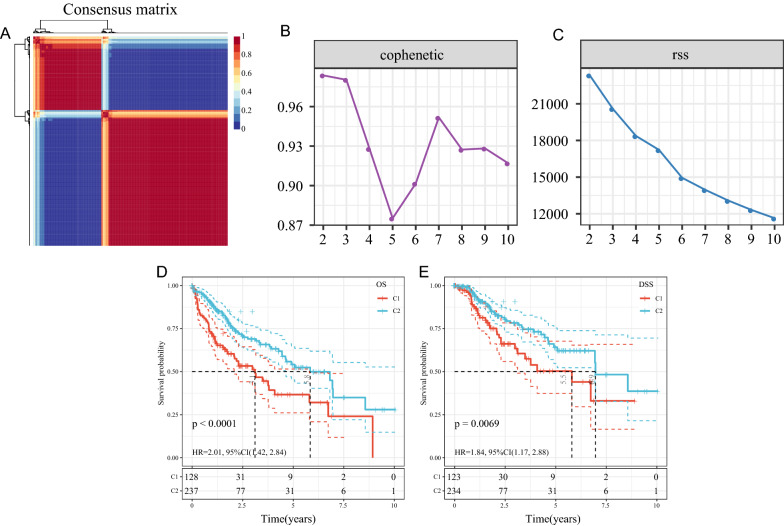
**A** Consensus map of NMF clustering; **B** cophenetic distribution having rank = 2–10, where cophenetic correlation is obtained from the consistency matrix proposed by Brunet et al. that is reflective of the stability of the cluster obtained from NMF. The value is between 0 and 1, where a larger value signified a more stable cluster; **C** the rss distribution with rank = 2–10. Rss is the residual sum of squares and shows clustering performance of a model, where a smaller value yielded a better clustering effect of the model in contrast to the theoretical concept that the smallest value is attained when each sample is clustered into one class; **D** overall survival (OS) time prognosis survival curve of LIHC molecular subtypes; **E** DSS time prognosis survival curve of LIHC molecular subtypes

The expression of 200 EMT genes was first extracted from the TCGA expression profile data, followed by univariate cox analysis by coxph function in R. Fifty-nine genes (Additional file 1: Table S1) associated with LIHC prognosis (p < 0.05) were obtained, and the LIHC samples were clustered by non-negative matrix factorization algorithm (NMF). An optimal clustering of k = 2 was determined based on cophenetic, a residual sum of squares, and other metrics, thus obtaining two molecular subtypes (C1, C2) (Fig. 1A–C).

Further analysis on the prognostic relationship between subtypes revealed a significant difference between C1 and C2 groups in terms of overall survival (OS) time and disease-specific survival (DSS) time (Fig. 1D, E, log-rank p < 0.01). It was also found that the C1 subtype carried a poorer prognosis.

### Comparison and analysis of immune and matrix scores between molecular subtypes

**Fig. 2:**
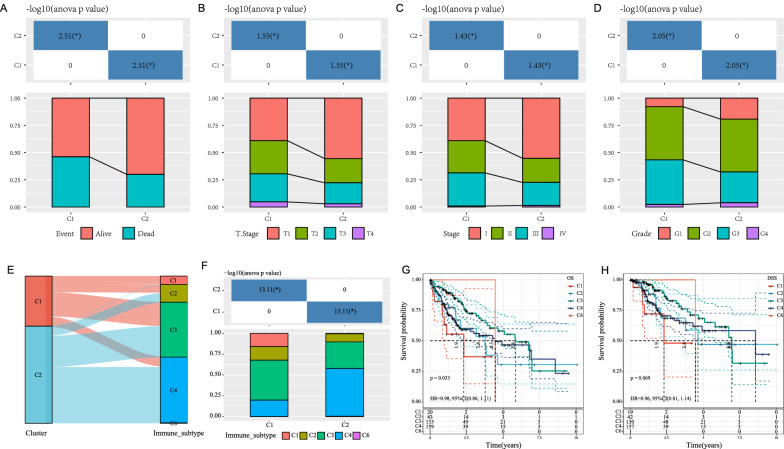
**A**–**D** A comparison of distribution as seen in different clinical features of the two molecular subtypes in the TCGA dataset; **E** comparison between metabolic subtypes and existing molecular subtypes; **F** distribution comparison between different metabolic subtypes; **G** KM curve of OS time between existing immune molecular subtypes; **H** KM curve of DSS time between existing immune molecular subtypes

**Fig. 3:**
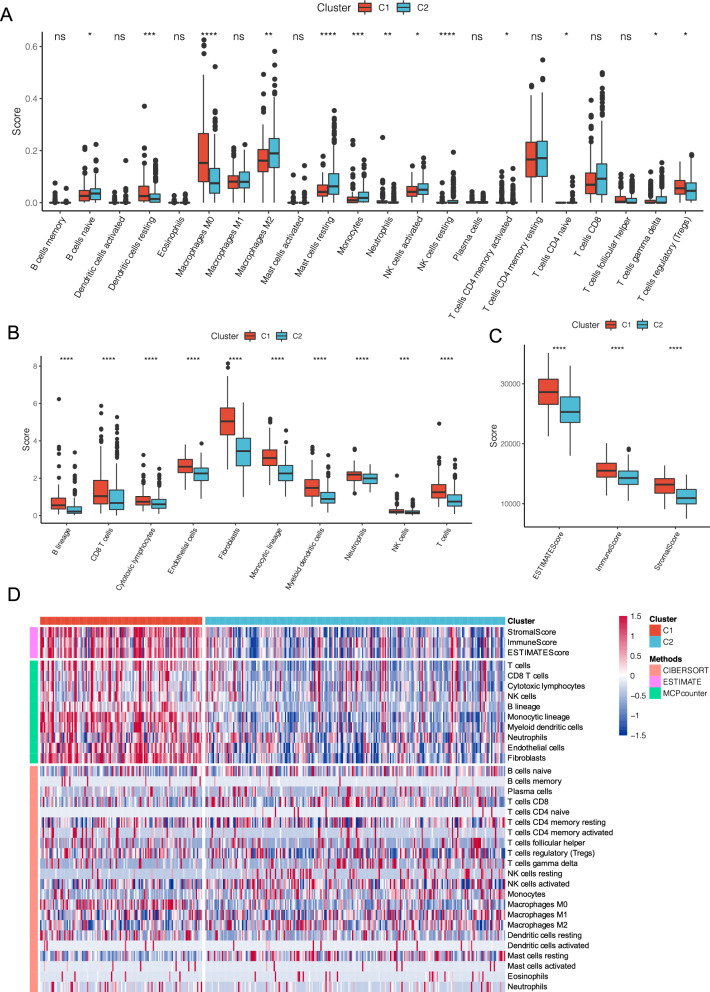
Comparison of immune scores as seen in the three-immune software between molecular subtypes of TCGA dataset. **A** Comparison of CIBERSOTR immune scores between molecular subtypes of TCGA dataset; **B** comparison of MCPcounter immune scores between molecular subtypes of TCGA dataset; **C** comparison of ESTIMATE immune scores between molecular subtypes of TCGA dataset; **D** comparison of three immune software immune scores between molecular subtypes of TCGA dataset

**Fig. 4:**
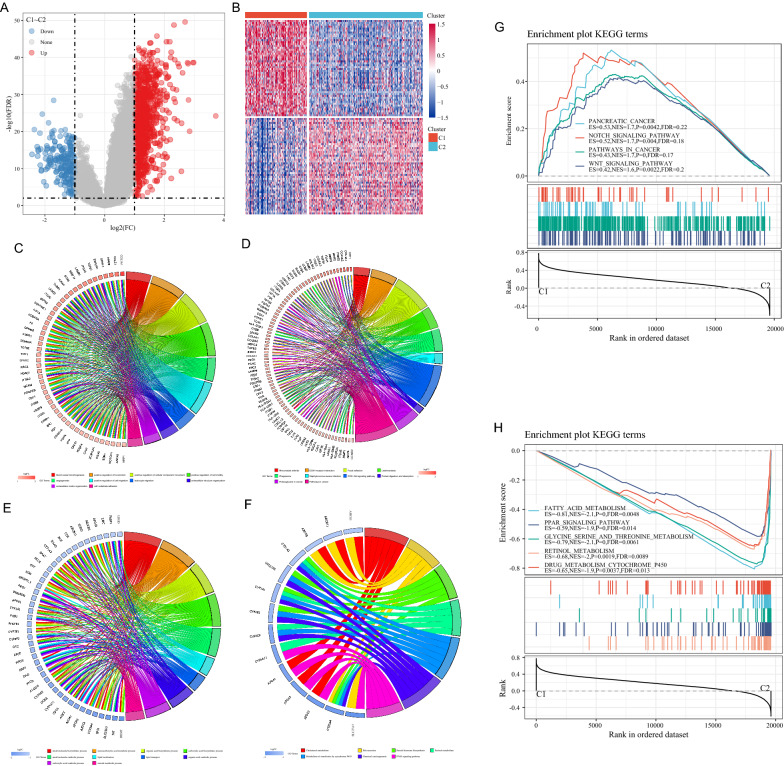
**A** Volcano map of differential genes in C1 and C2 groups; **B** heat map of differential genes in C1 and C2 groups. **C** Biological process (BP) annotation map of differentially up-regulated genes in molecular subtypes; **D** KEGG annotation map of differentially up-regulated genes in molecular subtypes. **E** BP annotation map of differentially down-regulated genes in molecular subtypes; **F** KEGG annotation of differentially down-regulated genes in molecular subtypes; **G**, **H** correlation pathways between molecular subtypes

The different clinical features in two molecular subtypes were compared, the results revealed that (1) survival rates were significantly different amongst the two subtypes and the C1 group showed a poorer prognosis (Fig. 2A); (2) the proportion of T-stage was significantly different between the two subtypes, and a higher proportion of T2, T3, and T4 in the C1 group carried a poor prognosis (Fig. 2B); (3) the proportion of Stage was significantly different between the two subtypes, with a higher proportion of Stage II and III in the C1 group with poor prognosis (Fig. 2C); (4) the proportion of Grade was significantly different between the two subtypes, with a higher proportion of G3 in the C1 group having poor prognosis (Fig. 2D).

The six types of immune infiltration identified in human tumors are as follows: C1 (wound healing), C2 (INF-r dominant), C3 (inflammation), C4 (lymphocyte depletion), C5 (immunologically silent,) and C6 (TGF-beta dominant) [29]. Utilizing this subtyping outcome, KM curves were plotted and found that the immune subtype C1 had the worst prognosis (Fig. 2G, H). A comparison was made between this subtyping method and the study’s subtyping sample (Fig. 2E, F) and an analysis of the distribution of this subtyping against the study’s EMT molecular subtypes indicated a consistent tendency that immune subtype C1 was more distributed in the molecular subtype C1.

### Comparison of immune scores between molecular subtypes

To identify the relationship of immune scores between molecular subtypes in the TCGA dataset, the R software package ESTIMATE was used to evaluate three immune scores of StromalScore, ImmuneScore, and ESTIMATEScore. Ten and twenty-two immune cells scores were also evaluated using MCPcounter and CIBERSOTR, respectively.

Comparing the differences of immune scores in molecular subtypes (Fig. 3A–C) elucidated that the immune scores of C1 subtypes were generally higher than C2 subtypes as seen in the three software. Heat maps of the immune score also visually demonstrated the differences in immune scores between subtypes (Fig. 3D).

### Identification of DEGs between subtypes and functional analysis of pathways

DEGs between C1 and C2 molecular subtypes were calculated using the limma package, with a total of 1130 DEGs filtered according to the threshold FDR < 0.01 and |log2FC| > 1, of which 931 were up-regulated genes and 199 were down-regulated genes. An up-regulated expression pattern between C1 and C2 was the predominant form (Fig. 4A). The DEGs are shown in Additional file 2: Table S2 and the 100 genes with the largest up- and down-regulation were selected and plotted in the heat map (Fig. 4B).

The KEGG pathway analysis and GO functional enrichment analysis were performed on 931 up-regulated differential genes of LIHC subtypes using the Goplot R package, in which 1001 annotated genes showed significant differences in Biological Process (BP) (Fig. 4C, FDR < 0.05).

As to the differential genes of LIHC, KEGG pathway enrichment was performed and genes were significantly enriched in: ECM-receptor interaction, Proteoglycans in cancer, Rap1 signaling pathway, PI3K-Akt signaling pathway, Pathways in cancer, and other tumor-related pathways (Fig. 4D). The same approach was used to perform both pathway analysis and functional enrichment analysis for 199 down-regulated differential genes. The down-regulated genes were also found to be significantly enriched in: retinol metabolism, glycine, serine, and threonine metabolism, metabolism of xenobiotics by cytochrome P450, PPAR signaling pathway, and other metabolism-related pathways (Fig. 4E, F).

Further employment of GSEA revealed that PATHWAYS_IN_CANCER, WNT_SIGNALING_PATHWAY, and NOTCH_SIGNALING_PATHWAY and other tumor-related pathways were enriched in the C1 subtype group (Fig. 4G), thus showing a clear relationship between C1 subtype and tumors; in contrast, metabolism-related pathways such as: FATTY_ACID_METABOLISM, PPAR_SIGNALING_PATHWAY, and DRUG_METABOLISM_CYTOCHROME_P450 were more enriched in the C2 subtype group (Fig. 4H) depicting a close association between C2 subtype and metabolism.

### Construction of a prognostic risk model

**Fig. 5:**
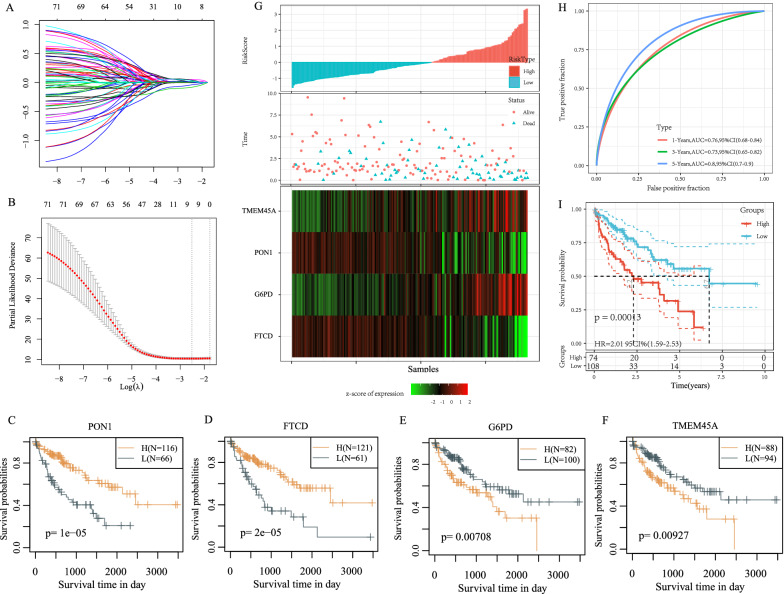
**A** Independent variable trajectory: horizontal axis (representing the log value of the dependent lambda) and the vertical axis (representing the coefficient of the independent variable); **B** confidence interval under each lambda; **C**–**F** KM curves of 4 genes (TCGA training set)

A total of 182 samples were obtained from the training set while 183 samples were derived from the validation set (Table 2). The data in the training set (a total of 1130 differential genes of C1 and C2 molecular subtypes) was subjected to the univariate Cox proportional risk regression model and the survival data used the R package surv coxph function, where p < 0.01 was selected as the threshold for filtering and resulted to a total of 71 prognostic genes (Additional file 3: Table S3). However, these genes are not conducive to clinical testing and still need further reduction using the lasso regression to maintain a high accuracy rate. The R package glmnet was used for lasso cox regression analysis, where the independent variable trajectories were analyzed (Fig. 5A), and the results showed that as lambda gradually increases, the number of independent variable coefficients also gradually increases. Fivefold cross-validation was done to analyze the confidence intervals under each lambda (Fig. 5B) and indicated that the model was optimal when lambda = 0.08091131, therefore 9 genes at lambda = 0. 08091131 were selected as the target genes for the next step.

The Akaike Information Criterion (AIC), a stepwise regression, was utilized to take into account the statistical fit of both models and parameters. The AIC method in the MASS package first started off with a complex model that later removed one variable to reduce the AIC thus resulting in a better model having fewer parameters. Using this algorithm, the final 9 genes were reduced to 4 genes, namely: PON1, FTCD, G6PD, and TMEM45A, and the prognostic KM curves for the 4 genes (Fig. 5C–F) can significantly separate the TCGA training set samples (p < 0.05). The final 4-gene signature formula is as follows: RiskScore = − 0.125 * PON1 − 0.144FTCD + 0.133 * G6PD + 0.134 * TMEM45A.

The calculation of RiskScore for each sample was according to the expression levels per sample and the higher the RiskScore correlated with a worse prognosis (Fig. 5G). The R package time ROC performed an analysis for the prognostic classification of RiskScore at 1 year, 3 years, and 5 years, which showed the model with a high AUC above 0.7 (Fig. 5H). Finally, samples with a Riskscore greater than zero and less than zero were classified as high-risk group and low-risk group respectively. A plotted KM curve classified 74 samples as a high-risk group and 108 samples as a low-risk group (Fig. 5I).

### Validation of the risk model

**Fig. 6:**
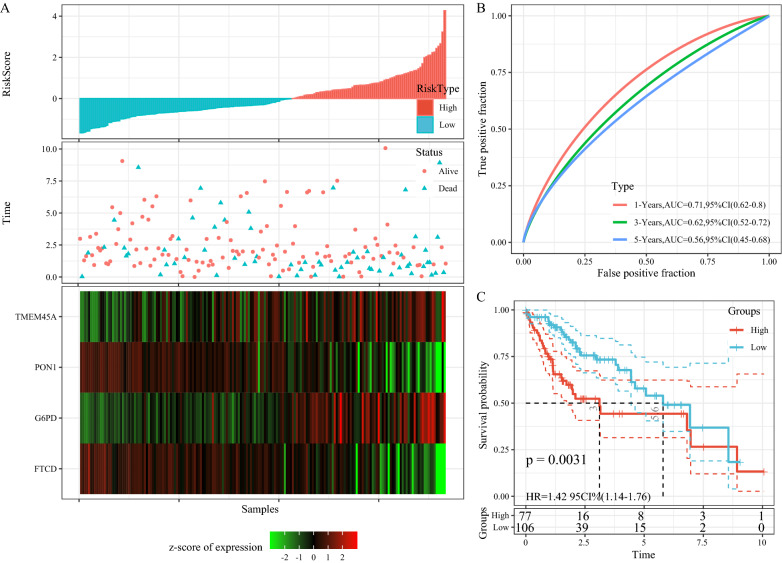
**A** RiskScore, TTL, survival status, and 4-gene expression in TCGA test set; **B** ROC curve and AUC of 4-gene signature classification; **C** KM survival curve distribution of 4-gene signature in TCGA test set

To determine the robustness of the model, a validation set of the TCGA applied the same model and the same coefficients as the training set. The RiskScore distribution of the TCGA validation set (Fig. 6A), showed that LIHC samples with high RiskScores correlating with worse prognosis were significantly smaller than those with low RiskScores. In addition, prognostic prediction efficiency at 1 year, 3 years, and 5 years was analyzed using R package timeROC (Fig. 6B). The plotting of KM curves showed that 77 samples were classified as a high-risk group while 106 samples were categorized as a low-risk group (Fig. 6C, p < 0.01).

**Fig. 7:**
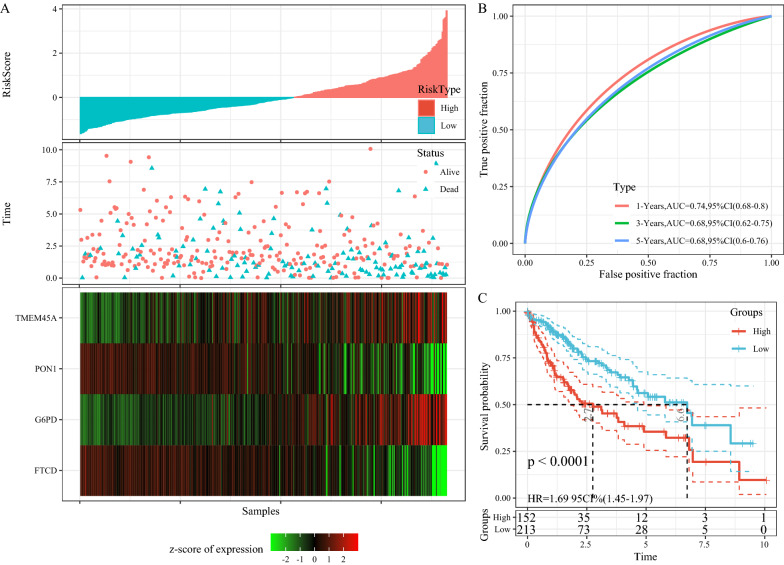
**A** RiskScore, TTL, survival status and 4-gene expression in TCGA full dataset; **B** ROC curve and AUC of 4-gene signature classification; **C** KM survival curve distribution of 4-gene signature in TCGA full dataset

The distribution of RiskScore for the full TCGA dataset (Fig. 7A) suggested that high RiskScore samples have a worse prognosis and the prognostic prediction efficiency of RiskScore at 1 year, 3 years, and 5 years was analyzed using the R software package timeROC (Fig. 7B). The plotted KM curves showed that 152 samples were classified as a high-risk group and 213 samples as a low-risk group (Fig. 7C, p < 0.001).

### Robustness of 4-gene signature verified by external dataset

The same model and coefficients as in the training set were used in the external validation sets GSE14520 and HCCDB18. The RiskScore of each sample was calculated separately based on the expression level of each sample followed by the plotting of the RiskScore distribution.

**Fig. 8:**
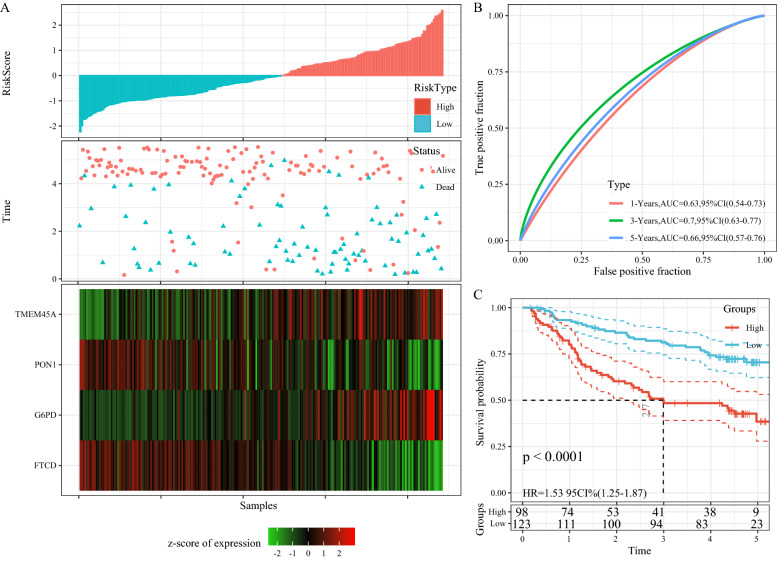
**A** RiskScore, TTL, survival status and 4-gene expression in the independent validation dataset GSE14520; **B** ROC curve and AUC of 4-gene signature classification; **C** KM survival curve distribution of 4-gene signature in the independent validation dataset GSE14520

The distribution of RiskScore for independent validation dataset GSE14520 (Fig. 8A) suggested that high RiskScore samples have a worse prognosis and the prognostic prediction efficiency of Riskscore at 1 year, 3 years, and 5 years was analyzed using the R software package timeROC (Fig. 8B). The samples having Riskscore greater than zero and less than zero were classified as high-risk group and low-risk group respectively. The plotted KM curves showed a significant difference, with 98 samples being classified as a high-risk group and 123 samples as a low-risk group (Fig. 8C, p < 0.01).

**Fig. 9:**
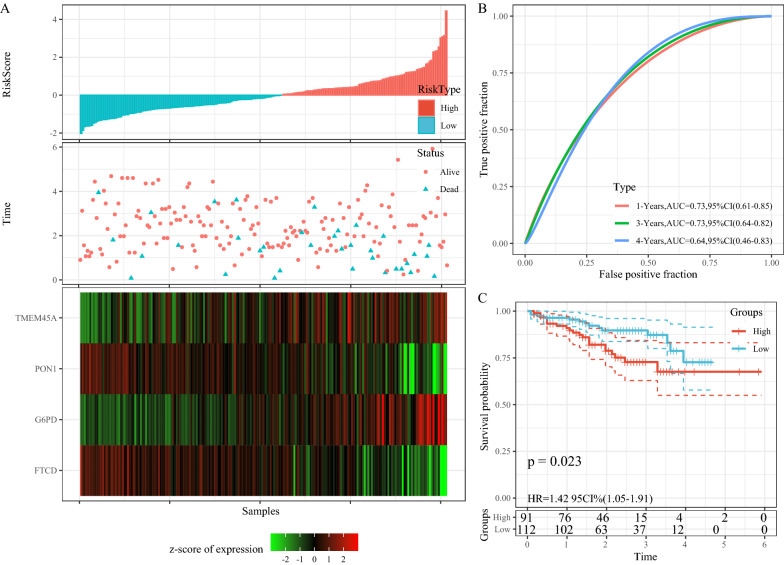
**A** RiskScore, TTL, survival status and 4-gene expression in the independent validation dataset HCCDB18; **B** ROC curve and AUC of 4-gene signature classification; **C** KM survival curve distribution of 4-gene signature in the independent validation dataset HCCDB18

The distribution of RiskScore for the independent validation dataset HCCDB18 is shown in Fig. 9A. The ROC analysis of RiskScore for prognostic classification was performed using the R software package timeROC. With very few 5-year survival samples from this dataset, only the prognostic prediction of Riskscore at 1 year, 3 years, and 4 years was analyzed (Fig. 9B). The plotted KM curves showed a significant difference, with 91 samples were classified as high-risk group and 112 samples as a low-risk group (Fig. 9C, p < 0.01).

### Correlation analysis of risk model having clinical features and pathways

**Fig. 10:**
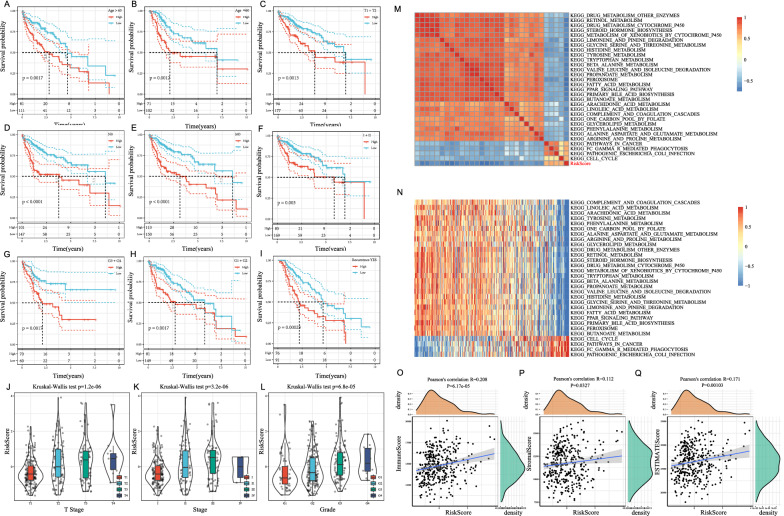
Prognostic performance of risk models reflective of different clinical features. **A** Based on Riskscore, patients in the Age > 60 group can be divided into two groups with significant prognosis; **B** Based on Riskscore, patients with Age ≤ 60 can be divided into two groups with significant prognosis; **C** Based on Riskscore, patients in the T1+T2 group can be divided into two groups with significant prognosis; **D** Based on Riskscore, patients in the N0 group can be divided into two groups with significant prognosis; **E** Based on Riskscore, patients in the M0 group can be divided into two groups with significant prognosis; **F** Based on Riskscore, patients in Stage I+II group can be divided into two groups with significant prognosis; **G** Based on Riskscore, patients in the Grade3+4 group can be divided into two groups with significant prognosis; **H** Based on Riskscore, patients in the Grade 1+2 group can be divided into two groups with significant prognosis; **I** Based on Riskscore, patients in the Recurrence_Yes group can be divided into two groups with significant prognosis

A 4-gene signature showed it could significantly distinguish the Age, T Stage, N Stage, M Stage, Stage, Grade, and Recurrence groups from the two high-and low-risk groups, respectively (Fig. 10A−I, p < 0.05), thus suggestive of the model’s predictive ability. After comparing the distribution of RiskScore amongst clinical groups, significant differences were found among T Stage, Stage, Grade, and Recurrence groups (Fig. 10J−L, p < 0.05), and a more advanced stage correlated with a higher RiskScore. The same is seen with Grade samples, where the higher degree of differentiation correlated with a higher RiskScore and RiskScores of Recurrence samples were higher than the Non-recurrence samples.

The relationship between RiskScores and biological functions of different samples are observed through corresponding gene expression profiles that were subjected to single-sample GSEA. The ssGSEA scores for each function were calculated for each sample. Then, the correlation between these functions and the RiskScore was further calculated, and a correlation greater than 0.55 was selected in Fig. 10M (4 showing a positive RiskScore correlation and 26 showing a negative RiskScore correlation). The 30 most relevant KEGG Pathways were selected and clustered (Fig. 10N), and those tumor-related pathways: KEGG_PATHWAYS_IN_CANCER and KEGG_CELL_CYCLE increased with increasing RiskScore, and metabolism-related pathways: KEGG_FATTY_ACID_METABOLISM, KEGG_RETINOL_METABOLISM, KEGG_PPAR_SIGNALING_PATHWAY, KEGG_DRUG_METABOLISM_CYTOCHROME_P450 decreased with increasing RiskScore.

The relationship between the immune and matrix scores of the RiskScore was established using the R software package estimate and Pearson correlation coefficient. The calculated values showed that the RiskScore and the StromalScore, ImmuneScore, as well as ESTIMATEScore all showed a significant positive correlation (Fig. 10O−Q, P < 0.05).

### Univariate and multivariate analysis of 4-gene signature

**Fig. 11:**
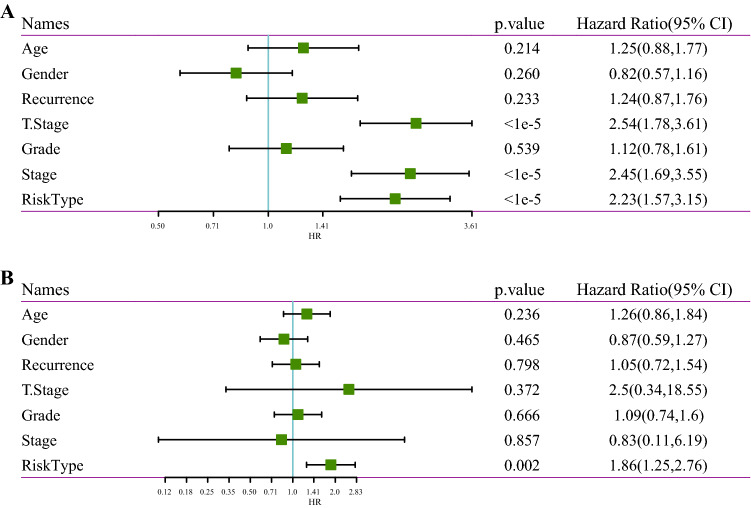
**A** Forest plot of univariate cox regression analysis; **B** forest plot of multivariate cox regression analysis

Clinical independence of 4-gene signature in the TCGA dataset was identified using univariate and multivariate COX regression analysis, and the univariate COX regression analysis found that RiskScore was significantly associated with survival and the multivariate COX regression analysis revealed that RiskScore (HR = 1.86, 95% CI = 1.25−2.76, p = 0.002) remained significantly associated with survival (Fig. 11). The above results indicated that our 4-gene signature model possesses a good clinical predictive performance.

### The comparison of risk model with other models

**Fig. 12:**
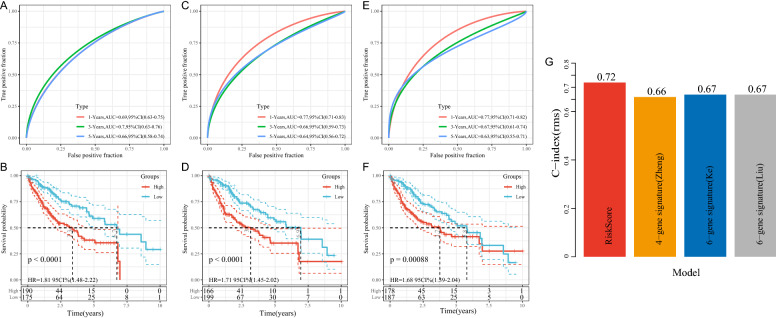
**A**, **B** ROC of the 4-gene signature (Zheng) risk model and LIHC KM curves of the High/Low group samples; **C**, **D** ROC of the 6-gene signature (Ke) risk model and LIHC KM curves of the High/Low group samples; **E**, **F** ROC of the 6-gene signature (Liu) risk model and LIHC KM curves of the High/Low group samples; **G** C-index for the 4 prognostic risk models

By retrieving the literature, three prognosis-related risk models, namely 4-gene signature (Zheng) [30], 6-gene signature (Ke) [31] and 6-gene signature (Liu) [32] were finally selected for comparison with our 4-gene model. To make models comparable, RiskScores were calculated for each LIHC sample based on the corresponding genes in 3 models. The Riskscore greater than zero were classified as a high-risk group and those with less than zero were classified as a low-risk group. Survival analysis revealed that the prognosis of LIHC differed between the high- and low groups of the three models (Fig. 12B, D, F, log-rank p < 0.05), their 5-year AUC values, however, were lower compared to our model (Fig. 12A, C, E), highlighting the more reasonable and effective result with a reasonable number of genes.

To compare the predictive performance of these models to the LIHC sample, the concordance index (C-index) of the four model was calculated using the rms package in R. The result showed that our RiskScore model yielded a higher C-index value thus indicating a good predictive performance.

### Prediction of immunotherapy by the risk model

With the limited effective predictive markers for immunotherapy, there is a critical need to identify novel predictive markers to further advance precision immunotherapy. Imvigor210, an immunotherapy dataset containing transcriptomic data, was retrieved to explore if the 4-genes model could predict the benefit of immunotherapy. This resulted in imvigor210 recording expression data from patients who responded and failed to respond with anti-PD-L1 immunotherapy.

**Fig. 13:**
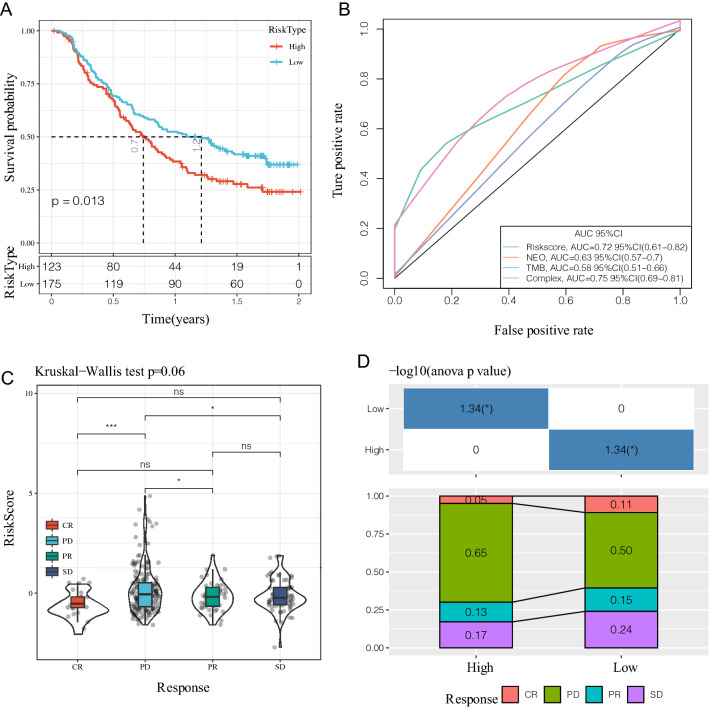
**A** KM curve of Imvigor210 dataset; **B** ROC curve of Imvigor210 dataset; **C** differences of RiskScore between effectiveness groups of immunotherapy; **D** corresponding stacked plots of immunotherapy between different groups of the Imvigor210 dataset

Kaplan−Meier curves showed that higher RiskScore values were associated with poorer survival in mUC patients receiving immunotherapy (Fig. 13A) whereas ROC analysis revealed that the complex model integrating Riskscore, NEO, and TMB had higher predictive performance (Fig. 13B, ROC = 0.75). Comparing the RiskScore differences between different groups also showed that patients belonging in the complete response (CR) group had a significantly lower RiskScore than those in the progressive disease (PD) group (Fig. 13C) and that fewer samples were responding to immunotherapy (CR + PR) in the high-risk group than in the low-risk group (18% vs. 26%) (Fig. 13D).

### Immunohistochemical verification of genes in the model

**Fig. 14:**
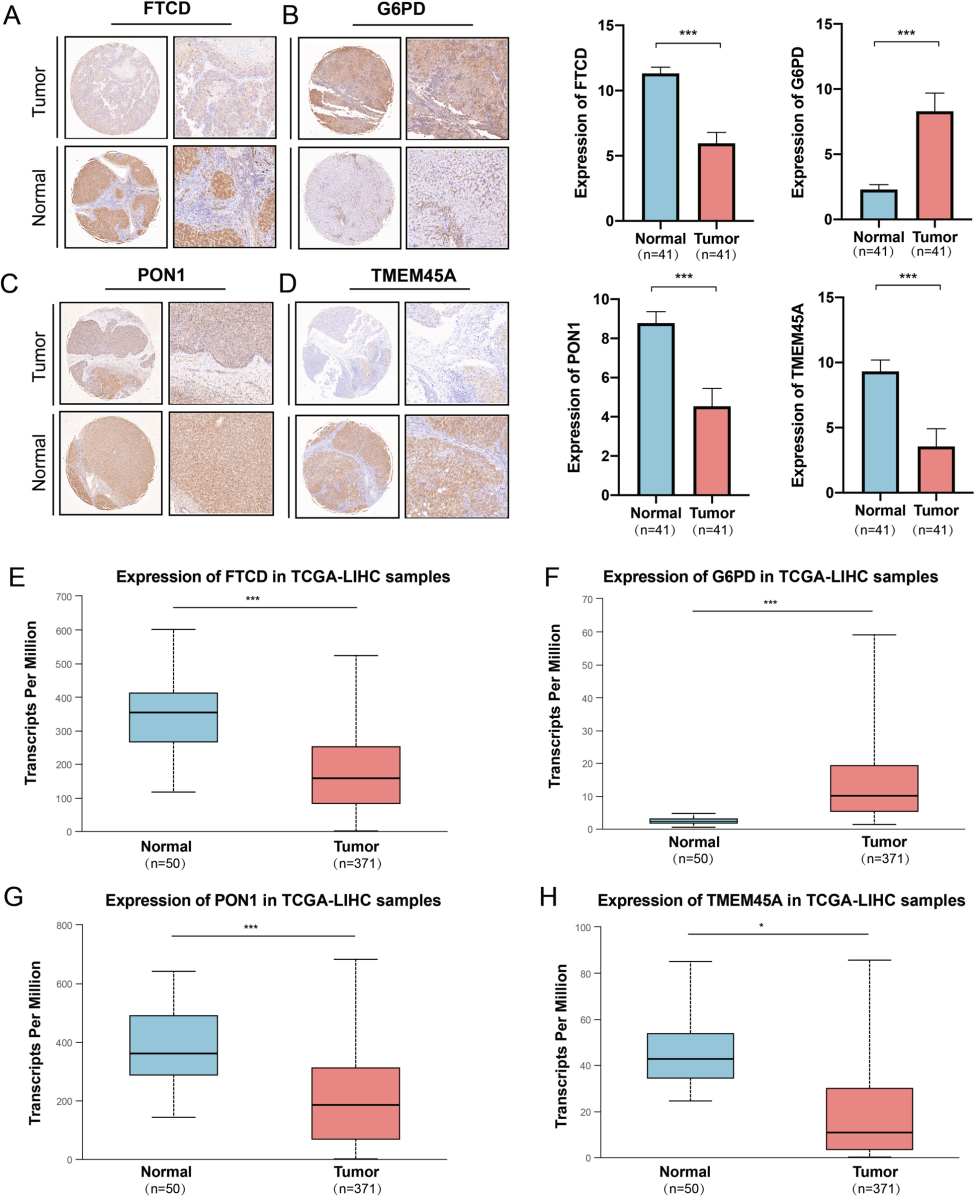
Gene expression amongst the liver cancer cohort. **A**−**D** Immunohistochemical expression of FTCD, G6PD, PON1, and TMEM45A genes in the real cohort; **E**−**H** transcriptome expression levels of FTCD, G6PD, PON1, and TMEM45A genes in the TCGA HCC cohort, respectively

To verify the expression differences of FTCD, G6PD, PON1 and TMEM45A, protein expressions in 82 cases of HCC and para-cancerous tissues was detected using immunohistochemical assays and reflected higher expressions of FTCD, PON1, and TMEM45A genes in para cancerous tissues (Fig. 14A, C, D) and higher expressions of G6PD genes in cancerous tissues (Fig. 14B). It is important to make mention that the transcript expression of the above genes analyzed in the UALCAN (http://ualcan.path.uab.edu/) database was consistent with the data presented in the study (Fig. 14E−H).

## Discussion

Patients with HCC usually lack clinically significant symptoms in the early stages of the disease. Owing to the high morbidity and mortality associated with the disease, these patients remain a major public health challenge worldwide [33]. Given the enormous heterogeneity of HCC, the identification of new prognostic markers and the construction of more accurate prognostic models are crucial. In this study, we classified 365 LIHC samples from TCGA based on the 59 EMT-associated genes identified, and assigned them to two molecular subtypes which had different clinical features and prognostic outcomes. In general, the C1 group showed poor prognosis and had a higher proportion of deaths, higher T-stage, higher differentiation degree, more advanced staging, and higher immune scores, compared to the C2 group. Based on this, a prognostic evaluation model was constructed, which not only distinguished the different molecular subtypes but also better evaluated the prognosis of patients with HCC.

In recent years, increasing number of studies have reported on tumor prognostic models. However, no studies have focused on predicting HCC prognosis based on EMT-associated markers. In this study, we fabricated a novel, highly robust four-gene marker (including *PON1*, *FTCD*, *G6PD*, and *TMEM45A*) for the prediction of HCC prognosis, based on EMT-associated genes, and validated the marker in two other independent cohorts. Paraoxonase-1 (PON1), a Ca^2+^-dependent high-density lipoprotein (HDL)-associated endostatin, is the first member of the paraoxonase (PON) multigene family. It is associated with the antioxidant effect of HDL, possesses atheroprotective properties, and is associated with the pathogenesis of many diseases, including cardiovascular disease and cancer [34–36]. A number of studies have shown that PON1 activity is associated with the progression of many cancers [37, 38]. For example, *PON1* gene polymorphism has been associated with breast cancer susceptibility [39], while *PON1* concentration has been positively correlated with the degree of bone destruction in multiple myeloma [40]. Moreover, PON1 activity is elevated in the serum of patients with colorectal cancer and tissues of patients with colon cancer [41]. In addition, since serum PON1 concentration is significantly reduced following radiotherapy, it can be used as an indicator of radiotherapy efficacy [42, 43]. PON1 has also been extensively studied in HCC [44–48]. Currently, serum PON1 is used as a biomarker to assess microvascular infiltration in HCC [49, 50].

Formimidoyl transferase cyclodeaminase (FTCD) catalyzes the degradation of histidine during folate metabolism. In addition, it has also been found associated with the Golgi complex [51]. The *FTCD* gene, a candidate tumor suppressor gene in HCC [52], is significantly downregulated in HCC tumor tissues. Therefore, it serves as a useful diagnostic biomarker for distinguishing early stage HCC from benign tumors [53]. Moreover, most hepatocellular and metastatic cancers are diagnosed by examining the combined expression of arginase 1 + FTCD + MOC 31 [54]. In addition, FTCD has also been found to correlate with drug sensitivity to methotrexate chemotherapy [55].

Glucose-6-phosphate dehydrogenase (G6PD) is the rate-limiting enzyme of the pentose phosphate pathway, and its deficiency results in one of the most commonly inherited enzyme deficiency disorders. Reduced NADPH, which is produced by G6PD, is essential for the maintenance of intracellular redox homeostasis and reductive biosynthesis [56]. G6PD is frequently activated in human malignancies to produce precursors for nucleotide and lipid synthesis. The abnormal activation of G6PD leads to proliferation of a variety of cancer cells [57]. G6PD activity is increased in several cancer types, including esophageal, gastric, colorectal, bladder, breast, and lung cancers [58–60]. Increased expression levels of G6PD mRNA results in poor clinical outcomes in cancer patients, including increased drug resistance, and tumor cell migration or proliferation. Therefore, G6PD has been predicted as a valuable potential target for cancer therapy in the near future [61]. In particular, one study has identified G6PD as an important miR-122 target that regulates glucose metabolism in HCC. In addition, the upregulation of G6PD has been reported to be associated with higher tumor grade, increased tumor recurrence, and poor survival in patients with HCC [62].

TMEM45A is a member of the transmembrane protein (TMEM) family. These proteins are components of various cell membranes including the mitochondrial, endoplasmic reticulum, and Golgi membranes [63]. TMEM45A has been reported to be associated with chemotherapy resistance in human breast cancer and HCC cells under hypoxic conditions. It also affects the proliferation and invasion of human ovarian cancer and glioma cells [64–67]. *TMEM45A* gene knockdown has been found to be effective in inhibiting multidrug resistance and suppressing EMT by inhibiting the TGF-β signaling pathway in human colorectal cancer cells [68]. These studies suggest that TMEM45A may be a potential biomarker. In our study, we found that the four-gene marker construct was involved in a wide range of tumorigenic processes and was closely associated with HCC tumor cell growth, metastasis, or invasion, thereby making the four-gene signature construct a powerful biomarker for the prediction of HCC prognosis.

Evaluation of the four-gene construct using GSEA showed certain significantly enriched tumor features and various metabolic features. The analysis revealed that a large number of tumor-related pathways were significantly overexpressed in subtype C1, suggesting that the tumors of this subtype are more aggressive. This finding is also consistent with the clinical features of the tumors of C1 subtype such as late stage tumor, high degree of differentiation, and high mortality. On the other hand, the expression levels of metabolism-related pathways were higher in the C2 subtype compared to C1, and most of these metabolic pathways were related to physiological hepatocyte metabolic functions such as fatty acid metabolism, PPAR signaling pathway, and drug metabolic processes. This indicates a more intact hepatocyte function, thereby contributing to a better clinical outcome compared to the C1 subtype. Besides, we found that the tumor-related pathways increased with increasing RiskScore, while the metabolism-related pathways decreased with increasing RiskScore, which indicates that RiskScores can help predict the prognosis of HCC and aid in better understanding the molecular mechanisms underlying HCC onset and progression.

Three published gene signatures of HCC were compared to demonstrate the superiority of our model. Using a four-gene signature marker (Zheng) [30], we obtained 149 pairs of HCC specimens from GEO and identified 98 DEGs between HCC and normal hepatic tissues. Subsequently, we established and validated a four-gene subset of prognostic gene expression signature markers for HCC (*SPINK1*, *TXNRD1*, *LCAT*, and *PZP*). We found that the expression panel of these four genes strongly correlated with the methylation status of the genes. Another six-gene signature (Ke) marker [31] helped identify two prognostic molecular subtypes of HCC with different expression profiles and clinical outcomes. It also helped establish a prognostic evaluation model that not only distinguished the different subtypes of HCC, but also provided a good evaluation of patient prognosis. Another six-gene signature (Liu) marker [32], which included *CSE1L*, *CSTB*, *MTHFR*, *DAGLA*, *MMP10*, and *GYS2*, helped classify HCC patients into high- and low-risk groups with significant differences in their survival rates. ROC analysis of the four models showed that the 5-year AUC values of the four-gene signature (Zheng), six-gene signature (Ke), and six-gene signature (Liu) markers were lower than that obtained in our model, indicating that our model is more reasonable and effective with similar number of genes. Besides, the C-index values of our RiskScore model were higher than those of the other three models, proving the good performance of our model.

Researchers have identified several genetic markers associated with cancer immunotherapy responsiveness, such as PD-L1 expression, tumor mutation burden (TMB), and DNA mismatch repair defects [69–71]. In 2020, the NCCN guidelines prioritized the use of atezolizumab and bevacizumab combination therapy [5]. However, the current use of immunotherapy for HCC is limited owing to the limited number of effective predictive markers [72, 73]. Therefore, we explored the ability of our four-gene model to predict immunotherapeutic efficacy by using an immunotherapy dataset (Imvigor210). We found that patients in the CR group had a significantly lower RiskScore compared to the PD group. We also found that higher RiskScore values were associated with poorer survival, and that the proportion of samples with immunotherapy response (CR + PR) was smaller in the high-risk group compared to the low-risk group (18% vs. 26%). In short, HCC patients from the high RiskScore group respond poorly to immunotherapy. However, this needs to be verified in the future via clinical trials.

Despite the promising findings obtained, our study has certain limitations. Firstly, our findings were based on a single platform and the study was retrospective. Data from different centers and different platforms are needed to further verify the performance of our model. Secondly, the limited sample size could have resulted in selection bias. Thirdly, our process of screening for differential genes was mainly based on statistics. Consequently, certain biologically significant genes may have been overlooked. Finally, the four genes identified in this study need to be subjected to more in-depth cellular experiments and animal studies to further explore their role in EMT, which will aid in laying the foundation for clinical applications.

## Conclusions

In this study, a four-gene signature (*PON1*, *FTCD*, *G6PD*, and *TMEM45A*) prognostic stratification system was constructed based on the EMT-associated genes of HCC cells, to effectively predict HCC prognosis. Additionally, the stability and accuracy of the model were evaluated. Our results reveal that the performance of our model is superior compared to that of the currently existing models. Therefore, through this study, we propose the use of this classifier as a molecular diagnostic marker for the evaluation of the prognostic risk of patients with HCC.

## Supplementary Information

**Additional file 1: Table S1.** 59 EMT genes related to the prognosis of liver cancer

**Additional file 2: Table S2.** 1130 differentially expressed genes between molecular subtypes

**Additional file 3: Table S3.** 71 differentially expressed genes related to prognosis among molecular subtypes

## Data Availability

The data used to support the findings of this study are available from the corresponding author on reasonable request.

## Abbreviations

EMT: Epithelial−mesenchymal transition
HCC: Hepatocellular carcinoma
DEGs: Differentially expressed genes
LIHC: Liver hepatocellular carcinoma
GEO: Gene Expression Omnibus
BSA: Bovine serum albumin
CR: Complete response
PD: Progressive disease
G6PD: Glucose-6-phosphate dehydrogenase
TMEM: Transmembrane protein
