# Supplementary material for: Identification and clinical validation of EMT-associated prognostic features based on hepatocellular carcinoma
Source: Cancer Cell Int. 2021 Nov 24;21:621. doi: 10.1186/s12935-021-02326-8 (PMC8613962; doi:10.1186/s12935-021-02326-8)
Supplement: Supplementary file 1 — Additional file 1: Table S1. 59 EMT genes related to the prognosis of liver cancer [file 12935_2021_2326_MOESM1_ESM.docx]

"","p.value","HR","Low 95%CI","High 95%CI"

"SERPINE1",0.0154172903263272,1.11768697716157,1.02146791963665,1.22296956654392

"VCAN",0.0325820331754115,1.12189351617394,1.00958377544964,1.24669699755482

"IGFBP3",0.0061438224134876,1.17745338234275,1.04760288464748,1.32339886411912

"LAMC1",0.0181342621893274,1.19413558843968,1.03071527778113,1.38346625330703

"LOX",0.0020835596936134,1.22934330134281,1.07788749154464,1.40208042528699

"LOXL2",0.0419818081199919,1.20523706681806,1.00679133203915,1.44279786784627

"ITGAV",0.000928063398850675,1.2557258479167,1.09739219587526,1.43690415427862

"SERPINH1",0.0154262398953065,1.21576457525595,1.03798841274793,1.42398844177292

"POSTN",0.0490659885241542,1.09852991219657,1.00038464409286,1.20630397029423

"SPP1",2.69075435397561e-06,1.13113612360283,1.07439927165959,1.19086913391413

"ITGB5",0.00157231449156096,1.41753052523914,1.14176113223698,1.75990645788395

"MMP3",0.00166407514860154,1.40651494910424,1.13711456510134,1.73974053518292

"FBLN5",0.0158942279147337,0.860718839499235,0.761931192222902,0.972314728981695

"MAGEE1",0.0018633130494652,1.47913873646631,1.15586784599198,1.89282140627202

"SGCB",0.000960523548005605,1.26571846857621,1.10050009924226,1.45574111515117

"DAB2",4.0850897304751e-05,1.32877402916274,1.16004316672838,1.52204717136255

"ITGA5",0.00161641543303671,1.3097802280876,1.10750074669897,1.54900504672572

"VEGFA",0.00334881877998082,1.32977977731051,1.09922334038109,1.60869423999942

"CXCL1",0.00442168074432141,1.10757270214513,1.03232999679617,1.18829956926969

"PLOD2",2.3764965727086e-06,1.48540893215265,1.26027143243236,1.75076546126289

"TNFRSF11B",0.00239454100565711,1.1782765617643,1.05988142399905,1.30989714940446

"SERPINE2",0.0103759539813503,1.15573809351833,1.03464337019292,1.29100575066781

"GPC1",0.00056423546332226,1.26545327870318,1.10695514634985,1.44664578854989

"LAMA1",0.032996459390255,1.23928296117342,1.01747376067175,1.50944655009166

"FBN2",0.00143373813867082,2.06954457107648,1.32331388105945,3.23658263770583

"PFN2",4.30010422465592e-06,1.27716549957606,1.1506447634612,1.41759799818737

"GADD45B",0.0421139598870269,0.859372983652856,0.742521370260311,0.994613696806467

"CXCL8",0.000360059797294167,1.15141477469865,1.06559888516326,1.24414167643505

"P3H1",2.73066333218185e-08,1.93436380283925,1.53282185821935,2.44109470495251

"MMP14",0.00344052455761746,1.19724431187225,1.06120951301021,1.35071720026756

"PLOD1",0.000852838423174599,1.44022945213474,1.16231418497232,1.78459568128366

"MATN3",0.00301295118126531,1.17504363985289,1.05625318508278,1.30719374394173

"SLC6A8",0.0267364607201999,1.10780814815676,1.01187246264047,1.21283950145262

"CALU",0.000311191327301234,1.41596459095102,1.17205118053123,1.71063837154138

"TNFRSF12A",0.0209219528496938,1.17615561464703,1.02485349768938,1.34979490530559

"PLAUR",0.00165169387315809,1.2592452994156,1.09082152439461,1.45367384914807

"TGFB1",0.00875415510156853,1.17663427382886,1.04190127109998,1.32879021530238

"PLOD3",0.00759404788662135,1.35323130636398,1.08373137709573,1.68974988380521

"APLP1",0.0290435961917011,1.13280583014889,1.01281630197163,1.26701065762985

"FBLN2",0.0348260509159293,0.890815242874438,0.800117976460275,0.99179348581583

"MSX1",0.0306065801932187,1.21554476829957,1.01842037859964,1.4508243499331

"PTX3",0.0225507148534028,1.22324379086362,1.02876369300024,1.45448889970307

"TPM4",0.0310028950148557,1.18463362161717,1.0156002584303,1.38180037452416

"IGFBP4",0.00834816953471108,0.77046947938917,0.634755835358565,0.935199309093219

"COPA",0.003020141104501,1.40233583020233,1.12150240350258,1.75349225692919

"ITGA2",0.0133562739007209,1.19967088011398,1.03855698709587,1.38577876657296

"LGALS1",0.00816248383331764,1.19952479330801,1.04825968417463,1.37261763614762

"NT5E",0.0485119672774623,1.15128635678786,1.00092103343965,1.32424060544612

"CTHRC1",0.000810974586314723,1.1746542752058,1.06904567826989,1.29069570581147

"ECM2",0.00137481757947555,0.79230986499119,0.687016597687021,0.913740547573116

"MCM7",0.000262132599071982,1.33717546572846,1.14401089811865,1.56295558817368

"OXTR",0.0121591359333642,1.20196595894845,1.04098626432462,1.38783979768282

"MMP1",8.31619701968203e-08,1.38244054018088,1.22805617587084,1.55623324460737

"CAPG",0.000831630815524687,1.19888001579592,1.07789687021959,1.33344230972859

"ENO2",0.00289071835733775,1.19801449642723,1.06375583515848,1.34921820046793

"BDNF",0.00455232986461287,1.64797672657125,1.1670104489675,2.32716621665495

"ADAM12",0.0196618069964002,1.20755828250811,1.03059742791596,1.4149045652119

"COLGALT1",8.38813862542404e-06,1.68794030611426,1.34070333539513,2.12511030724438

"GPX7",0.0071673473255653,1.17272270160577,1.0441417863287,1.31713772293048
