# Supplementary material for: Identification and clinical validation of EMT-associated prognostic features based on hepatocellular carcinoma
Source: Cancer Cell Int. 2021 Nov 24;21:621. doi: 10.1186/s12935-021-02326-8 (PMC8613962; doi:10.1186/s12935-021-02326-8)
Supplement: Supplementary file 2 — Additional file 2: Table S2. 1130 differentially expressed genes between molecular subtypes [file 12935_2021_2326_MOESM2_ESM.docx]

"","logFC","AveExpr","t","P.Value","adj.P.Val","B"

"SPP1",3.71796025470499,6.75536575471398,11.4825305243213,2.77470719525815e-26,6.8419654923074e-25,48.9026537996264

"MMP7",2.97730964416949,2.10489982908401,15.5019534357683,4.75539971563837e-42,2.51271924260249e-39,84.8365948806098

"CXCL1",2.95483001091372,2.38717347240413,15.4918399239778,5.23001619791173e-42,2.5792696549368e-39,84.7423679634066

"PTGDS",2.84158202523188,3.7773919005149,10.2775164280013,6.05548035224674e-22,8.42811211773193e-21,39.0319641677633

"LUM",2.8050730424113,3.63133497860974,12.7030580715202,6.76952937954466e-31,3.25179179124556e-29,59.4083612793192

"COL1A1",2.79158916768662,5.6765514210294,15.3907036777843,1.35321252623862e-41,5.41102143937846e-39,83.8007821903829

"CXCL8",2.7713028203534,2.83347690716562,15.4956131943785,5.04764723597293e-42,2.57517037435239e-39,84.7775218159265

"POSTN",2.68880889349437,2.31027403556338,18.5014968231727,1.82529209873336e-54,2.70051966007601e-50,113.152516548666

"COL3A1",2.6047529440057,5.75903399713186,14.7212571452456,7.0645888111883e-39,1.56000882778404e-36,77.6030274824238

"KRT19",2.57334691976418,2.23008985249464,10.9144589303304,3.29884240240954e-24,6.11608688516907e-23,44.1801180152771

"CRP",2.56523159738342,8.37252236582227,6.56544220736317,1.7735800440067e-10,5.52656207899729e-10,13.077548879468

"ISLR",2.48979869866775,2.57886206598791,15.1704291994569,1.06808992961748e-40,3.59145238833877e-38,81.7545511658398

"COL1A2",2.4542201210752,4.94668791303013,15.4021994787293,1.21468951715507e-41,5.13466611608837e-39,83.9077446193091

"SPINT1",2.4281421868961,2.67785783814977,11.1016211037114,6.9219348059586e-25,1.3933336796484e-23,45.7230723643751

"CXCL6",2.40225282087993,1.67836013250253,13.4550794326014,7.95171198810078e-34,6.53586549244172e-32,66.0861283102791

"MSC",2.36070969428067,2.62172857985823,12.5249971934197,3.27527109631951e-30,1.41041092286798e-28,57.8483423308701

"CTHRC1",2.34220660910687,2.56882968234949,15.6959593514869,7.64899363165196e-43,4.71528586584545e-40,86.6464892700227

"TESC",2.33133494073554,3.61634466873181,11.2713983305709,1.6607118283316e-25,3.63464963020207e-24,47.1338801857162

"TIMP1",2.31816923589827,7.96996381671623,12.7903881953787,3.11469974485908e-31,1.59453227422803e-29,60.1765800132635

"VCAN",2.31738359193998,1.92305548393809,18.2271339967073,2.54159097277446e-53,1.88014192210991e-49,110.544328619655

"THBS2",2.2863360375122,3.1417332433121,13.5489516363867,3.39375799442125e-34,3.02473792334111e-32,66.9290361139776

"SPINK1",2.23032543000542,6.28885207638252,5.74231653770432,1.96013943142529e-08,4.65493786323229e-08,8.5001480368823

"PKM",2.22953704236989,4.93270186196732,16.268482789532,3.39917940045328e-45,4.19090493580886e-42,92.011271399452

"COL5A1",2.2101007366099,3.01369820798276,15.8580385889233,1.65649449971763e-43,1.1670398153963e-40,88.1618166583751

"CA9",2.19134069897822,1.70382157893271,9.57950072874873,1.50602406840762e-19,1.5658205264997e-18,33.5892778568401

"S100A6",2.19026151412143,6.77876992655615,13.8501955856809,2.18037506236163e-35,2.35464591588615e-33,69.6467400943112

"HTRA3",2.17699061864115,2.09865540713992,17.0492007356719,2.01205353989236e-48,6.22565414234093e-45,99.3728823593517

"AEBP1",2.15248034418116,4.26615398258289,11.7254580198114,3.47552538731465e-27,9.61128936548042e-26,50.9566683169265

"MMP2",2.13729929004448,3.44156525841481,14.4389350805051,9.69174265773471e-38,1.52541843214027e-35,75.0096252729102

"FMOD",2.11283560784686,2.70373842869037,14.3379404182276,2.46514440293244e-37,3.54095256712481e-35,74.0851493701068

"CCL21",2.09935984236581,4.09936459221734,8.03440774055797,1.29682768274667e-14,7.07469231793399e-14,22.4038417006024

"BGN",2.0938216565939,7.0756841476827,12.9668856089887,6.4482428170729e-32,3.6552395585668e-30,61.7352154341277

"EFEMP1",2.09133789700491,3.10551641037277,10.5005505716141,9.94179374654466e-23,1.52740226874484e-21,40.8159440602196

"BICC1",2.08991182026867,2.29137281576074,13.1397781300984,1.36828293224324e-32,9.07791299665417e-31,63.2695907449894

"SOD3",2.08698113862497,3.27392863701973,12.9094063059564,1.07786621561106e-31,5.86287891910499e-30,61.2267469368334

"QSOX1",2.07826464306417,3.69794998031258,11.9122479534573,6.94474993176277e-28,2.10898901445908e-26,52.549198866617

"SEL1L3",2.07656159138644,2.91968202266139,14.3019088966725,3.43797651046615e-37,4.70970948818025e-35,73.7557599255453

"FDCSP",2.07369544406633,1.29848906145902,9.45201599888886,4.02697432116858e-19,3.96137533787826e-18,32.6195673211342

"SULF1",2.07142819559278,1.87100364905159,17.0445216751581,2.10397233603952e-48,6.22565414234093e-45,99.3286355994201

"CD24",2.06678560299144,5.39960678063198,7.93337908949359,2.60542147751376e-14,1.35729615351465e-13,21.7187117734381

"PMEPA1",2.0593042530312,2.45906125474672,15.1043181054942,1.98310834308735e-40,6.11251831999527e-38,81.141684009454

"S100A11",2.05642124801255,7.27484508274079,15.329397304969,2.40636343715354e-41,9.36898606649649e-39,83.230642284043

"RGS2",2.0539278820685,3.69728272961946,15.209730948847,7.39137066843653e-41,2.60369831046473e-38,82.1191720768126

"PFKFB3",2.03575217624137,3.20749631287452,14.6710654381182,1.12639669977653e-38,2.4152230685788e-36,77.1410191891493

"MGP",2.03564997005729,4.51907123013057,12.6743255253126,8.73558056831051e-31,4.08996564899221e-29,59.1560510360594

"MMP9",2.03528915055575,3.1560008123636,11.2501028073567,1.98746118076231e-25,4.30519592523843e-24,46.9563484510556

"CCL20",2.0158445087183,4.68053025806296,8.32200569259531,1.72334613842492e-15,1.05577251006198e-14,24.3870593628459

"IER3",1.98931137997852,5.22073086776407,13.4288549773302,1.00835399090559e-33,8.10793331274359e-32,65.8509999595787

"MFAP4",1.97717883117615,2.96157981701333,9.68846404741996,6.45831116040796e-20,7.00005227972423e-19,34.4242482206777

"EPCAM",1.97379201678384,2.7174408698797,6.45293862523898,3.47047198149856e-10,1.0389646492568e-09,12.4228689237329

"NTS",1.96859646429117,1.49798562993192,7.27838999044336,2.06845881613965e-12,8.3250403114217e-12,17.4290277489138

"SPHK1",1.95667495475609,2.42224354064624,10.997428828977,1.65362099962851e-24,3.1731936043455e-23,44.8624963328921

"UCHL1",1.95450638063384,1.5761374912485,10.6089213105005,4.10224012380951e-23,6.64033289187765e-22,41.6901772747577

"CCL19",1.94390141878815,3.01374672496894,8.07656850089399,9.67553847110335e-15,5.34539177296393e-14,22.6915503438468

"C12orf75",1.94173212587517,2.83875569030119,11.9401794806504,5.45340460504605e-28,1.68089835690951e-26,52.788291814656

"RGS1",1.93911926912591,2.90789133757656,13.0746469380661,2.45570222203685e-32,1.54604742021426e-30,62.6907078763497

"FLNA",1.90841295076719,5.10144388274873,15.4405143382929,8.47414156836098e-42,3.79924013648184e-39,84.2643642192963

"SERPINE2",1.9015471005595,2.3090119799798,13.9795852421929,6.6683948451282e-36,7.58914628720551e-34,70.8197441305691

"HSPB8",1.8852447946276,3.00978650476786,10.1935064037193,1.18955223068311e-21,1.58268212706445e-20,38.3654428682437

"DCN",1.88509866127918,2.74288695946244,10.0854019544238,2.82357496892359e-21,3.5858190270579e-20,37.5122497787831

"CXCL5",1.88426698915696,0.995028058634178,11.3300340854044,1.01194476619086e-25,2.28582969144686e-24,47.6235360578919

"TRNP1",1.88380052036405,3.61194282209379,9.52953236497991,2.21642047784516e-19,2.25219374792027e-18,33.2082632603436

"PFKP",1.87376051455688,2.55224575076919,13.43583064083,9.46631887888465e-34,7.65323430672669e-32,65.9135286996655

"SLC1A5",1.87232798029374,3.58195943062649,12.9450522102447,7.83856638712003e-32,4.34350523211389e-30,61.5419765351089

"SOX4",1.87138906987289,3.39748840877487,12.3805317708203,1.16946099433959e-29,4.66638608508206e-28,56.5890939444479

"GEM",1.86395222186921,2.50631002201705,14.7367100273544,6.11892148803093e-39,1.37165823356693e-36,77.7453494556814

"B3GNT3",1.86236847279037,2.93153108794284,9.16838962323082,3.49223432935903e-18,3.04285081877896e-17,30.4907156801966

"EMILIN1",1.85679666695402,3.64283168434464,12.6565851055319,1.02238550776886e-30,4.72693549607507e-29,59.0003767437191

"FKBP10",1.85158879672527,3.32570320529424,12.5366653110656,2.95457094163544e-30,1.28567285533813e-28,57.950303413312

"CCDC80",1.85158176777837,1.9543453280409,13.19226661867,8.53414461522612e-33,5.87268230615211e-31,63.7368497426836

"NQO1",1.85008715048708,4.63954977905321,6.07847302974604,3.04179923340612e-09,8.05070119109904e-09,10.3092935870637

"KRT23",1.84914840687506,2.92983552435338,6.77870745456937,4.85190512207196e-11,1.63154936344098e-10,14.3429418134368

"SFRP5",1.84874140926943,1.2436517777927,9.03037451122191,9.84815750662807e-18,8.15809016296541e-17,29.4694413764593

"TGFB1",1.84568079795386,4.3251415858966,14.8843631703615,1.54709928898538e-39,3.94643689319632e-37,79.1071056719485

"BACE2",1.83829003106769,2.25337605031328,11.364030688811,7.58897072212779e-26,1.74345996636461e-24,47.9079918415924

"LIF",1.83071838760407,1.64104245343792,14.5751452360203,2.7440797652376e-38,5.07483251583628e-36,76.2592116120557

"AGR2",1.79924973603155,1.2855955755025,8.1966613360486,4.17732027617225e-15,2.44282424845725e-14,23.5167985490362

"COL6A3",1.7880231535569,2.90801423044973,14.5686894482847,2.9133999966415e-38,5.2565552378428e-36,76.1999165732399

"TMEM132A",1.78156409574034,1.89131582105612,14.6323914172377,1.61318652228205e-38,3.18227927962173e-36,76.7853041905302

"PTGES",1.78029911397708,1.76644711938897,10.0775796994351,3.00523905607989e-21,3.80672190365599e-20,37.4507123544695

"ITGA3",1.7802758372656,1.94731487129822,14.5911722875432,2.3649842949793e-38,4.48589008259214e-36,76.4064462979987

"SULF2",1.77953700510352,3.75003200846798,12.595085145474,1.76272268139259e-30,7.97537678018451e-29,58.4613648419744

"S100A9",1.7734468387499,4.85091200823183,9.29805061206696,1.30722822137143e-18,1.20576318797945e-17,31.4589719746215

"MRC2",1.7733244318914,2.22075763004767,15.6358243265203,1.348316126409e-42,7.38827299637823e-40,86.0850153389208

"COL6A2",1.77186476690733,5.51912404211172,13.3841462517365,1.51118825351036e-33,1.19561658880673e-31,65.4504981825377

"SLC6A6",1.77077677453041,1.67809144070404,16.3906256999183,1.06603894888647e-45,1.45242706782175e-42,93.1598583053489

"C1QTNF1",1.76921978827825,3.02885411198418,12.1810673812564,6.71315161316257e-29,2.37610234729044e-27,54.8602802235911

"SLC38A1",1.76913930335723,2.85805715909746,12.3441907034987,1.60927303548406e-29,6.24913243044268e-28,56.2732545394262

"CAPG",1.75961815768555,4.36533189973195,11.5701762922755,1.31429788116126e-26,3.38763713445658e-25,49.6414372136014

"LCN2",1.75869888616088,5.64307410920486,5.5220728810429,6.35112057829938e-08,1.4138553860358e-07,7.3614369722058

"DEFB1",1.75709873844936,7.18939821469542,5.37662255224555,1.35320450502853e-07,2.90238629340347e-07,6.63009187994424

"CERCAM",1.75235113485026,1.76145938129312,17.4684615953964,3.6568269421848e-50,1.80342515365414e-46,103.342461682876

"DBN1",1.75131579075192,3.29010714824121,13.3338349004146,2.38126962585748e-33,1.8254344100809e-31,65.0003506753181

"TMEM51",1.74725010617182,2.72103134477477,14.4768793056656,6.82149036932618e-38,1.14686306834296e-35,75.3574100336

"ALOX5",1.74633910907784,1.92709182820021,14.4697941200155,7.28395580167712e-38,1.19740140095348e-35,75.2924508618621

"PRELP",1.74380929208397,2.34963151986955,11.3683368878074,7.31712637681048e-26,1.68362184673268e-24,47.9440515743956

"PHLDA2",1.74105405403409,3.54645404128719,10.9478051141915,2.50007790536403e-24,4.69995585894039e-23,44.4540574942103

"TNFRSF21",1.73749505621821,3.99433289404859,13.0726734871833,2.49956748296279e-32,1.5669958012896e-30,62.6731840480445

"DDR1",1.73278949001573,2.807364929102,11.1225205212945,5.80974535568154e-25,1.18804677818907e-23,45.8961750821787

"KRT7",1.72967585388406,3.00161152845548,6.88431009401758,2.52491287761781e-11,8.77727585158729e-11,14.9811543341277

"LAMB1",1.72782124935176,4.24903011689078,14.2133383819298,7.78000724903678e-37,1.02772506472767e-34,72.9470627041208

"CREB3L1",1.72768803925472,1.54593345833445,13.8226773278232,2.80394614266631e-35,2.98448799861497e-33,69.3976999429254

"SOCS3",1.72763304187084,4.28899972228997,12.3464182195391,1.57810638554044e-29,6.16044432033529e-28,56.2926029215954

"COMP",1.72542181554545,1.06682713878967,14.363074599514,1.95440096214344e-37,2.8629071519715e-35,74.3150542742119

"PLTP",1.72271424632097,4.297465966373,11.6916706938357,4.64522184355563e-27,1.26334663925378e-25,50.6698111917338

"CTSK",1.71807877865563,3.33512640130082,15.2605354250103,4.59107609171313e-41,1.69812426942239e-38,82.5908169400281

"ITPR3",1.7151400760818,1.45925374022253,13.7595223654624,4.99140141946487e-35,5.02365877557706e-33,68.8267308996538

"MMP14",1.71466721019512,5.14264611905109,14.5199132545345,4.57899438456211e-38,7.87746766506935e-36,75.7521406373095

"TIMP2",1.71199223951948,4.87411440038511,12.6976583644106,7.10192067404773e-31,3.3894489152431e-29,59.3609278049205

"SOX9",1.69997535905629,3.75906800801192,9.27513047644496,1.55614390353414e-18,1.42205985502085e-17,31.2872007140687

"LTBP2",1.6987164493992,2.68335189945723,13.7824300450794,4.04965997546584e-35,4.14545183409985e-33,69.0337393521916

"FBLIM1",1.69633619971578,3.52790098248282,14.9186319584667,1.12387912496638e-39,3.02501450379726e-37,79.4236276021288

"PAPLN",1.69532439712648,1.81120401458613,12.2695316541545,3.09703144738388e-29,1.16001469022897e-27,55.6255907720256

"CLDN4",1.69205273420316,2.76496050846557,6.87732895368832,2.63692819556843e-11,9.14947294874178e-11,14.9387285548045

"MUC13",1.68987539024961,4.04811642324442,5.45198437931102,9.16260661446296e-08,2.00178329682486e-07,7.00695192747838

"TM4SF1",1.68952819028408,5.79171498988062,12.9538856697952,7.24329191052803e-32,4.02874074497226e-30,61.6201436082282

"HKDC1",1.68903438255008,3.91241626647213,9.27326388758651,1.57837291211569e-18,1.43793271149949e-17,31.2732233947721

"ANTXR1",1.6806508054624,2.17019870971087,14.8624905828238,1.89700625301107e-39,4.75698432428793e-37,78.9051724108446

"SELENOM",1.67773912331873,4.11422546356374,9.81680326854114,2.36578546671216e-20,2.70318532466951e-19,35.4148183054969

"OSMR",1.67676646580324,2.94409723067512,13.4824266699382,6.20613834991334e-34,5.2770009705154e-32,66.3314868609064

"SLC34A2",1.6735439564884,0.91322477443241,9.96809579511826,7.1727233736203e-21,8.64877280462203e-20,36.592247694142

"CYBA",1.67204195784892,4.66116403497287,10.2364267222305,8.42816271226851e-22,1.14714505361557e-20,38.7055873669731

"HEYL",1.67034539899625,2.82200782782222,13.5816130043458,2.52247397772888e-34,2.31801257767073e-32,67.2227651743237

"CCL2",1.66838795006768,3.48882849681075,11.9571568085484,4.70762770561778e-28,1.48190110435351e-26,52.9337366747276

"IMPDH1",1.66480007397062,2.94813347608097,15.0248982810086,4.16710399989545e-40,1.18562122458564e-37,80.4062484203093

"ASPN",1.66477746511477,3.39059854052478,11.3950513570719,5.83441050635422e-26,1.35510366470189e-24,48.1679012037618

"MXRA8",1.66371907286265,2.87183242915927,13.8109103177601,3.12219924155805e-35,3.29949555563224e-33,69.2912554030141

"IGFBP5",1.6603357132038,4.28502315108258,10.8850817459879,4.21005464590115e-24,7.69935209964246e-23,43.9391350054139

"TMEM119",1.65924600731959,1.57530945129256,13.9377053612193,9.78837234461854e-36,1.09711340029266e-33,70.439712533269

"FBLN1",1.6561433240014,3.23738050274747,9.84988512318004,1.8239999487749e-20,2.11324034785628e-19,35.6713881164199

"ICAM1",1.64754213324162,4.80276718136911,11.9234016879048,6.30586444516816e-28,1.92758810880709e-26,52.644645067662

"GPC4",1.64500437383165,1.58604588378259,12.0937381807993,1.43751875090565e-28,4.82269612690454e-27,54.1070768874927

"STMN2",1.64371921387246,1.10150969248557,12.2586564699745,3.40645124286123e-29,1.26801723780723e-27,55.5313845094132

"SLC16A3",1.6414438399003,2.29298918793937,13.2147759748562,6.96872903595286e-33,4.93312660702979e-31,63.9374319629809

"SFRP4",1.63936831544355,1.59652846529342,13.6290694536674,1.6384256093218e-34,1.55387864678949e-32,67.6499590457682

"OR2I1P",1.6347418585155,6.32235927016444,7.40973185314449,8.7880699020222e-13,3.70004252135511e-12,18.2675399475115

"COL16A1",1.6347380917922,1.53857618220337,16.2088620074311,5.98388068470816e-45,5.87554362846635e-42,91.451100293983

"FSTL3",1.63114562433493,3.82548717175175,10.0192670592151,4.77954443762928e-21,5.87808478426643e-20,36.992819623788

"TSPAN15",1.62553878914722,3.50716983699616,12.6952556756393,7.25500575613484e-31,3.45137653254067e-29,59.3398239956444

"LOXL4",1.62436311049478,3.32680694816145,8.02438677371795,1.39011798287948e-14,7.54467922109386e-14,22.3356125279176

"NIBAN2",1.62294848437166,4.63552384921794,14.1874501161219,9.87488955649998e-37,1.23812704227472e-34,72.710957747814

"COL14A1",1.61806029060948,2.29026321503176,11.3148986986104,1.15010991892391e-25,2.57426267026917e-24,47.4970266135025

"SYT13",1.61616898811179,0.852134217362526,11.5154235044335,2.09679875673446e-26,5.29388013752326e-25,49.1796088093949

"THBS1",1.61414642342427,4.15459619250418,10.3710895409454,2.84460205439481e-22,4.14639284677548e-21,39.7778927610629

"ABCC1",1.60941014450502,2.26191935511607,14.9273702835038,1.03589264191822e-39,2.8917040824868e-37,79.5043665996632

"GOLM1",1.6083572668403,5.1943291864106,10.4812597561003,1.16327844807121e-22,1.7742994473416e-21,40.6608260963199

"TPM4",1.60643184502982,5.22146004503006,16.202531538918,6.35408570837861e-45,5.87554362846635e-42,91.3916409888916

"AKR1B10",1.59556868062599,7.35480628040465,4.20381167849985,3.30046616308826e-05,5.42921913307658e-05,1.36037416338914

"SCRN1",1.5948629291486,1.90128690400437,13.0234716298929,3.88541262691882e-32,2.29022628746071e-30,62.2365909754972

"MMP12",1.58894082478292,1.00413293080298,11.3967206504378,5.75238956562276e-26,1.34026147438408e-24,48.1818970545133

"ELOVL7",1.58685094176379,1.79421519731646,9.84416169641987,1.90801366233996e-20,2.2071197915809e-19,35.6269636575769

"MARVELD1",1.58365351963531,2.45904762867938,15.4057159791292,1.17521457987988e-41,5.1139116792126e-39,83.9404671485886

"NCF2",1.58260654948282,2.90745753111223,13.2395894257507,5.57286961213387e-33,4.0416963682118e-31,64.1586849191513

"ANXA13",1.58150451283413,3.07045935364518,7.73721366513978,9.92502764883333e-14,4.76910633531956e-13,20.4059813879526

"PMP22",1.58140229690013,3.11688356231113,15.796825872968,2.953028245054e-43,1.89956751676409e-40,87.5891827694144

"SPINT2",1.5788425631853,2.60685531824408,7.66326652436428,1.63340908775265e-13,7.61622674229449e-13,19.9172454670771

"DCDC2",1.57473512554306,2.87315381616129,7.66802353939571,1.58204775155565e-13,7.39538593499712e-13,19.9485839431531

"ITIH5",1.57401349365659,1.03791479303782,10.8170945598295,7.39412159387368e-24,1.30700154099595e-22,43.3827026701837

"JCHAIN",1.57172440650218,3.05568787749189,6.95435596736039,1.63049617641323e-11,5.80442515159618e-11,15.4086672880034

"JAG1",1.57094200562897,3.1399104028861,14.6434648577369,1.45555984941386e-38,2.95000109206548e-36,76.8871306215251

"FNDC1",1.56944252462735,1.06985326757064,15.3154489915894,2.74290249076968e-41,1.04054467566506e-38,83.1009925451866

"CXCR4",1.56878875913136,4.55683531749685,12.9975747368395,4.89965897541308e-32,2.82064025452282e-30,62.0070348453705

"KCTD17",1.56710513270041,2.98988912238743,10.963394167621,2.19584585092174e-24,4.13854004641875e-23,44.5822664987858

"PLPP2",1.56660534404472,1.99032121870819,7.69809019109005,1.29235735613413e-13,6.10291320906623e-13,20.1469829737724

"MFAP2",1.56654297647027,1.25386464853573,14.2011868703721,8.70146295282154e-37,1.12928196830697e-34,72.8362237542869

"DAB2",1.56519384369015,3.55735000484316,13.5533569280119,3.2606721747967e-34,2.95961011203173e-32,66.9686401114348

"MMP11",1.56056688780216,2.57883483477277,10.6449731662325,3.05265242098347e-23,5.00154956461246e-22,41.9820615305143

"TNC",1.54938618695977,2.0949690619125,12.6242874457744,1.3611552782014e-30,6.21552232746594e-29,58.7171785331742

"CHST11",1.54725707490593,2.04769424879973,14.3878095566164,1.55503371770695e-37,2.32391150035094e-35,74.5414154271829

"SLC6A8",1.54713683496559,2.78644465064673,8.67021128552547,1.40711753098131e-16,9.85715145400973e-16,26.8512348011019

"PLAUR",1.54627514895709,1.90836780252867,16.9502357047971,5.17422527621416e-48,1.27587771602647e-44,98.4373221325592

"ADAMTS2",1.54564636510709,2.19122858165119,13.0331930030711,3.56128206133231e-32,2.13316470030006e-30,62.3228063952453

"NCK2",1.54446870882686,3.76127596927539,11.3954415697191,5.81513372930697e-26,1.35275005542605e-24,48.1711727753425

"LAMC2",1.54275829719482,0.926812667474499,11.8408521179948,1.28687973061264e-27,3.74791055401852e-26,51.9391731020548

"ITGAV",1.54146347369891,4.11479237278786,14.0075795595671,5.15842038203363e-36,6.00935665765257e-34,71.07396434656

"TMC5",1.54137641324802,1.23689268921784,10.2413465673501,8.1013468321872e-22,1.1087828527494e-20,38.7446278050794

"IL2RG",1.53698940101583,3.51028330038375,10.4430637836895,1.58695371012554e-22,2.3836528062241e-21,40.3541432949542

"LTB",1.53597902582692,3.42453470713483,9.73702882223901,4.42040608831028e-20,4.89520270034061e-19,34.7981903948203

"LOXL1",1.53484768413932,1.75982408837435,12.9041273974728,1.12989611388518e-31,6.07884109270227e-30,61.1800910214949

"SMOX",1.5347623462092,3.03647445687428,13.1417309323431,1.3444774165039e-32,8.96015467440324e-31,63.2869630037785

"ARL4C",1.5323620522298,3.4232385031124,12.7689565496332,3.76906894111305e-31,1.87755471325817e-29,59.9878659577759

"LGALS3",1.53052527924005,5.23209743499524,9.48461478960405,3.13380829288609e-19,3.12852184164978e-18,32.8667842763373

"S100P",1.52660271682484,3.58423803900788,4.65871702665398,4.45660279123358e-06,8.01256997160054e-06,3.26879376296067

"TUBB6",1.52374102271503,3.03293490465218,16.2461530914873,4.20124660489463e-45,4.78134180918585e-42,91.801433469435

"PODN",1.52373473104652,1.84552530521541,11.219143559461,2.57974269501288e-25,5.51550479374502e-24,46.6985443914927

"ALOX5AP",1.52229649623573,2.18652139380225,13.2095027788727,7.30760735606225e-33,5.12398345179815e-31,63.8904314278091

"SRPX2",1.5187081840071,1.93092852697446,13.2172419880219,6.81567527195114e-33,4.84797671387101e-31,63.9594140475665

"RAB31",1.51727934075638,2.56538857292285,16.9208408799256,6.84909536264348e-48,1.44760522700443e-44,98.1595583689441

"KRT80",1.51448089117739,1.03573634987969,11.4112522660715,5.08516208835623e-26,1.19610450075088e-24,48.3037754282727

"TUSC3",1.51440128751228,1.91307579686793,10.1463877370978,1.73492508245796e-21,2.2515979469268e-20,37.992942413603

"ITGB4",1.51348041403666,2.43089605996906,11.7002759531238,4.31453982214404e-27,1.18210401238187e-25,50.7428349683058

"S100A4",1.51236501356469,5.07698544386002,11.1142820996695,6.2251783808726e-25,1.26513068880508e-23,45.827919885229

"PNMA1",1.51040021281499,3.51072795902579,16.0540034638807,2.59566415994452e-44,2.13349173590995e-41,89.9976642014387

"MYOF",1.51004964630047,2.08165581524562,14.8909110164142,1.45547035445282e-39,3.77783927967184e-37,79.1675709939427

"CDR2L",1.50985975629876,1.98483641830831,15.660212358676,1.07144784801695e-42,6.09695035054263e-40,86.3126737127868

"PI3",1.50921393052854,2.03578431115508,7.11042950578313,6.08287709283855e-12,2.2928959640394e-11,16.3731037703344

"TACSTD2",1.50881389381468,1.48074391126908,9.91000530902863,1.13556528200527e-20,1.33976781078691e-19,36.1389338626978

"HOMER3",1.5076713021735,2.96546984989069,14.2281521855881,6.78736855667159e-37,9.04676736900507e-35,73.0822219941859

"ENO2",1.50378246443458,1.54552780502045,13.3169941058226,2.77241900685619e-33,2.09275200032844e-31,64.8498016833808

"DPYSL3",1.5036813737902,2.30489511876684,14.3399349304544,2.42015343836019e-37,3.51040883534696e-35,74.1033893165321

"TREM2",1.50280067695607,3.13318452102261,10.4959066627137,1.03250741021782e-22,1.58464181889757e-21,40.7785883038443

"PLBD1",1.50138060180831,2.37662852623514,10.4440645980686,1.574103904223e-22,2.3691624886042e-21,40.3621713376804

"OLFML3",1.50083842375528,2.79372150858265,10.8518846436541,5.54392011182471e-24,9.95416238524837e-23,43.6672150103557

"ADAM9",1.49937468902425,3.39683323279446,13.0884890577587,2.16885788295805e-32,1.38311432665364e-30,62.8136497193796

"OLFML2B",1.49913730219573,2.64336662439253,14.3943641209952,1.46360982169039e-37,2.20960278692953e-35,74.6014171679413

"PTP4A3",1.49809691234687,4.0503027129736,10.4870010850698,1.11016418637918e-22,1.69678503486363e-21,40.7069763015988

"TEAD2",1.49418303589039,3.81160093314842,12.0434527251095,2.2262713536514e-28,7.23905157742251e-27,53.6744186571625

"RAP1GAP",1.49129380881939,3.93991471852173,10.175888837024,1.36997449728297e-21,1.80166868331569e-20,38.2260531626438

"COL4A2",1.4871400803878,5.799379679843,12.9085806578819,1.08584352112067e-31,5.8846354926668e-30,61.2194492476631

"MDFI",1.48668404144227,1.49942267468318,12.1408370109458,9.53647929793246e-29,3.29654699095586e-27,54.5130134688395

"STK39",1.48626468869985,2.55469681286391,11.3723089473281,7.07497726088923e-26,1.63044063200711e-24,47.9773190144696

"GLIS2",1.48605624796219,2.50892066358458,11.504322011461,2.30481081690762e-26,5.76982674046503e-25,49.0860942185413

"NRSN2",1.48052244150638,2.94757601573922,9.31920686823918,1.11270411421064e-18,1.03667867567673e-17,31.6177559757031

"CPXM1",1.47959406375032,1.69555701787111,14.3670191349497,1.88445318856593e-37,2.78804849248329e-35,74.3511454017263

"ANXA5",1.47690556310452,6.90740803179761,13.6685604522884,1.14372925562285e-34,1.10597871483269e-32,68.0058161800939

"TMSB10",1.47396265874349,10.6835005360562,10.5819512796114,5.11549211701905e-23,8.19087725879836e-22,41.4721633309972

"SERPINE1",1.47241124363606,5.8239578611326,7.86614312155763,4.1312777116978e-14,2.09251125452136e-13,21.2661400439261

"EGLN3",1.46933475449857,1.55257672411088,11.7983443857098,1.85651284273299e-27,5.30253040699509e-26,51.5767474415668

"DSG2",1.46809531113553,3.43961822516491,9.02832477588792,1.00002406350234e-17,8.27480761717962e-17,29.4543476533997

"BASP1",1.46523589558975,2.55362702940993,10.478074947073,1.19382305979375e-22,1.81527360428043e-21,40.6352316682996

"DUSP5",1.4647079774091,3.68218903050742,11.9280851214148,6.05537238757867e-28,1.8586978106686e-26,52.6847344209544

"HIF1A",1.46282106561235,4.72334877240433,13.3751157509473,1.63977712926677e-33,1.29045226742031e-31,65.3696576585927

"F2R",1.46056498860788,3.45279419589271,12.7942504191879,3.00944199596536e-31,1.54599633091346e-29,60.2106011775898

"CLEC11A",1.46055814998854,2.94777550000281,12.4023306036164,9.65480703182129e-30,3.8921762952533e-28,56.7787283528216

"MOXD1",1.45877197391147,1.23258855876922,13.7651045084294,4.74350860090853e-35,4.8068636815371e-33,68.877164758584

"PTGIS",1.4583893778685,1.34520192329376,13.0173001283344,4.10627806832751e-32,2.4012800008263e-30,62.18187024175

"GPR183",1.45632875733716,2.31669381148023,12.8592437955818,1.68651779341312e-31,8.97555063077234e-30,60.7836891110836

"FBLN2",1.45542705957876,2.75396200555809,8.96008869148185,1.66357114517676e-17,1.3271064615117e-16,28.9531193996189

"ADGRA2",1.45365323184423,2.12259509006531,13.5992856034177,2.14814728376702e-34,1.99885780272535e-32,67.3817939802318

"TGFB3",1.45319439697198,2.36370058364671,15.1540190492709,1.24548558030373e-40,4.09487981346528e-38,81.602368637254

"VTCN1",1.45235022668202,0.78157630689182,9.78292415050814,3.08621011114015e-20,3.47492226745194e-19,35.1525858703073

"FGFR2",1.45157839333018,3.31725612641607,6.46277453547616,3.27379337886197e-10,9.83667202279912e-10,12.4797479377604

"PDGFRA",1.45130778748745,1.42018807392119,11.4750196832822,2.95784240389308e-26,7.24524476251625e-25,48.8394653481152

"MAPK13",1.45107351010756,2.12111673198268,8.90464236889246,2.5111932855269e-17,1.94112354542166e-16,28.5476289254397

"FSCN1",1.4474070505679,4.16849027750366,13.6447634883673,1.42038826992413e-34,1.36458730217711e-32,67.7913398067069

"APOA4",1.4412145504817,3.95157803945756,3.7882329476406,0.000177239331405759,0.000270475080778567,-0.226982908912793

"ELF4",1.44026160776733,1.8388779605201,14.6183526667888,1.83774224859544e-38,3.57755217999599e-36,76.656238614369

"UNC5B",1.44001891586826,2.36011920947899,14.1094803740285,2.02341660779896e-36,2.45380727150702e-34,72.0006103155761

"F3",1.43914610668833,1.50098545497076,14.7409766787463,5.88084346698401e-39,1.33857044760044e-36,77.7846521724879

"NCEH1",1.43835769907709,2.24747765564879,12.1157956957578,1.18626602129624e-28,4.04396446660782e-27,54.2971033290506

"PLAU",1.43604347882731,2.7889087941932,15.4441127881741,8.19229774708959e-42,3.79924013648184e-39,84.2978665814743

"CRISPLD2",1.43518885119307,2.15643269253669,11.9485320479381,5.07284651619402e-28,1.57014151060859e-26,52.8598370409974

"RRAD",1.4348160221541,1.83024133122623,11.8830633991944,8.93804707298617e-28,2.66073252404085e-26,52.2996426132934

"TMEM156",1.43447742927621,1.65879909264418,9.67645941476699,7.09165427063526e-20,7.61400761495273e-19,34.3319841316739

"GPNMB",1.43377875303199,3.88860699160685,10.8127420033086,7.66513506467961e-24,1.34846222689578e-22,43.3471405628917

"LDOC1",1.43176650403979,2.44805070614863,9.04823721438712,8.61620087314006e-18,7.20614425766575e-17,29.6010696402994

"CD44",1.43117244503566,3.15951991213789,9.6532596138717,8.49538898135216e-20,9.02292031436505e-19,34.1538689973956

"ETV4",1.4309226978294,2.94516049714891,7.12034361979126,5.71052370476325e-12,2.16356461490326e-11,16.434915317957

"G6PD",1.42874454839571,4.20280911449208,9.32859832247915,1.03582943863448e-18,9.72404603083575e-18,31.6883127758913

"GSTP1",1.4247152395302,4.79506026981041,10.2381077831085,8.31504595455547e-22,1.13383506818109e-20,38.7189259197876

"KIRREL1",1.42399320690949,1.8508756598508,15.1139902831757,1.8115313380367e-40,5.70246939281978e-38,81.2313098890162

"ADGRE5",1.41581089436509,3.73881132214918,12.1186663746784,1.15696239738924e-28,3.96232839568837e-27,54.321845176241

"CRYAB",1.41424811371935,2.66380625086441,8.45538970814851,6.65263190704887e-16,4.29994272891166e-15,25.3229855865771

"MISP",1.41265337231936,1.28625531296804,7.17720101991627,3.97023015469836e-12,1.53487209664913e-11,16.7906591328115

"NOTCH3",1.41159926094428,3.42010417651081,12.3563686612755,1.44606570425225e-29,5.69003779106702e-28,56.3790506807507

"FGFR1",1.41037757174099,1.58649539723979,14.1049332605882,2.10979190802947e-36,2.53775376254439e-34,71.9592186336196

"GAL3ST1",1.40937999504865,2.66359773315229,6.71786320143971,7.04497193740989e-11,2.31571561461851e-10,13.9787056315165

"SCTR",1.4082853474812,1.12375673224113,9.69107137197672,6.32835595035052e-20,6.87430442624346e-19,34.4442962795477

"ISYNA1",1.40691949985377,3.47611112601894,9.15905882789495,3.74691910997346e-18,3.25708979036765e-17,30.4213637993708

"ATP1B3",1.40608126199275,4.59030999329549,13.1235464816049,1.58313442503294e-32,1.04099883637166e-30,63.1252284877162

"METRNL",1.40351509575097,3.18402440458415,11.207222065964,2.85204817964745e-25,6.06265126693737e-24,46.5993636688425

"TGFA",1.4034911110603,1.38651026920326,11.2751378961731,1.60912312560993e-25,3.52695950272576e-24,47.1650720825492

"GLS",1.4031230240933,3.10332666610413,12.2314858221572,4.32078388757346e-29,1.56298282681294e-27,55.2961705530222

"LXN",1.40236074138553,2.29419360881834,14.6805604753884,1.03128067093138e-38,2.24379375388672e-36,77.2283890938993

"LGALS9",1.4009508160951,3.45558697970706,13.2927414707534,3.45087432635612e-33,2.54008386360392e-31,64.6331092622343

"TMC6",1.40083107309366,2.38051954150147,11.8150829751904,1.60714126034128e-27,4.61702037800956e-26,51.7193932824798

"HLA-DQA2",1.40058204537448,3.30103902503866,7.33683084940253,1.41519846685926e-12,5.8128432307559e-12,17.8007506077361

"SFXN3",1.39830470968837,2.85855593677014,14.8590834194063,1.95821225140223e-39,4.82862504324932e-37,78.8737230950128

"FMNL2",1.39690459159186,2.18809662011223,12.0250116942611,2.6131234990623e-28,8.38636923397543e-27,53.515945149182

"ASNS",1.39573522868402,2.17605976979061,10.1467101613488,1.73045618722157e-21,2.24776991132072e-20,37.9954880907277

"COL5A2",1.39573005671832,3.74982332839343,11.2687838237256,1.69775423343636e-25,3.71023247912717e-24,47.1120754227534

"SEMA6A",1.39569004390996,1.70087549917543,10.9006731362703,3.69902271903671e-24,6.82382058954466e-23,44.0669911562633

"HK2",1.39374558674115,1.59696342576051,9.75680005011666,3.78699131824059e-20,4.22856879648072e-19,34.9507406384675

"TNFRSF11B",1.39186072893588,2.19255164302344,8.99453326327854,1.28705541826614e-17,1.0433964336026e-16,29.2058294560215

"EMP3",1.391702125237,3.86533764796103,13.1259095105001,1.54988003791256e-32,1.02368192682662e-30,63.1462410178943

"SGPP2",1.39160804210732,0.955616543887785,11.1007796285862,6.97090062774904e-25,1.4012836248308e-23,45.716106079549

"FXYD2",1.39072046915376,1.56623236249103,6.5082392873978,2.49779715616135e-10,7.63058206182267e-10,12.7435542957133

"C15orf48",1.38941197116444,2.55294955863791,7.29014803051354,1.91672302707429e-12,7.73960621876751e-12,17.5036387430832

"LYZ",1.38926374061308,7.04601880921427,5.34097796220073,1.62482678181151e-07,3.45442049675259e-07,6.45340370981712

"CYBB",1.38924317278565,2.87229596257545,10.7589049265091,1.19571336403992e-23,2.0546549617852e-22,42.907880137684

"ECM1",1.38863202251329,2.42119999153462,12.9322268851411,8.79068641636554e-32,4.83487752900105e-30,61.4285205452187

"FIBIN",1.38795983356528,1.48688163404985,11.421301878296,4.66936015902222e-26,1.10356523247179e-24,48.388105658128

"FSTL1",1.38545496642092,3.73860457441089,11.4605219557704,3.34603807919388e-26,8.07579663648833e-25,48.7175512646647

"PRSS23",1.38532188504197,2.52082810508143,14.0026810526331,5.39551347980312e-36,6.23645483856931e-34,71.0294693701593

"GPX8",1.38526454781829,1.70698150796912,15.9924575810861,4.64768630659905e-44,3.61907994242805e-41,89.4206663370946

"NFE2L3",1.38468610834419,2.18919379464827,13.5122650846487,4.73473306663182e-34,4.09651320004782e-32,66.5993851085387

"IQGAP1",1.38382734893531,3.02023201329294,14.8048787141311,3.24458194020222e-39,7.7425144847245e-37,78.3736314712014

"LTBP4",1.38262445129767,3.16764230410751,10.7495295268515,1.2918317033811e-23,2.20700347015282e-22,42.8315014211727

"SLC7A7",1.38225233338855,2.23367539049788,14.5232436104658,4.43984439909945e-38,7.72794092760898e-36,75.7827014251957

"CTSC",1.37995512981063,3.83403552180026,11.8870477095307,8.63552283388653e-28,2.58106182479497e-26,52.3336964599187

"PAQR5",1.3796434667933,1.87475232580889,8.84464482349183,3.91400012248196e-17,2.94374229536812e-16,28.1106788532554

"IGLL5",1.37894970487159,1.86458291562467,6.76785823092464,5.18647079311177e-11,1.7360596240744e-10,14.2778072997169

"LPAR2",1.37823235722886,1.82217243659734,12.0690752813393,1.78165886693294e-28,5.8707445292367e-27,53.894779840828

"CD248",1.37773167646214,3.45112309805226,14.1906050686695,9.59213715733585e-37,1.22341094174814e-34,72.7397248186257

"COL4A1",1.37508658790815,5.73963839479731,11.4814560550661,2.80019704622372e-26,6.89333033259232e-25,48.8936131398226

"COL9A2",1.37444432527585,1.31273616640041,11.7298106017686,3.34794958560079e-27,9.29322966584683e-26,50.9936492850495

"LYPD1",1.37289917113921,1.97111393034824,8.13455799552664,6.45627878111574e-15,3.66260140209384e-14,23.0889863701801

"AGRN",1.37238719406972,5.04256618787847,13.0404998353984,3.33553254281579e-32,2.03083143913414e-30,62.3876234664569

"SLC12A2",1.37147612769233,2.23530002138719,11.9120556250783,6.95631388344732e-28,2.10898901445908e-26,52.5475533924011

"LBH",1.37075531480606,3.59338384380912,12.9753402559746,5.97849534264362e-32,3.41512890325917e-30,61.8100763496304

"BHLHE41",1.37011807788177,1.26365003765501,12.4420010140244,6.80937535936443e-30,2.83787911103653e-28,57.124179109283

"TUBA1A",1.36873818559266,4.05239046559976,14.0591121178622,3.21429671750443e-36,3.77424761392683e-34,71.5423337819879

"MYL9",1.368180095484,5.37299046048335,10.9883600634202,1.78349796171703e-24,3.40475514111012e-23,44.7877845843806

"CCN2",1.36796222472492,5.78689120348858,9.58740195273113,1.41660510875372e-19,1.47492417902965e-18,33.6496347563953

"LAPTM5",1.36777655979321,5.62614980338409,12.193419965355,6.02659521215722e-29,2.14335279240063e-27,54.9670041600536

"SLC2A6",1.36753318719609,2.64106481231241,10.9427469127372,2.60753160965704e-24,4.88953487514271e-23,44.4124771142743

"PITX1",1.36721829406119,1.42593396106572,8.37922759114178,1.146984083808e-15,7.18747544258341e-15,24.7873394411323

"TYRO3",1.36644366006199,1.40373244229623,13.211864187411,7.1538678182517e-33,5.04007020814447e-31,63.9114780901484

"KCNE4",1.36482716270272,1.49258616876986,13.9828171168774,6.47368756291289e-36,7.42466724754235e-34,70.8490854542621

"AQP1",1.36298080831613,5.03711969571379,8.76297653482787,7.1394894350977e-17,5.1829610496698e-16,27.5189812635503

"HSPG2",1.3620401377024,3.26175424962912,9.4390863423065,4.44747409564446e-19,4.34900061104162e-18,32.5216565166128

"NXPH4",1.36129767012303,1.71027720543633,7.84080184446135,4.91180383554631e-14,2.45756299448453e-13,21.0962745888914

"ARL14",1.36038930958798,0.989166549965715,9.8086830608204,2.52154783409998e-20,2.87192457317238e-19,35.3519178569939

"GCNT3",1.35984758186601,1.28132578151767,10.1907075101187,1.21655192533898e-21,1.6156988990476e-20,38.3432891351342

"WNK2",1.35972642704707,1.08369713492914,9.32411959773981,1.07181020325867e-18,1.00265972225546e-17,31.6546592380947

"MFGE8",1.35776924082084,3.16631887300959,14.6065407607983,2.05067064267296e-38,3.94021716342162e-36,76.5476703822043

"UAP1L1",1.35763466550904,1.78141671946088,11.1762015522537,3.70214006366019e-25,7.72541075343478e-24,46.3415297613391

"EPS8L3",1.35587612315326,2.8214477772706,6.73773127283505,6.23892006110181e-11,2.0635998726582e-10,14.0973621903199

"RHOV",1.35487635432844,0.781126454133556,10.6302911551819,3.44327641974639e-23,5.59201697367156e-22,41.8631296305108

"SNAI1",1.35377648274623,2.04586606741573,13.3425189257268,2.20160034675618e-33,1.70537576598208e-31,65.0780073657235

"UBD",1.35311079355847,6.66861808629396,6.61603740195333,1.30770956419014e-10,4.15451213274493e-10,13.3748822120965

"TOR4A",1.3523514342236,2.11395560551256,13.1663425350229,1.07758840837716e-32,7.31326628529359e-31,63.5059879783346

"ENDOD1",1.35133806794124,2.50985967012519,12.8261341871958,2.26552564772103e-31,1.1895690052328e-29,60.4916039800828

"TGFB2",1.3510268861133,1.2064814182998,13.4948004262545,5.54746593887072e-34,4.74420569743308e-32,66.4425583119191

"GGT5",1.35040296399142,2.89815864976486,9.94577922288909,8.55880955644607e-21,1.02449504358916e-19,36.4179189102028

"LGALS3BP",1.34984288731588,7.447516222712,7.12898672671403,5.40427341538918e-12,2.05279140386862e-11,16.4888556275805

"KCTD12",1.3493834362519,2.99113509788359,13.160287731952,1.13789142089898e-32,7.65231980554562e-31,63.452091258525

"YBX3",1.34918946109532,2.88761459294628,11.5096434161621,2.20265710364695e-26,5.54222990620009e-25,49.1309143836519

"MFSD10",1.34216638691114,4.11299242374134,13.2036209218786,7.70501954529489e-33,5.35191381092197e-31,63.8380136026061

"TEAD4",1.34211610809434,2.46229462355471,12.7291269823663,5.37042718629384e-31,2.63993986825505e-29,59.637471433634

"TPPP3",1.34020682312514,2.3584428929503,12.8872699296487,1.3134026001724e-31,7.015087173123e-30,61.0311492757166

"PIP4P2",1.33969764046298,2.28024251662602,12.9591139694368,6.91242330139491e-32,3.88856664426379e-30,61.6664179530227

"BIRC3",1.3388618989478,3.71268770141027,8.36750410575884,1.24694514177826e-15,7.78091664808494e-15,24.7051794705862

"LIMK1",1.33876349059526,2.68041220201989,15.0592757188309,3.02197328695718e-40,8.7666852510846e-38,80.7244775283267

"F2RL1",1.33875355740868,3.08910207446657,7.44325793418382,7.05094160295584e-13,3.01237889158913e-12,18.4833535373921

"COTL1",1.33789497692253,3.64760872411786,11.9353440271836,5.68657441828324e-28,1.74912408562371e-26,52.7468829637126

"GLIPR2",1.33782931978957,2.60860996444542,15.080238406956,2.48407062717514e-40,7.35036498581125e-38,80.9186096073961

"PDLIM7",1.33470970105306,3.53994956408173,16.4306711151012,7.28688351369777e-46,1.19788268427954e-42,93.5367092897268

"TC2N",1.33437820627158,1.83977394951004,9.5987431927644,1.29738787528659e-19,1.3546121111408e-18,33.7363216069515

"STC2",1.33433569046922,1.95484750372804,12.4042764983479,9.490958543356e-30,3.8365773674577e-28,56.7956628380285

"PPP1R18",1.33425839984884,4.25760049891669,13.5498787159799,3.36530766474945e-34,3.02473792334111e-32,66.9373702980609

"LAMP5",1.33408878462972,0.742083079326213,11.3737127769074,6.99131676050102e-26,1.61367443793467e-24,47.9890779183297

"HLA-DQB2",1.33380705792378,2.46741855013278,8.99513696010722,1.28127295544945e-17,1.03927814560716e-16,29.2102641179614

"LHFPL2",1.33162309603537,2.095268126743,14.1918385734301,9.48379803426695e-37,1.22011123406069e-34,72.7509724961477

"COL6A1",1.33026712550084,5.57960219622238,9.83168122950785,2.10476885375537e-20,2.4252379432485e-19,35.5301439039188

"RAB34",1.32937632004667,3.16983758581065,8.02695404017385,1.36560467137654e-14,7.41707823532153e-14,22.3530864497906

"MUC1",1.32920867163133,1.08600147248949,9.93383220489993,9.40698654745957e-21,1.12148562425193e-19,36.324685206845

"THY1",1.32835046444526,4.21358593675388,12.9727423777635,6.1190845016622e-32,3.4819944308497e-30,61.7870717642953

"RCAN2",1.32829019956559,2.19004759948309,11.4287939148596,4.38155785091128e-26,1.0422049582674e-24,48.4509970711263

"SPARC",1.32723137469465,7.49201861873696,12.2739678607822,2.97900456863055e-29,1.11863889829668e-27,55.6640293437005

"S100A14",1.32639127832953,4.09451729678148,5.36887006423437,1.40824334148905e-07,3.01344521801134e-07,6.59157785075592

"LPCAT1",1.32414096599646,3.6760685149097,11.2919371494618,1.39635524773794e-25,3.08344416272878e-24,47.3052569468522

"FZD1",1.32395119223177,1.70242230383034,13.4812079143337,6.27509087232236e-34,5.30514111177196e-32,66.3205486890428

"TMSB4X",1.32393080452725,9.31999176992003,13.5045116217503,5.07975264652267e-34,4.36947327937808e-32,66.5297533082427

"AKR1B1",1.32363834901386,3.53557849542779,9.52707659489736,2.25884674645991e-19,2.29372941756172e-18,33.1895685541323

"ANKRD1",1.32338323215627,1.45364770878393,8.78683256643054,5.99198001966043e-17,4.40613043692227e-16,27.6914526378853

"FLNC",1.32263573333933,1.51543595849701,7.63089512068964,2.02935518584762e-13,9.36795943045727e-13,19.704361325409

"PFN2",1.32168867217834,2.70449161855865,8.38068870965091,1.13509493879689e-15,7.12504438672042e-15,24.7975846410761

"NPTX2",1.32078290427748,1.46328362165612,6.36269973492592,5.90857874212101e-10,1.71153317767578e-09,11.9042606060952

"MXRA5",1.31994608672549,1.24143443153294,13.5538663442936,3.24562221585353e-34,2.95961011203173e-32,66.9732200855158

"LAPTM4B",1.31956315339226,6.05280025792209,8.59811155065773,2.37669654621818e-16,1.6249179945147e-15,26.3354761353123

"CLCF1",1.31848462058036,2.36283960942713,13.0891311206986,2.15639542104699e-32,1.38311432665364e-30,62.8193534774042

"TAX1BP3",1.3176558698562,4.29650468619059,12.5554868012237,2.50190557533677e-30,1.09513884577241e-28,58.1148529821948

"PRNP",1.31576338988503,4.91440573921497,10.4614424192548,1.36677289922464e-22,2.06551634770465e-21,40.5016337437101

"RAC2",1.31511635797038,3.63786334292658,10.1958074618741,1.16780144221192e-21,1.55514152452974e-20,38.3836586799695

"IKBKE",1.31364787898747,2.2226509640232,12.560116858835,2.40158307862579e-30,1.06063945218712e-28,58.1553468718726

"LAMA5",1.31103877575566,3.36224451183372,11.8753986233552,9.55001748681196e-28,2.83151320074916e-26,52.2341460274981

"SRPX",1.31063017699244,1.68656737629641,9.57150038430671,1.60226949297341e-19,1.66121773991182e-18,33.5281940850756

"KRT17",1.30909143900947,1.33097244617806,9.09524665561969,6.05673497160438e-18,5.17973375172756e-17,29.948262496608

"CDH11",1.30674672116113,1.02529262142076,15.8109664567058,2.58394036032708e-43,1.73769989231996e-40,87.7214294900534

"CA12",1.3065972306174,1.93473574918612,7.06092978110618,8.33038390072969e-12,3.08120074528239e-11,16.0654641288106

"ID4",1.3065342978577,1.76163038889018,11.9217910048213,6.39437458517465e-28,1.95061385541565e-26,52.6308595013103

"PYCR1",1.306383298276,3.17053359393144,6.04439615271064,3.68787891850572e-09,9.64507134511085e-09,10.1220452504301

"IL4I1",1.30625619579,1.7929743530755,10.3242132041725,4.15525506734797e-22,5.91693924171446e-21,39.4037504510955

"IGFBP6",1.30609487514012,2.51461158891007,12.3925237581054,1.05244576880339e-29,4.23123237756688e-28,56.693398982104

"CTNND2",1.30539984255319,1.22042907863366,7.60189305713013,2.4636237395033e-13,1.1208275899739e-12,19.5141898170847

"GFPT2",1.30519532435936,0.879479765955832,16.2258381040638,5.09405934739822e-45,5.38332914605405e-42,91.610567456976

"PLP2",1.30461235597696,5.67133901039637,8.49489009185659,5.00906739882562e-16,3.2893542905293e-15,25.6020736861767

"ADGRG1",1.30346490917213,2.60993658641391,10.4414350252385,1.60808915390791e-22,2.41294919189326e-21,40.3410790790337

"SLC7A1",1.30301572566781,1.71850134246917,11.383068322053,6.45837383527985e-26,1.49299438895258e-24,48.0674604237589

"CD53",1.30079245331929,3.85827474489745,10.5416683046294,7.10952719107032e-23,1.11307359568133e-21,41.1470813209065

"IER5L",1.29972131889825,2.8853040355882,11.702568770391,4.23045755390988e-27,1.16121743061404e-25,50.7622958024097

"TCEAL9",1.29829380533129,5.20958928102922,8.28877868013958,2.18111730809028e-15,1.32524150197929e-14,24.1554857905841

"TLCD3A",1.29797163177111,2.2005054094348,14.6545950673134,1.31261692324838e-38,2.69724546936942e-36,76.9894989993135

"VIM",1.29623788650246,6.68622067307087,14.508202219398,5.10378632054347e-38,8.67936995545295e-36,75.6446898547884

"CMTM3",1.29621555106702,3.44907110537273,12.9206432027505,9.74930534210215e-32,5.32254511204433e-30,61.3260841564705

"EPHB6",1.2936482091853,1.4661796219922,10.0385108772024,4.10164348731084e-21,5.07814354767899e-20,37.1437629937219

"LOX",1.29320129665169,1.91257097266984,11.0787244371239,8.3847167399716e-25,1.67185827719515e-23,45.5336118387016

"GPRC5B",1.29284564271914,2.468079126646,10.7540092394876,1.24498258925473e-23,2.1294239778062e-22,42.8679920661133

"KDELR3",1.29176920537508,4.49370454374192,8.79419751970294,5.67611402805375e-17,4.18217664567008e-16,27.7447604862589

"MSRB3",1.29139170716438,1.92098963520898,14.2625574967117,4.94264592296266e-37,6.64785876638477e-35,73.3962854918676

"RGS10",1.29065691373149,3.511884726046,11.7864002532759,2.05766652872982e-27,5.84322001776539e-26,51.4750150354671

"CHD3",1.28802764169911,2.8231524874403,11.4400857801724,3.98080698281312e-26,9.5301034483366e-25,48.5458226314729

"LHFPL6",1.28750702265755,3.60268705193987,11.9191118684673,6.54434698380231e-28,1.99225542439002e-26,52.6079310294414

"LAD1",1.28732172821377,4.89729072055842,5.95606075954594,6.05223858784866e-09,1.53589828314273e-08,9.64067116841129

"FXYD5",1.28578396695371,4.34151877641878,12.0285201821832,2.53469168703611e-28,8.1701009825053e-27,53.5460873850748

"ABR",1.2831337007257,1.85706231300482,14.900657246925,1.32902110247096e-39,3.5112262876889e-37,79.2575832823386

"APOBEC3C",1.28288350395754,2.72804555407857,11.1619704416704,4.17233264124385e-25,8.62146109318474e-24,46.2233615835758

"SYK",1.28221817271563,1.98288680559449,13.1210046449545,1.61969968179317e-32,1.06032994655442e-30,63.1026274693469

"TAGLN",1.28166121481626,4.93916131341271,10.5491432625963,6.68860328312286e-23,1.05386459610014e-21,41.2073541615776

"P3H3",1.28144583646564,1.52011115702502,12.587698800583,1.88177206640175e-30,8.41112317897703e-29,58.3966968584164

"PROCR",1.2766839503051,3.03051918384682,11.7690088056679,2.38996931577715e-27,6.72235665911082e-26,51.3269683612057

"LIMCH1",1.27642565051684,1.67405834543372,13.0393884489244,3.36892637488387e-32,2.03563267106772e-30,62.3777637876903

"IFI16",1.27619927257274,3.27177503007849,11.4664936236584,3.18034900508751e-26,7.726315850619e-25,48.767759374466

"FBN1",1.27607546833491,2.07649586893556,11.9051814593434,7.38246530723513e-28,2.22451271324936e-26,52.4887488086395

"PLEKHB1",1.2758848698353,1.07855316151812,8.97779875767556,1.45805548511031e-17,1.17111459838258e-16,29.0829758973198

"ST6GALNAC4",1.27530055264009,2.83716539764903,13.44442529115,8.75741818949006e-34,7.15834265820472e-32,65.9905847713702

"CRABP2",1.2749352302373,1.40761826726289,10.8256639418136,6.88808432401567e-24,1.22339985082607e-22,43.4527393767219

"C7",1.27323301659295,3.51145523151828,5.07340164021196,6.21811727904143e-07,1.22515708008281e-06,5.15935587235309

"SCD5",1.27272520096094,1.19764093012178,10.5534242664938,6.45878970861951e-23,1.02091659977592e-21,41.2418834184564

"RENBP",1.27188694905511,3.21299806428843,8.35524211175029,1.36071991558896e-15,8.43850056265895e-15,24.6193289871416

"C2CD4A",1.27124589051971,1.43705221726448,8.64600212141266,1.6784250839975e-16,1.16583563933066e-15,26.6777398283917

"PTK7",1.27120963925748,1.69867023022581,9.24947235105005,1.89085465070945e-18,1.7016541701488e-17,31.0952210159421

"HSPB6",1.2701841328715,3.28261853479606,7.2995252166938,1.80361718694628e-12,7.31483450681751e-12,17.5632063162442

"C4orf48",1.26988287119452,2.45114495084714,8.96376583034051,1.6186640189456e-17,1.29309579699245e-16,28.9800680206755

"SPI1",1.26942228690724,3.64354353806728,12.0404355589058,2.28541756984936e-28,7.41507740042132e-27,53.6484834096592

"FZD7",1.26923817449401,1.4405742989223,12.2961403269101,2.45298290661453e-29,9.30561079573384e-28,55.8562340806638

"COL8A2",1.26890179422556,0.921779938676647,15.3950798736235,1.29871184150227e-41,5.33734491528501e-39,83.8414984423674

"SMOC2",1.26680024292111,2.34173732094245,10.1614332853689,1.53812330787117e-21,2.01385259645611e-20,38.1117815937156

"PLA2G2A",1.26624372082794,5.46340374761077,3.1195747111889,0.00195433584807953,0.00269723870077767,-2.45776929415058

"MARCKSL1",1.26576429072889,6.27693545201936,9.90416530627223,1.18914618734832e-20,1.39963546872063e-19,36.093445104462

"GAL3ST4",1.26463005900707,1.31873797467342,15.1892759025357,8.95259994543116e-41,3.08031898122451e-38,81.9293748190859

"NXN",1.26438994604019,2.09575517829518,9.90349034134934,1.19549845655314e-20,1.4059936140464e-19,36.088188684676

"HDAC7",1.26301098753823,2.92681630815865,16.1593162294718,9.57147829287582e-45,8.33000125547634e-42,90.985837816993

"FOXS1",1.26150842270532,2.10377754454658,11.5202865609953,2.01166864495059e-26,5.09634205514451e-25,49.2205865082199

"ARNT2",1.26126432914334,1.01021612351107,11.20822282904,2.82812950355818e-25,6.02045697915731e-24,46.6076875328748

"USH1C",1.25806352283953,1.61919738930187,6.11177040385519,2.5179586226018e-09,6.74022034040051e-09,10.4930881022891

"GUCY1A1",1.25738264549013,1.63674286304214,12.9921342833475,5.14420127223158e-32,2.94994022568474e-30,61.9588305755261

"GRAMD1A",1.25696159712317,4.08317344731642,11.7556586278064,2.68085275265303e-27,7.49777249064301e-26,51.2133899632665

"SEMA3C",1.25482729252754,0.697025470454622,15.087443155243,2.32220321342134e-40,7.01163194746301e-38,80.9853459035115

"EHF",1.2522003011484,1.77801978903175,8.08547534101993,9.0937792225269e-15,5.04093156977465e-14,22.7524656520346

"ITGA2",1.25049741650282,1.64648740590907,11.1145796785745,6.20967044125109e-25,1.26371491304415e-23,45.8303848879027

"TTYH3",1.2470755471449,4.68645248721739,10.4740255014385,1.23381375417684e-22,1.87223328133808e-21,40.6026946947311

"PAM",1.24686820543519,2.59818033107356,12.2585010095299,3.41109064310428e-29,1.26801723780723e-27,55.5300380848245

"MICAL1",1.24481371203252,2.42873992842643,13.8283316571023,2.66274289817497e-35,2.85473052018107e-33,69.4488590916427

"MCAM",1.24461265606954,4.07928352870413,13.0036813454872,4.63895342527386e-32,2.68098890339557e-30,62.0611502521528

"IL18",1.24447053809416,2.11891945612671,10.9393734785635,2.68173583095359e-24,5.00963151754524e-23,44.3847515679118

"COL12A1",1.24325656134044,1.63823049458215,12.9581619936662,6.97152562086054e-32,3.90695915002393e-30,61.6579917471666

"GUCY1B1",1.24279831172011,2.30052109059278,13.4375732712018,9.31810238824097e-34,7.57479806780358e-32,65.9291510645117

"PDGFRB",1.24257070037943,4.34158225327876,10.9356861880652,2.76524799628576e-24,5.15262520214708e-23,44.3544514417364

"TMC4",1.24244554374517,2.22851474593804,6.19490365705539,1.56536409935364e-09,4.31116192292201e-09,10.9555273843776

"RAB3IL1",1.24175104677729,2.73545375244888,11.0106893498328,1.48046386297708e-24,2.85945990244725e-23,44.971796839904

"ANXA2",1.24101258363014,6.31249256061255,11.8650652598178,1.04415917745156e-27,3.08350000606703e-26,52.1458755019081

"SPRED1",1.24004288932166,2.07642498161031,14.9185688748329,1.12454070773132e-39,3.02501450379726e-37,79.4230447729888

"FPR3",1.23964819844345,2.6344601003266,11.3300304399585,1.01197597019107e-25,2.28582969144686e-24,47.6235055776108

"CSF1",1.23865247225403,3.69148203210927,12.2405406095895,3.99172626707732e-29,1.45104644032946e-27,55.3745329413864

"TTC39A",1.23710177872651,1.59591584802787,9.96885162681207,7.1299042028701e-21,8.61117817807862e-20,36.5981558816228

"TMEM159",1.23707915840289,1.61406094662578,13.0244160537408,3.85267485069902e-32,2.28001297664368e-30,62.2449657119187

"NES",1.23642271901923,3.94464615328431,13.0928084776628,2.0863809774137e-32,1.35385993687876e-30,62.8520231661015

"HID1",1.23598016958506,2.35859660957647,9.56677831900352,1.66191520888942e-19,1.71944304304328e-18,33.4921547672089

"CHST1",1.23553196661471,1.71248550469617,12.2546906658098,3.5267914649677e-29,1.30447199310493e-27,55.4970393630157

"PTGFRN",1.23321158842421,3.88595952156034,11.1829702409405,3.49739245340025e-25,7.31880075644366e-24,46.3977594735835

"PLXNA1",1.23295663133275,2.3809673153093,13.5490924770734,3.38942045502252e-34,3.02473792334111e-32,66.9303022199709

"PXDN",1.23225704211861,2.4603645923427,12.7717130380743,3.67777482768674e-31,1.83826616809545e-29,60.0121311816635

"KLF5",1.22959804638442,2.12732562121172,7.80916849939013,6.09292574833314e-14,3.01689546340659e-13,20.8847802918063

"PKDCC",1.22944461328108,3.31331576996844,9.11831273767405,5.09273951205555e-18,4.37555639261683e-17,30.1190338944958

"HES4",1.22741872466634,2.80603231716738,11.0434212271702,1.12642206657113e-24,2.21320245350862e-23,45.2418725760851

"IGSF3",1.22680198815447,2.17634068100641,8.80341611265975,5.30389631180869e-17,3.91963765900148e-16,27.8115259513717

"PELI1",1.22513525452923,3.25271846434384,12.605119458618,1.61293009683759e-30,7.34255408698837e-29,58.5492398070062

"FAM171A1",1.22485569003441,3.89619613843644,6.25697508804079,1.09419060281569e-09,3.07124833402734e-09,11.304104217007

"ITGB2",1.22409627120775,3.65503674010614,10.2809197063456,5.89172311653838e-22,8.2156497181136e-21,39.0590288134864

"LPCAT4",1.22286071382693,2.04154107290485,14.2112531074707,7.93090956613358e-37,1.03838767284023e-34,72.9280401357799

"ADAMTSL2",1.22182438030406,2.95222949377552,7.21963792360614,3.02277097601829e-12,1.19194820336329e-11,17.0575641763171

"GALNT18",1.22137701654377,3.43781949664274,10.2216631741694,9.48958719647811e-22,1.27519021409531e-20,38.5884965298168

"ARMCX3",1.21893972062962,3.25173127673777,7.28505471762405,1.98105616387265e-12,7.99283499986251e-12,17.4713079447123

"SMARCD3",1.21868266286909,1.9194621399386,11.3915003103199,6.01278339984456e-26,1.39434373668809e-24,48.1381314066853

"NCS1",1.2183803356845,2.10311229974951,11.1565205696659,4.36773113646749e-25,9.01263349568152e-24,46.1781280395592

"CAV2",1.21799751318,3.76200004256204,8.79907313272321,5.47613974107111e-17,4.04288859626482e-16,27.7800663500036

"APLP1",1.21799045501707,1.16280163187481,9.23562619924332,2.10018622206998e-18,1.87974925320782e-17,30.9917579810175

"STMN3",1.21785342347746,2.19622724290596,9.17147440608367,3.41186948886172e-18,2.97808903172325e-17,30.5136532848703

"TNFAIP2",1.21616243120561,4.50501461607906,10.1252918207904,2.05365219877996e-21,2.65824884347765e-20,37.8264795617735

"ELN",1.21588173012904,2.71883322565428,7.07862102975644,7.4462741519128e-12,2.77081554521001e-11,16.1752277078207

"CLSTN1",1.21519118972795,4.40616166215077,10.2964880202286,5.19696953726004e-22,7.3019149386289e-21,39.1828989646903

"FOS",1.21430553093716,4.76288188444535,6.97688858275502,1.41554976035331e-11,5.07464470667005e-11,15.5468962617506

"RASA3",1.21407177307142,2.27257273851841,11.7986096984902,1.85227442787672e-27,5.30065767126424e-26,51.5790077269136

"FUT4",1.21244233223616,1.51121149217632,11.5484805430347,1.58172220872671e-26,4.0557331157906e-25,49.4583165773075

"DUOX2",1.21241825704239,0.942704966756894,7.02148603511656,1.06901624686216e-11,3.8936719281944e-11,15.8214912598799

"EFEMP2",1.21237917004323,2.52813247598927,12.6484224612716,1.09910428308944e-30,5.06580930476895e-29,58.9287769870494

"RTL8B",1.21197844008659,1.91420201170818,12.1000850052218,1.36023349681576e-28,4.58420377799296e-27,54.1617399508618

"RNF145",1.21141330875618,3.57493382823073,14.1890539728759,9.73012490037135e-37,1.2304034008632e-34,72.7255815934604

"SNCG",1.21097163885971,3.8248058297542,6.2243902498023,1.32094009629181e-09,3.67010492481453e-09,11.1207662188701

"ST14",1.21088225591288,4.22133524579854,6.48386052796855,2.88825781197051e-10,8.75113133895222e-10,12.6019156564087

"IFI27L2",1.2105050032893,3.14620114598413,9.73332720478926,4.55018315521115e-20,5.03138712865089e-19,34.7696497425155

"SLAMF8",1.20926850714562,2.34759171420604,10.6192357606939,3.76985538685319e-23,6.11568097022949e-22,41.7736322176873

"MMD",1.20924782665192,3.62379805071222,10.873254294018,4.64402301920684e-24,8.45120794208675e-23,43.8422068928789

"PTGS1",1.20825106020647,1.38819374803287,13.2063380829502,7.51882301651538e-33,5.24721634572382e-31,63.8622273300577

"ITM2C",1.2075179895268,5.3282661911209,8.53616316934309,3.72043633368975e-16,2.47833658518415e-15,25.8946162092194

"KIF3C",1.20747051613361,1.15901908261616,14.576114450778,2.71952188002715e-38,5.07483251583628e-36,76.2681142361901

"VEGFB",1.20646580846457,5.57704255751035,7.16575344128894,4.27239747572761e-12,1.6439563238853e-11,16.7188627774354

"LIMK2",1.20620083487506,3.56640858851198,12.4196284813298,8.29177835548111e-30,3.38280976917974e-28,56.9293038295965

"CLIP2",1.20604547407468,2.54009339553065,9.27242440469118,1.58847258793458e-18,1.44535374775475e-17,31.266937777908

"SRGN",1.2055111590187,5.64569024273566,10.2361764500752,8.44513348877483e-22,1.14839843719139e-20,38.7036016600543

"MNDA",1.20498957213033,1.83670015418026,12.1042806115792,1.31143027864806e-28,4.4399567443016e-27,54.1978820014633

"AXL",1.20489542629686,2.44598295002898,13.3554385068609,1.959012619066e-33,1.53352337032177e-31,65.193572398455

"EFNA5",1.20463632919962,0.709331558742922,11.3071732097119,1.22771256781711e-25,2.731429690354e-24,47.4324842017068

"TPM2",1.20381785110455,4.42642671181124,10.9839461984981,1.85033245272556e-24,3.52779235026736e-23,44.751432766734

"NDN",1.20336678325604,2.68710096652656,8.1429563514795,6.08790627815418e-15,3.46366512315892e-14,23.1467086967585

"ANO9",1.20306412948135,1.14068243017934,10.8267973808987,6.82378468903474e-24,1.21343623166189e-22,43.4620049919881

"SYDE1",1.20185825365118,2.35247060837356,14.0835801440067,2.5672563727169e-36,3.03860464274772e-34,71.7648965939724

"HEG1",1.20181662770811,2.89639877263152,13.0797513111368,2.34577102068771e-32,1.48314881414849e-30,62.7360380494119

"TMED3",1.20163530710926,2.03392274466383,9.1031966613128,5.70562291090441e-18,4.88793809883212e-17,30.007090195344

"LDHB",1.20112666435626,4.29882997954222,10.0967132855313,2.58000489004076e-21,3.30200452838694e-20,37.6012832021354

"HLA-DQA1",1.20057817684252,3.57583620342099,8.29984355694338,2.01669371633884e-15,1.22735432057726e-14,24.2325315899754

"SINHCAF",1.20053665325751,2.33147012659537,11.5532281841845,1.5189178809783e-26,3.90145660574201e-25,49.4983749341799

"GGT1",1.19895228000574,4.10269376941136,6.18916338711265,1.61784902733515e-09,4.4457794129687e-09,10.9234335475929

"SLC25A24",1.1987314122465,1.46193587657146,11.6994920027802,4.3436689008756e-27,1.18788505339103e-25,50.7361813988609

"EPHA3",1.19771569841739,1.18406792041526,11.8803995149211,9.14617657831801e-28,2.71722253968303e-26,52.2768772360267

"EHD2",1.19682984270244,4.35813024562524,12.1310794127994,1.0383294131994e-28,3.58090528398253e-27,54.4288594643667

"FPR1",1.19668910875006,1.32959037890229,12.2348477470119,4.19556851734621e-29,1.5214077503465e-27,55.3252626694119

"NT5DC2",1.19645898117079,3.3189739491687,8.32089753451377,1.73695625715677e-15,1.06322994723353e-14,24.3793259504751

"SLC2A1",1.19645661361947,2.12887136719776,9.13617716822883,4.45207793958107e-18,3.84745871005268e-17,30.2514812320473

"CTBP2",1.19559076883147,1.32370355820188,13.6890778770515,9.48776552138118e-35,9.29612522442613e-33,68.1908309421226

"HLA-DPA1",1.19511853149904,5.02706156797417,7.6575504209193,1.69730522407682e-13,7.89922327468278e-13,19.879607255766

"PLAT",1.1934861159422,2.18727429884711,11.2330243764066,2.2951216621236e-25,4.92834905531476e-24,46.8140902207795

"BCL2A1",1.19267659479362,1.80949026883623,10.0188917979751,4.79381407639494e-21,5.8907374800883e-20,36.9898778085952

"ITGB1",1.19246142280067,6.0996566562547,9.14911023928813,4.03876511438279e-18,3.49845022642233e-17,30.3474688017144

"PDLIM3",1.19227432519799,1.80309602366726,13.1729442347777,1.01546441809418e-32,6.95546114152932e-31,63.564762882616

"ARNTL2",1.19218667511975,1.96323705826721,11.2325180283305,2.30493275952505e-25,4.94224350393813e-24,46.8098740913604

"TNFAIP6",1.19046695898909,0.67586454916061,13.0892069721168,2.1549278660988e-32,1.38311432665364e-30,62.8200273091549

"ITGAM",1.19032679084145,2.0193189678027,10.816699345581,7.41833125765818e-24,1.30971612120588e-22,43.3794733101681

"SERPINH1",1.18948248241401,5.43524290290374,11.9538031358705,4.84642036835855e-28,1.51591520824238e-26,52.9049986020302

"HLA-DRA",1.18911230326147,8.63706032657285,7.69312065613166,1.33634027297224e-13,6.30256752904823e-13,20.1141521796626

"VSIG4",1.18847798757651,2.81217615914875,9.02445181706202,1.02939811321282e-17,8.49885328403108e-17,29.4258341167716

"C1orf116",1.18794839466379,1.0420686966393,8.56522197000579,3.01587739873478e-16,2.03930101070754e-15,26.1011512607656

"LTBP1",1.18717876503464,2.71215551709592,9.18554009777247,3.06805648653379e-18,2.69708233620127e-17,30.618303015264

"GLIS3",1.18667249208841,1.34634474232144,11.9543208605571,4.8247311773403e-28,1.51421489301913e-26,52.9094348253245

"MARCKS",1.18580019580082,5.40742150478496,10.1025088074792,2.46341404219588e-21,3.16923571776418e-20,37.646922500614

"NGFR",1.185511348846,2.17684657864956,8.06607733513987,1.04080829308882e-14,5.73724243526417e-14,22.6198595701563

"ASAP1",1.18421263917303,2.96594780649068,12.9301757663307,8.95330317681588e-32,4.90607853707374e-30,61.4103796945732

"MYBL2",1.18405505221402,3.58384799524695,6.19476660096098,1.56659760435739e-09,4.31375610580079e-09,10.9547608233054

"MTHFD2",1.18403288609831,1.7310478392031,14.0842264516223,2.55205535800795e-36,3.03860464274772e-34,71.7707769915221

"HAPLN3",1.18294527024161,1.48803628625191,12.6598096057657,9.93571236021584e-31,4.62260579777967e-29,59.0286658311127

"LRRC32",1.18235049467213,3.66507128869771,10.9963098696327,1.66912275789858e-24,3.1987916066204e-23,44.8532762728501

"TSPAN13",1.18202737945158,4.51891232365613,6.11287016586869,2.50225559595128e-09,6.70412461349241e-09,10.4991725130494

"ERICH5",1.18146288402161,4.77931661749401,4.74056799317763,3.05390518296007e-06,5.57602458125315e-06,3.63071064701002

"NEURL3",1.18096349587962,2.03636111147278,7.99823840506837,1.66592421675164e-14,8.94640609322705e-14,22.1578585120729

"LAMC1",1.18076948771913,5.24534694393462,10.6541860285085,2.83037544056011e-23,4.66318537228139e-22,42.0567345107251

"CXCL12",1.18036267028285,3.51345045969255,7.2518125682286,2.45638333179143e-12,9.79309927077721e-12,17.2607113933349

"C11orf96",1.17258667932007,3.42568779019379,8.77146630734535,6.70812425619683e-17,4.9034930024917e-16,27.5803245670583

"VNN2",1.17241664989027,2.90281209143467,6.03557506863321,3.87587803768272e-09,1.00992630446488e-08,10.073714711496

"PTAFR",1.17195161857015,1.80641113144159,12.0682779929911,1.79405808480224e-28,5.89846430325538e-27,53.8879199124437

"FCGR2A",1.17139684463789,2.46433959986395,11.9731647180409,4.09777288238093e-28,1.30099892263575e-26,53.0709589378249

"ANXA1",1.17115560991356,3.60716757248168,11.386783676187,6.25812307859046e-26,1.44896605552028e-24,48.0985968438305

"ACSL4",1.17101199185836,5.61856335469406,4.83168511263823,1.99251714781543e-06,3.71462842766246e-06,4.04014533652168

"TMEM173",1.17083138457196,3.05117801591578,13.0206387852934,3.9852840747751e-32,2.33977293199593e-30,62.2114718761188

"ARHGEF25",1.17040011678727,1.55016033705317,13.907632782815,1.28917729965079e-35,1.43408858258147e-33,70.1670370837922

"EDIL3",1.16985955179082,1.56891364911983,11.4651740736381,3.21624360252065e-26,7.80070886873655e-25,48.7566639115559

"TMPRSS3",1.16892499504759,1.49615546942007,7.16686145526207,4.24218790046096e-12,1.63318162860577e-11,16.7258081924276

"FERMT3",1.16886318747618,3.39761760414725,11.6814592835049,5.07050272782148e-27,1.3714458474976e-25,50.5831893327384

"RNASE6",1.16861936958606,3.55963125271629,10.1788598515726,1.33774714244088e-21,1.76399010449313e-20,38.2495503380562

"CSF1R",1.1683890841696,3.44700981617267,10.2499072320306,7.56241477913839e-22,1.04079931774281e-20,38.8125839415175

"HCK",1.16734007360339,2.66655931528124,11.102349443208,6.87982875007021e-25,1.38674477326006e-23,45.7291022486097

"LY96",1.16576736044147,4.21278609949882,6.92332182443046,1.97989026063598e-11,7.0010698867374e-11,15.2188457320778

"ALDOA",1.16573781543857,7.09435341586719,10.8523847193236,5.52099993485136e-24,9.92505395335673e-23,43.6713080233882

"PHLDB1",1.16532699890391,1.29661383958025,16.3892684813688,1.07987142588978e-45,1.45242706782175e-42,93.1470884445839

"CYBRD1",1.16408356038245,3.12666432650166,8.95913776272161,1.67538387058405e-17,1.33336763664825e-16,28.9461514845186

"CXCL14",1.16316875528521,1.2165510736759,7.183999489048,3.80084205888865e-12,1.47555650121379e-11,16.8333382494369

"BLVRA",1.16314613008204,4.55893261885861,9.27653012299691,1.53967956331634e-18,1.40788375397189e-17,31.2976826343305

"DUSP4",1.16295314924991,1.1718803876222,12.0922052675801,1.45683152411272e-28,4.86542266348707e-27,54.0938762541328

"SRC",1.16269541794022,3.35758903005354,8.49552301776752,4.98630912270753e-16,3.27586338678765e-15,25.606552689993

"PODXL2",1.16127497956096,2.06317750516274,5.36499219922861,1.43658395180423e-07,3.07098100952803e-07,6.57233058202991

"TSPO",1.16123929838691,6.06430779284526,9.48196317700143,3.19844711400203e-19,3.1866010135798e-18,32.8466562178922

"SEZ6L2",1.16106506361588,3.09502969690668,4.65230603438551,4.58947247653161e-06,8.24344364335137e-06,3.24068265404126

"AMIGO2",1.16023601584425,1.76185631718613,10.5579236284485,6.22571051068971e-23,9.87238874658674e-22,41.2781819078796

"TES",1.15966398015251,3.57168779026415,10.1018635378915,2.47613184267536e-21,3.18245254038483e-20,37.6418403227356

"CST2",1.15939601139421,0.873461638911949,11.195314312124,3.15257281327823e-25,6.66318782463591e-24,46.5003485043088

"PLEKHB2",1.15921493085001,3.73627513168311,14.8512005545994,2.10748222718994e-39,5.11150812315986e-37,78.8009681998837

"SLC2A3",1.15820445242287,2.30053531607268,10.9637059221655,2.1901532763148e-24,4.13307624018846e-23,44.5848313979737

"LINGO1",1.15717728235529,1.66216786999319,8.64002914585995,1.75296414610066e-16,1.21589800944957e-15,26.6349836948757

"ZNF532",1.15670000510829,1.55312882149353,14.4484091937504,8.87828693938902e-38,1.41241134697054e-35,75.0964390005636

"HAVCR2",1.15634945038147,2.09369726492702,11.7852765509076,2.07767294769633e-27,5.88873012666038e-26,51.4654464347917

"SCPEP1",1.1562773345136,4.53893843064314,8.69858129278494,1.14399846262471e-16,8.12510521602679e-16,27.0549544115518

"NRGN",1.15626494828654,2.60343177868254,13.235502064909,5.78194124481128e-33,4.17286930326746e-31,64.1222293569795

"MPZL1",1.15611683059389,4.53828806888611,14.4491115607119,8.82076758009885e-38,1.41241134697054e-35,75.1028755800623

"MFSD6",1.15596870276623,2.28792983493031,11.4265739876273,4.4649435887908e-26,1.05863526275897e-24,48.4323600079603

"WIPF1",1.15532058587875,2.39773499201306,12.7810056963698,3.38593923705725e-31,1.72147666708804e-29,60.0939486361496

"LY6E",1.15263683620484,5.80439013790799,5.44783058736387,9.36271113220637e-08,2.04429325857428e-07,6.98606399212784

"DDIT4",1.14999651702755,5.72449619758543,8.38258421747077,1.11985263632214e-15,7.03832614884708e-15,24.81087753214

"CEP55",1.14913461991373,1.75524562447395,10.3316898593372,3.91178296559064e-22,5.5917709155472e-21,39.4633631605738

"HMGA1",1.1488746779322,6.13762120607344,8.65198174051856,1.60694711580197e-16,1.11881329780189e-15,26.7205630384334

"C5orf30",1.14879108616063,1.50247281997781,10.443801118403,1.57747674946964e-22,2.37182606792717e-21,40.3600577926521

"GAS7",1.14877791363919,1.20663929997215,14.7786353911088,4.14250301326803e-39,9.5763018877032e-37,78.1316725842655

"PTHLH",1.148075572892,0.833509022434713,10.0180480377802,4.82605349447419e-21,5.92543248553905e-20,36.9832634808999

"RARRES1",1.14762803012778,2.66733907889676,7.44211190551886,7.10430573413974e-13,3.03342578171999e-12,18.4759644888327

"ARHGEF17",1.14688814665261,2.20342350723675,12.6421926276236,1.16149425106427e-30,5.33674144239005e-29,58.8741432237751

"WFDC2",1.14659613090313,1.7911366269605,5.31723906385646,1.83435026184337e-07,3.87592289688269e-07,6.33628890935965

"CAVIN3",1.14636475472394,3.37620025301642,10.1644347524826,1.50160176083167e-21,1.96777662103672e-20,38.135500827403

"COL8A1",1.14583839289311,1.29696617690513,12.8245489509026,2.29775061621271e-31,1.2012445359317e-29,60.4776265040386

"BAIAP2L2",1.14430874589843,3.16821708425275,6.57006418505698,1.72501479209229e-10,5.38089686886052e-10,13.104636277159

"ADAMTS1",1.14404884582339,2.89169106008012,10.0957676682299,2.59954153994471e-21,3.32126226973074e-20,37.5938379350234

"FZD2",1.14347972180851,1.03667814774626,13.7445149923302,5.72380758247808e-35,5.72187386370021e-33,68.6911733588312

"RASSF3",1.14289525873296,3.9456282195938,9.75955788896119,3.70611659361934e-20,4.14138935064941e-19,34.9720338957822

"PLXDC2",1.14186561050793,1.60279538675095,12.1923412510692,6.08365331664816e-29,2.15845685419207e-27,54.9576824680321

"FHL3",1.14176424094235,3.8412098126975,13.2255749157827,6.32286427666855e-33,4.51916796972518e-31,64.0337045399213

"JUNB",1.14122485876996,6.71110075111942,10.1498071603504,1.68810903843858e-21,2.19662033629716e-20,38.0199425236254

"CHST3",1.14120358940135,1.39195395077832,13.1611597464303,1.12900347076747e-32,7.62721751141765e-31,63.4598529385432

"SNAP25",1.14113018414894,1.48069151339343,7.63880681048049,1.92462173969157e-13,8.9011499339596e-13,19.7563307593859

"BAMBI",1.14076432118606,4.70283580421469,6.36823130840812,5.71989333540815e-10,1.65932984112478e-09,11.9358830495623

"SERPINB9",1.14073899157655,2.7176055297238,12.1553169772869,8.40498833170904e-29,2.93282552753857e-27,54.6379480685018

"ACP5",1.1407075311034,4.6145482882617,7.04928324271075,8.96797089442411e-12,3.30085134236146e-11,15.9933182290621

"ARHGEF2",1.14048596177064,2.93365466281793,11.2457362660115,2.06198008170273e-25,4.4600870334491e-24,46.9199665065024

"NPNT",1.13932734341908,2.10913885168225,7.1167395333563,5.84321557722344e-12,2.20987664787886e-11,16.4124373783185

"FAP",1.13931139322981,0.786049705549014,16.5823674642464,1.72229117203647e-46,3.18516223628495e-43,94.9654501153889

"NNMT",1.13864881323751,6.87038683407486,4.19762662584933,3.38761534519846e-05,5.56391752133783e-05,1.33564559633039

"INAVA",1.13864764765284,1.4535309373565,7.67089078198939,1.5518633011369e-13,7.2657650444052e-13,19.9674796767204

"LOXL2",1.13741132786304,2.78235360877399,13.0823920552575,2.29083820794498e-32,1.45463310242687e-30,62.7594920827774

"HILPDA",1.13715029054312,2.71006194583392,11.2831084488474,1.50442708251958e-25,3.30728063683168e-24,47.2315714874633

"MSN",1.13694976383171,6.08625335137852,11.1788376220874,3.62102032303389e-25,7.56680729933424e-24,46.3634264783302

"ORAI2",1.13632445458839,1.43509749315063,15.4408187866175,8.44992569077241e-42,3.79924013648184e-39,84.2671986380729

"MELTF",1.1358485296354,1.54271299474826,8.62692136754638,1.9282206686691e-16,1.33184055989539e-15,26.5412231498773

"B3GNT9",1.13508714207453,1.61747317000372,12.0371237999706,2.35214181969942e-28,7.59823978656178e-27,53.6200190896618

"AREG",1.13403407151187,1.07790061921346,9.8360661365601,2.03344612849168e-20,2.34671103518209e-19,35.5641525898975

"CDH6",1.13310421278406,1.1505343247399,10.8076356578833,7.99569886458725e-24,1.39995697871679e-22,43.3054290765513

"IL7R",1.13286897990316,1.67767673305082,9.0010056545463,1.2263845474071e-17,9.98040277568964e-17,29.2533844954909

"PODXL",1.13231711668325,3.7050320754133,10.544637389902,6.93927139432291e-23,1.08987813459668e-21,41.1710192298858

"STX3",1.13167841772781,3.12668074259816,11.6607921696439,6.053479875625e-27,1.62838608654312e-25,50.4079786398678

"EEF1A2",1.13160993689689,4.33564074844184,3.26218581026205,0.00120917368820877,0.0016982841007261,-2.01605389452338

"ARPC1B",1.13118340843423,6.08744942231796,12.4354892426352,7.21125782283865e-30,2.98853107812039e-28,57.0674439281295

"CCN3",1.13049617390102,1.56865422856806,12.1168122646355,1.17580538950754e-28,4.0175613713081e-27,54.3058646623021

"SMIM3",1.13010960597105,2.62196048110416,10.0120623720038,5.06102636306182e-21,6.20877985418736e-20,36.9363502826402

"ARMCX2",1.12969843159523,1.44586723851884,12.8260424964226,2.26737721849036e-31,1.1895690052328e-29,60.4907954989114

"DEPDC1B",1.12870121422168,1.8413917693828,8.99325251894807,1.29940868232646e-17,1.05168224589825e-16,29.1964219370828

"CDCP1",1.12826935528955,0.921075864132251,9.88329436853709,1.40197336718602e-20,1.6409965164175e-19,35.9310041443229

"ADRA2A",1.12696185217799,1.07601674680513,9.92783484416815,9.86371860677886e-21,1.17010637647531e-19,36.2779064493064

"CCDC3",1.12617130794048,2.94856209141741,9.4088068874505,5.61038307325423e-19,5.42166019391224e-18,32.2926817509205

"GPX2",1.12566508844064,8.06071736383825,4.22053286999627,3.07543685564335e-05,5.07428217678637e-05,1.42739284300747

"C12orf49",1.12540292857851,2.71030622902503,12.075772281121,1.6808221352944e-28,5.55084006510729e-27,53.952408978566

"THBD",1.12534230953356,2.54520122081338,10.8081775143855,7.95995947732492e-24,1.39535071643391e-22,43.309854783342

"AFAP1",1.12484418188413,1.53704214364542,13.8610046623173,1.97517085318959e-35,2.14872446859853e-33,69.7446040225827

"STK17B",1.12461064676675,2.01697047812952,12.5000217250616,4.08305868231074e-30,1.73588658634446e-28,57.6302226692101

"CAVIN1",1.12440583034521,5.52331250926718,12.2795437045497,2.83699300611753e-29,1.0680231940333e-27,55.7123507309996

"FRZB",1.12411461892642,2.51534195092474,10.1003537209243,2.50614462835745e-21,3.21303377613072e-20,37.6299496364646

"HNF1B",1.12406734664262,2.87638139487742,8.18438966751845,4.55340984591118e-15,2.64394421782794e-14,23.4320844940457

"FMNL1",1.12320610838801,2.05819561353003,12.2641581338681,3.24625104113469e-29,1.21283545842393e-27,55.5790383333149

"TLR2",1.12315675714964,1.88942866740461,9.94162921080475,8.84446305827929e-21,1.05783210143284e-19,36.3855252222369

"DUOXA2",1.1226766127369,0.960742692193088,6.71929255363337,6.98371880554257e-11,2.29813433558724e-10,13.9872329238182

"HK1",1.12226911612824,2.87444264288357,10.9638841694791,2.18690511449286e-24,4.13221726295298e-23,44.5862979091795

"EDNRA",1.12133627211388,1.55458623884228,14.1617276501909,1.2513204026606e-36,1.54277377978029e-34,72.4764870412698

"ME1",1.12001492520146,3.66040637307924,6.37421503650078,5.5224303842454e-10,1.60708807110367e-09,11.9701150814564

"LSP1",1.11914006449312,3.21965180936638,9.61803633463179,1.1170221583467e-19,1.1796104805667e-18,33.8839291780317

"MYH11",1.11888482367406,1.7444690810447,9.67908956088855,6.94781960013711e-20,7.4649957141633e-19,34.3521928835542

"ECT2",1.11824066126964,2.53659794628981,9.15617942552981,3.82916815528675e-18,3.32467974515654e-17,30.3999713318317

"LARP6",1.116752314814,1.28749616144001,10.3424141101717,3.58710328259117e-22,5.14754539921788e-21,39.5489106974832

"NBL1",1.11513083414847,1.98000203810395,9.65727028522417,8.23438949433175e-20,8.77721848477221e-19,34.1846425986932

"F13A1",1.11465449114722,2.04861796461185,7.84448224433637,4.79000577577298e-14,2.39986913147854e-13,21.1209205404934

"SLC9A1",1.11437828116199,2.19531190945231,15.2480630494632,5.16059522324554e-41,1.86221966653458e-38,82.4749974148115

"TMEM54",1.11372691372641,3.9534571034061,6.9608645972692,1.56531082617506e-11,5.58177239654375e-11,15.4485600933034

"CD52",1.11283792814204,4.22152423872154,7.79788511665615,6.57876308380195e-14,3.24334554564644e-13,20.8094893749281

"FCER1G",1.11263562007861,5.20530424849742,8.86031045676089,3.48637185499672e-17,2.6424626841535e-16,28.224584711705

"SDC3",1.11262073790055,3.9197250141575,11.0803840738492,8.26905959612631e-25,1.65325319898228e-23,45.5473381425946

"DPT",1.11245203364626,1.91014685391276,5.96637218758615,5.71385196744796e-09,1.45576786392961e-08,9.69656212025064

"CCN1",1.11144724819769,5.15314423262016,8.32380579651218,1.70146217035643e-15,1.04409509790225e-14,24.3996230953235

"CTXN1",1.11138967212588,0.976135599009483,11.0947561451496,7.3316171674129e-25,1.46980048769477e-23,45.6662473397308

"CAPN6",1.1111531017061,0.738005049921927,7.57575995756349,2.93270567491795e-13,1.31842541660319e-12,19.3432813289653

"TRIM47",1.11045251141683,4.36110931118244,9.00964293354708,1.14981347413026e-17,9.4403387068575e-17,29.3168796535664

"VCL",1.11027445227598,3.4189183906039,13.7896960485798,3.78971953325401e-35,3.9485141193305e-33,69.0994221335842

"TUBA1B",1.11004933553232,5.78196516848901,8.47635684874004,5.72301258457809e-16,3.72840031654922e-15,25.4710192877324

"CD93",1.11003963311136,3.74129882165369,11.8363343418495,1.33802891437857e-27,3.88922156939705e-26,51.900626528793

"PGF",1.10948984345668,1.93328587694443,13.2442268120354,5.34477791452557e-33,3.89536892834512e-31,64.2000509659572

"REG1A",1.10878002672191,1.80367220514796,3.575148435133,0.000396812791108871,0.000584745542276469,-0.981702535960966

"SUSD4",1.10872061048163,2.37233130489899,5.58668386453265,4.51543765618058e-08,1.02478754599159e-07,7.69160819665784

"CORO1A",1.10863228990737,3.34881362685553,8.96005981153073,1.66392868273349e-17,1.3271064615117e-16,28.9529077752871

"SOCS1",1.10821640289319,2.91677194899723,9.12492468813817,4.84561422754176e-18,4.17292564007452e-17,30.1680360879505

"TRPV4",1.10789000402236,1.84664969672317,7.52924589102754,3.99526212073482e-13,1.76015436137927e-12,19.0401442838435

"IL15RA",1.10767381249022,3.53183865350782,11.4599779080866,3.36155355030485e-26,8.1000300939349e-25,48.7129776743452

"LAMA2",1.10612286712125,1.36737228825157,9.55740429735988,1.78692496254596e-19,1.83849477196575e-18,33.4206427538098

"TCIM",1.10567031149356,5.03707064825347,7.50555067870449,4.67440152295689e-13,2.04487789864421e-12,18.8862446993054

"C15orf39",1.10516335630812,3.21863571470933,12.7777593941019,3.48516768696643e-31,1.75384543975062e-29,60.0653639127013

"S100A8",1.10464764215946,2.36042269943537,7.18248227688202,3.83801660488153e-12,1.48920681010287e-11,16.8238109299182

"HIST3H2A",1.10455257145142,1.7193878588885,6.00206800856028,4.6791980770311e-09,1.20818037608508e-08,9.89065720538636

"IGFBP3",1.10443953353102,5.96937705384893,7.72155716686366,1.10321721270472e-13,5.25671454491668e-13,20.3022216532057

"RFLNA",1.1037143195688,0.73941721375975,10.2051320744892,1.08363167795858e-21,1.44435411490064e-20,38.457498271554

"IFT57",1.10330072995554,2.40103893720665,10.7780605861077,1.02087717281152e-23,1.77067734721528e-22,43.0640428763656

"B4GALT5",1.10219700317307,4.50109281994716,12.0255661958014,2.60056875805908e-28,8.36422060336612e-27,53.5207087488517

"SIGLEC10",1.10109372598875,1.22276786696727,13.6329415864013,1.58171031180269e-34,1.50976800407231e-32,67.6848365348011

"EGR2",1.10106308497011,1.29521678603999,12.316031043592,2.06038650015706e-29,7.90589457152985e-28,56.0287809067231

"PRKX",1.10060856271762,1.70241951305225,10.8460826284337,5.8168652715634e-24,1.04189493574795e-22,43.6197337984652

"ENAH",1.09985868488874,3.57532357144859,10.3891636095463,2.45731371923434e-22,3.59603921622869e-21,39.9223968713006

"RCC2",1.09952987419673,4.75806634277559,12.1224327438719,1.11960652926614e-28,3.84328969849015e-27,54.3543105848933

"PDE4A",1.09948850897358,1.75045341916926,11.6677952749911,5.70079993392269e-27,1.53630847035312e-25,50.4673334212619

"CLIP3",1.09903877749197,1.78213217787307,12.7270575020563,5.47008152452519e-31,2.67095234836139e-29,59.6192769392837

"PLSCR1",1.09798141081846,3.79192707562357,9.8979685572733,1.24874660648405e-20,1.46745083740521e-19,36.0451944704798

"NRARP",1.09788322746431,2.32123931984945,11.4913393812436,2.57425539519879e-26,6.40102665075061e-25,48.9767870594719

"RASEF",1.09684370649092,1.72287816396847,8.13388652366796,6.48666482397756e-15,3.67842874935791e-14,23.0843730911431

"ANXA4",1.09652142068841,5.2380363596644,9.34328940955514,9.26007335803279e-19,8.73742253393464e-18,31.7987725767966

"LZTS1",1.09546561831619,1.58060974604407,14.6397870435277,1.50612872802496e-38,3.01123980150395e-36,76.8533088826404

"TMEM45A",1.09539755213266,3.00014982590036,5.95377119440159,6.12998856275861e-09,1.5540298284101e-08,9.62827184763181

"PKIB",1.09506128360498,1.92861333770273,6.95293826110241,1.64504493812828e-11,5.85199323385619e-11,15.3999816491499

"PDP1",1.09495716540702,1.73905471478217,10.3677837642053,2.9217333838999e-22,4.2504469434414e-21,39.7514775200607

"FOXJ1",1.09474984675144,0.924789103215048,7.44832718572144,6.81959500001324e-13,2.92028677352231e-12,18.5160477095299

"IL34",1.09472450250988,2.21559280746666,9.59014134974101,1.38684949886194e-19,1.44496044617341e-18,33.6705677556153

"YWHAZ",1.093671361815,6.42067123848527,12.8129461447985,2.5479459847483e-31,1.32735425508278e-29,60.3753411186711

"PCSK1N",1.09365572269165,1.2642568323412,5.68733225736476,2.63765628579848e-08,6.17079771479894e-08,8.21236862965072

"GPR137B",1.09282782920753,3.70923548728183,8.71854095266317,9.88693183468558e-17,7.05177837729626e-16,27.1985425650664

"GPSM3",1.09196257319539,3.72695662271296,11.0349671056548,1.20885724535025e-24,2.36574642129061e-23,45.172078018507

"PACS1",1.09113880646306,3.5630496940311,14.4201502601853,1.15311048359131e-37,1.75879068090035e-35,74.8375406576395

"UNC5CL",1.09106006281299,3.98276037764601,7.61909385040335,2.1961072717574e-13,1.00654916622214e-12,19.6269149821529

"ATP1A1",1.09028017363283,7.18371399965614,10.2229596220177,9.39128957321578e-22,1.26427779104393e-20,38.5987750095561

"POF1B",1.08985935036522,1.07750127756543,7.52713651689872,4.05154723585006e-13,1.78347638662308e-12,19.0264295857855

"SFN",1.08978778934995,4.44045711709375,4.15591670547008,4.03522665891217e-05,6.58299464313656e-05,1.16974832465461

"LGALS1",1.08875129940076,7.64749423912267,9.78890034354012,2.94494220978727e-20,3.3259862590689e-19,35.1988046330727

"ADAMDEC1",1.08830192558613,0.945761461864521,10.0563236460706,3.55964522016417e-21,4.4631314434177e-20,37.2836277784379

"SLFN13",1.08794111523967,1.08413642811483,8.85462423823864,3.6359552340188e-17,2.74846631381566e-16,28.1832247859673

"PLEK",1.08793095207541,2.81184562344871,8.42944424740665,8.01194177051199e-16,5.11816401099849e-15,25.1401449018917

"PLCD3",1.08776924422832,1.55900499493554,11.4199446424994,4.72347518708813e-26,1.11457440818132e-24,48.3767145132832

"MYADM",1.08761032230774,5.15842751203091,11.0008836423689,1.60665687456931e-24,3.09109082695097e-23,44.8909664519489

"EMILIN2",1.0870404794371,1.66442287316705,12.4308057004367,7.5148388756193e-30,3.08839003235521e-28,57.0266449805159

"PALLD",1.08638623850189,3.59298120147963,9.07538276843434,7.03040956350839e-18,5.97443477840934e-17,29.8014172108313

"KLF4",1.08587102207854,2.5999894683016,8.58714122690384,2.57338257322765e-16,1.7512969259845e-15,26.2572508002712

"PYCARD",1.08551178706432,3.97728461572889,7.11989942199307,5.72671615366487e-12,2.16914402185028e-11,16.4321444835671

"DKK1",1.08406071678848,1.72706664709057,4.43115332178298,1.23910606881072e-05,2.13144684200146e-05,2.29212692674741

"CRMP1",1.08332796349538,1.50291305534087,10.3061950228026,4.80564266839354e-22,6.77137935989356e-21,39.2601853459609

"BTG2",1.08314151997858,4.59416857230867,8.54105894391063,3.59125255887595e-16,2.39984560110974e-15,25.9293801132721

"THEMIS2",1.08256468737204,2.43470727812637,11.0678081579212,9.18660099146852e-25,1.82437263984935e-23,45.4433523271476

"DTX3",1.08192850962882,2.06340221483177,10.2429449066686,7.99790615032318e-22,1.09767181348823e-20,38.7573133357729

"TMEM65",1.08189126910576,2.42001869404508,11.7971161037688,1.8762610310302e-27,5.3486092397094e-26,51.5662836062961

"RTL6",1.08171527025497,1.91197863034163,10.6492390189214,2.94765247720734e-23,4.84023511656855e-22,42.0166333160715

"NIBAN1",1.08157701571146,1.67019561332592,12.5420909309721,2.81630091469824e-30,1.22912011896638e-28,57.9977276516196

"SLC39A10",1.08093798392246,2.11530928418881,12.4795427723335,4.8913846344484e-30,2.05591010416659e-28,57.4515022229007

"TPBG",1.08078759219615,0.767736630312314,12.0994685382891,1.36755505834772e-28,4.5984038836942e-27,54.1564299929354

"MMP10",1.07935693950714,0.638977088786745,9.48021554102293,3.24177086190615e-19,3.22759084131234e-18,32.8333919913963

"EPS8L1",1.07905165640211,0.873334639578068,9.04730773286783,8.67636078489914e-18,7.24826413396854e-17,29.5942163312185

"CHI3L1",1.07758542260597,6.16315342913099,3.08669174061725,0.00217774670543869,0.00298967825062312,-2.55696548725148

"PDPN",1.07753729303693,0.711324739834224,12.7803262906043,3.40647064676091e-31,1.72598401434341e-29,60.0879660242366

"LFNG",1.07743761389036,2.16841045486047,11.7304006656453,3.33101735819791e-27,9.26360936363496e-26,50.9986631384216

"PPP1R9B",1.07741566130553,4.1729291882104,12.1724590077658,7.23714410589193e-29,2.54331465669052e-27,54.7859324248379

"APP",1.07659873373336,7.02564684641118,8.15551952855484,5.57529864039804e-15,3.18973485632982e-14,23.2331329951209

"RTL8A",1.07616889031706,5.27850741633504,8.5494715849781,3.37955615938903e-16,2.26965653101047e-15,25.9891475926645

"MBOAT4",1.07601294357826,0.803969377750823,10.7436152187925,1.35637875013027e-23,2.31193820370706e-22,42.7833369782586

"ZDHHC1",1.07589747360974,1.83977284453004,10.755224119252,1.23257113461849e-23,2.11308110506148e-22,42.8778895369098

"RPP25",1.07589616027244,2.44131387994751,9.41361508932168,5.40737217346952e-19,5.24259969242998e-18,32.3290117252617

"MTCL1",1.07586507360367,0.932913913905794,12.3479085731591,1.5575903831765e-29,6.09644172462867e-28,56.3055490495178

"WWTR1",1.07552922863804,3.29593895934161,10.344214590593,3.53528191334269e-22,5.08304139046697e-21,39.563277911959

"C3AR1",1.07547652935528,2.6975182734293,10.3482505355298,3.42180645501303e-22,4.93908551238222e-21,39.5954883220478

"LIMD2",1.07443564196588,2.76254386614722,8.90277966134727,2.54610457708018e-17,1.96605517838733e-16,28.5340345988912

"PTPRS",1.073978355238,1.52165751985752,7.21892613984271,3.03665489910796e-12,1.19710389641093e-11,17.0530777046811

"ARHGEF3",1.07386076612708,2.27039809735374,10.4459225947582,1.55052199856869e-22,2.33604612717147e-21,40.3770763762657

"TYROBP",1.07348709772282,6.10707767156806,9.12063990953656,5.00436744983996e-18,4.30212762465905e-17,30.1362783823542

"RAB25",1.07207955122388,0.850073102923923,5.65672838539934,3.10855699582616e-08,7.22562462737597e-08,8.05319677034606

"ZNF703",1.07175663046266,3.13638469100394,7.43552908548461,7.4186301770294e-13,3.15761316079258e-12,18.4335378217612

"ASAH1",1.0713825319469,5.01566745390023,11.897019017749,7.92236140288111e-28,2.3726991286564e-26,52.4189431170829

"JAK3",1.0713331735203,1.67393255797834,11.1990072838615,3.05614661731313e-25,6.47789243598106e-24,46.5310507576241

"SIRPA",1.07080397968616,4.85367426507293,11.057967911873,9.97459132461716e-25,1.9720448375632e-23,45.3620276756848

"LRRC1",1.06954643621966,2.31941190883251,7.94528590478891,2.40050679547024e-14,1.25629635794065e-13,21.7991412635229

"EIF5A2",1.06865580749147,1.67440263384415,9.22499388869216,2.27638349332131e-18,2.03376170191358e-17,30.9123751329333

"SELPLG",1.06862686223779,3.10189769148169,9.68653520542654,6.55615030751559e-20,7.10089632501414e-19,34.4094191517297

"GLIPR1",1.06813791261451,1.84011573393286,10.2266080405969,9.12005943835067e-22,1.23000254685869e-20,38.6277042258079

"H2AFY2",1.06803692000669,3.63876878194208,5.78753809863227,1.53292661924621e-08,3.68654898110334e-08,8.7385611301077

"SH3BGRL",1.06786141029431,4.73584231975961,9.39643873598856,6.16798439898746e-19,5.92567072617009e-18,32.1992820147947

"C1QC",1.06780481185851,6.86094444263389,7.5422448232676,3.66503538642338e-13,1.62493852388774e-12,19.1247225112936

"SLC44A2",1.06750781570774,4.02166467288796,10.3145118273258,4.49374421720983e-22,6.37439556027032e-21,39.3264348394836

"CHSY1",1.06722730346115,2.7957065241196,11.6083688623882,9.48267005865297e-27,2.45702457999598e-25,49.9641826341406

"PDZK1IP1",1.06709087976499,4.55574826618041,3.36481019235275,0.00084681224957057,0.00120676047316476,-1.68667413411867

"GRAMD1B",1.06696451825321,1.27431958659672,8.96648281989382,1.58625628282249e-17,1.26857630834372e-16,28.9999845390857

"PKD2",1.06605339287547,2.53134007734174,10.578218518017,5.27406900016958e-23,8.41745963942923e-22,41.4420124489245

"EVC",1.06549534194564,1.60191731572614,7.70453986676839,1.23739835406031e-13,5.86396817691298e-13,20.1896149578278

"CYS1",1.06512996355571,0.883541954414494,8.4905449506981,5.16810565441623e-16,3.38627649057078e-15,25.5713306160882

"MEGF6",1.06443646604252,2.03070565933804,8.15274267810386,5.6847955618893e-15,3.24676114402135e-14,23.2140226199923

"TUBA1C",1.06427033581662,4.29041480694296,10.4882525560485,1.09891045050673e-22,1.68132162515482e-21,40.7170377294959

"CD3D",1.06413718201329,3.03516594699174,6.99056805523089,1.29891904440185e-11,4.6735183029974e-11,15.6309813516146

"CLIC6",1.06383214459044,0.65778388108964,10.0546904146646,3.60622456751477e-21,4.51770469740737e-20,37.2707979033087

"KCNQ1",1.06348841934664,2.01253260752985,8.87758452680623,3.0683386165151e-17,2.34726317638785e-16,28.3503364055204

"BATF",1.06335765635065,2.82972578164516,6.65173506098934,1.05361723603855e-10,3.3902277092628e-10,13.5857458303079

"GABBR1",1.06329301878826,1.0268322210959,12.6827748323025,8.10473021321859e-31,3.830973913884e-29,59.230224374023

"SLC39A6",1.06321762740117,4.15736596759447,11.1705648139634,3.88171634475962e-25,8.06601029785374e-24,46.2947162337123

"MCUB",1.06307774646165,1.69045627766814,10.5384002575912,7.30172974614429e-23,1.1407507032123e-21,41.120737215475

"HLA-DPB1",1.06239551418196,6.3547110825706,7.6125383048524,2.29450269975651e-13,1.04807556168254e-12,19.5839314951157

"PDGFA",1.06177725059336,3.61303261574076,8.1217254756618,7.062097958031e-15,3.97881718541769e-14,23.0008675314162

"ELF3",1.06176504292922,4.62695307951835,7.73897817652476,9.80734387595749e-14,4.7156208204352e-13,20.4176847334376

"ABCC4",1.06125115106657,1.9778986132417,8.4402441320389,7.41561649653218e-16,4.76810282773549e-15,25.2162070054224

"TNFSF13B",1.0608865520238,1.8311642237352,11.6781322231191,5.2172714509722e-27,1.40856808607908e-25,50.5549738053366

"SLCO3A1",1.06073548458123,1.92233866515951,11.3091847850794,1.20701826255697e-25,2.69348947127155e-24,47.4492878236266

"SLC41A1",1.06064229390559,2.46086498162833,9.49489523168178,2.89522192189023e-19,2.90272132031342e-18,32.9448539414691

"TWSG1",1.06006625821822,2.61197582914424,11.4913640290116,2.57371524548283e-26,6.40102665075061e-25,48.9769945265529

"RHOQ",1.05928511528583,3.3757468414433,12.7649341989973,3.90635585219799e-31,1.93292758639696e-29,59.9524609914799

"CD37",1.05918811373999,2.32547205746747,10.7844242070362,9.68618548714908e-24,1.68596605038083e-22,43.1159527769214

"MYLIP",1.05846407912824,2.64763300821542,12.0577964214664,1.96528425486267e-28,6.41862705313316e-27,53.7977537370463

"EVI2B",1.05735300547033,2.58358885969745,9.71450837637332,5.27078416030918e-20,5.77211337170794e-19,34.6246491976954

"SPINDOC",1.05729461067364,2.94696950924842,11.0184954664755,1.38709375169523e-24,2.69672168939959e-23,45.0361699549426

"GMIP",1.05716606558657,2.73590134688917,13.0888239521883,2.16234863837969e-32,1.38311432665364e-30,62.8166247372866

"CAV1",1.05672900514034,4.23796193140125,10.8323675237812,6.5163632928383e-24,1.16016359708234e-22,43.507546911791

"B3GNT5",1.05672809324596,1.68236441020174,8.5427767370107,3.54698572190028e-16,2.37455446857532e-15,25.9415809627726

"ZSWIM4",1.05638314825344,1.97778762963881,13.7914235435306,3.73040892382719e-35,3.91428368993073e-33,69.1150398302352

"ACTA2",1.05621979318677,6.03203334093316,9.37082183046793,7.50365490821482e-19,7.12558243690875e-18,32.0060710727467

"FHOD1",1.05614760511841,2.2397249458191,14.4717352463443,7.1542285517784e-38,1.18929001599507e-35,75.3102468611146

"UBE2Q2",1.05542965054781,3.36764631809908,9.62411010664505,1.06556680587091e-19,1.12607577806143e-18,33.9304347722727

"ZC3H12A",1.05538524715358,3.33047241513397,10.0482465588879,3.79598203463487e-21,4.73227337036621e-20,37.2201895906158

"ATP2A3",1.05534828889992,2.12680474572524,10.5870374014138,4.9070236890324e-23,7.88267269047062e-22,41.5132548634844

"S100A3",1.05526169441947,0.947310767265741,12.5764578028861,2.07849873134712e-30,9.26246648502429e-29,58.2983097134377

"ARMCX1",1.05484452463245,1.96888679746013,10.2822571620414,5.82857919505162e-22,8.13526690479138e-21,39.0696663083424

"MTMR2",1.05454953054823,2.35213774860301,13.6072840950926,1.99747086496821e-34,1.87041654729143e-32,67.453791377578

"PLEKHA2",1.05437716993145,2.29824005408904,12.1737571123601,7.15559490010198e-29,2.52665934479735e-27,54.7971423278717

"PLXDC1",1.05429578048787,1.3762300180571,14.3234553581088,2.81792230082021e-37,3.9705867086319e-35,73.952703609516

"TAGLN2",1.05417705492052,8.53179320351049,12.5983242625556,1.71291270481573e-30,7.77378633980023e-29,58.4897282400316

"RGS4",1.05342356168031,1.01404374137591,9.55365176321268,1.8395436640817e-19,1.88869177724419e-18,33.3920274000247

"TMEM165",1.05333960224078,3.29048856180227,14.4521870646078,8.57325150473339e-38,1.39385995618165e-35,75.1310608779842

"ZNF385A",1.05322044094596,3.23809409986999,10.757843140213,1.20623162209653e-23,2.07032446043133e-22,42.899228335834

"COL15A1",1.05307338116962,2.94755025815952,8.38414453364515,1.10745775380189e-15,6.9663424606713e-15,24.8218213048065

"PTPRE",1.05280105054554,1.27923208395998,14.1323528374512,1.63961456945256e-36,2.00480145083063e-34,72.2088737251704

"IGF1R",1.05236280101506,0.898801077545734,12.3159064395673,2.06263961109194e-29,7.90589457152985e-28,56.0276996417843

"SULT1C2",1.05215684169391,1.96164595744369,6.39017630611752,5.0278023100972e-10,1.47008567545233e-09,12.0615528545548

"HLA-DOA",1.05197423525409,2.49011756895006,8.18862089763017,4.42010020255613e-15,2.57563538782268e-14,23.4612837344518

"CTTNBP2NL",1.05025004593075,1.89714622743576,14.6634818220232,1.20861613613412e-38,2.55449653344347e-36,77.0712478449278

"LPAR1",1.04941021733742,0.79934362859805,12.1146751746996,1.19790376417081e-28,4.07424969905912e-27,54.2874464421639

"HLA-DMB",1.04897828264071,3.15986787523947,8.49818220970289,4.89180454496035e-16,3.21663325523059e-15,25.6253733377245

"JAG2",1.04866025870331,2.32076042562143,11.6466461179527,6.83349688463214e-27,1.81185638724252e-25,50.2881334238864

"CACNA1H",1.04790870111754,2.18749818723986,5.33157505188101,1.70488035640952e-07,3.61785784180706e-07,6.40696148623041

"ADAMTS9",1.04789386303528,1.78042943325654,12.22059538637,4.75252860957033e-29,1.71079466614582e-27,55.2019540387943

"RASSF2",1.04780283630092,1.96873946667234,12.6776705806407,8.4801793616694e-31,3.99567686802226e-29,59.1854137530745

"EPHB3",1.04758761348182,0.910392582826494,10.9797405689544,1.91632969130141e-24,3.64891863356556e-23,44.7168027610679

"BAK1",1.04466984446586,4.04552656081113,10.6848924639624,2.19940966076589e-23,3.69775749216266e-22,42.3058636080196

"FGR",1.04442875430593,1.96654575148622,12.7988715886812,2.8881497582135e-31,1.48885629521842e-29,60.2513127774287

"IGFBP7",1.04414278851361,7.87039293647852,9.51431997416524,2.49253902634878e-19,2.50864727175716e-18,33.0925045885136

"KCNS3",1.04346227453738,1.83586103655357,8.18453086912318,4.54889769490508e-15,2.64236126408012e-14,23.4330587425198

"TNFRSF12A",1.04257507516214,5.85066457033421,8.00137125438241,1.6302222694783e-14,8.76422183028032e-14,22.179133868443

"FCGR3A",1.04074882750576,4.15954232027039,7.18758437965825,3.71440793333234e-12,1.44427504267154e-11,16.855855548275

"RAB11FIP5",1.04053660215807,2.92556753425156,11.4121198567314,5.04785720981292e-26,1.18922050030545e-24,48.3110543375005

"ITGAX",1.04045979642845,2.21278544768856,11.6596496044009,6.11304623659547e-27,1.64142502850145e-25,50.3982963750342

"SLFN11",1.04027362371423,1.75531642167782,11.7249783168553,3.48987846317012e-27,9.63297609376902e-26,50.9525929817296

"FRAS1",1.03995410846998,0.728618505530759,9.19846906283277,2.78243220612734e-18,2.45914483211792e-17,30.7145836588925

"UCP2",1.03988751171156,4.11535344810575,8.94245026345296,1.8967391301517e-17,1.49985331002642e-16,28.8239510724025

"PLEKHO1",1.03966512885754,3.11721089453731,11.0524684282611,1.04439021684202e-24,2.05475442263002e-23,45.3165928869397

"OSBPL3",1.03915123828058,1.77757699358196,12.8024097929707,2.79858665850757e-31,1.44773040603565e-29,60.282487426595

"SEPTIN5",1.03848685324416,1.8556374672277,9.5926847389803,1.35977848656502e-19,1.41875336450842e-18,33.6900061639435

"MZB1",1.03794679287738,1.53305607052692,6.09591102572444,2.75541370239142e-09,7.34528751835694e-09,10.4054455839894

"PLEKHO2",1.03768990458215,3.74473594930277,12.7791573978042,3.44208499854887e-31,1.73807670831162e-29,60.0776734416219

"EZR",1.03738586301507,5.72121115842715,9.21852992176365,2.39058660092275e-18,2.12936356174907e-17,30.8641416987244

"BLMH",1.03668152731161,3.36992351256187,7.89988855945653,3.27903811293608e-14,1.68566257404063e-13,21.4929430645998

"UBE2C",1.03640195278599,4.29770291990286,5.97657845441065,5.39714183106205e-09,1.38221764567359e-08,9.75196133580007

"CARD11",1.03596884608303,1.33083156489968,11.5027651609036,2.3355792602751e-26,5.83697553306926e-25,49.0729832870104

"HCLS1",1.0358045931358,3.43460541489379,10.5429364034602,7.0363086614195e-23,1.1039468361156e-21,41.1573047831882

"PTPRC",1.03524916798095,2.11405491387772,8.5953384170673,2.42496359128633e-16,1.65638671897882e-15,26.3156956881835

"CCDC9B",1.0349741318597,1.7383945970427,9.43727521810781,4.5097528093612e-19,4.40407873363029e-18,32.5079481351832

"ARHGDIB",1.03431915384452,5.93120352409994,11.6475160766982,6.78276357889889e-27,1.80163352154056e-25,50.2955017900237

"ACSS1",1.03388695596104,2.25906506316603,8.69820972644206,1.14710756222528e-16,8.13978723411179e-16,27.0522834401402

"UNC13D",1.03351721158016,1.63304855686764,10.5636532193309,5.94096742347619e-23,9.45124871293873e-22,41.3244171317183

"FABP5",1.03305755740704,2.77189900733244,9.38987902517544,6.48567912747631e-19,6.22280302795149e-18,32.149775893886

"SLPI",1.0328253479591,7.38107292576795,3.23246087598453,0.00133835285716205,0.00187313693328092,-2.10966132386265

"HAND2",1.03244163659367,0.846457155663574,10.8508969644226,5.58946719529461e-24,1.0023777836895e-22,43.6591313490381

"CMTM7",1.03241715075301,2.64095845520141,11.4340101334982,4.19167370882673e-26,1.00187096158468e-24,48.4947957631487

"VASH1",1.03206754232637,2.15044890442067,13.0560186295951,2.90229053779645e-32,1.80067950687189e-30,62.5253304042404

"PTGER4",1.03167390476648,1.45261376417859,12.0109881368844,2.95150579861948e-28,9.43143159623656e-27,53.3955033763932

"ITGA5",1.03166132302832,5.02027306184106,10.3373257841994,3.73767170872381e-22,5.35322874448875e-21,39.5083149883523

"ACKR3",1.03160394275102,3.83422180547898,6.91017433541216,2.14921741719018e-11,7.55468559927032e-11,15.1386251217419

"NEXN",1.0303127744941,1.39081069265892,12.3591542763338,1.41111453403793e-29,5.59816564917092e-28,56.4032567314882

"SELENOW",1.03002591272392,4.72273661942338,8.18634442999222,4.49133609353709e-15,2.6126670894394e-14,23.445572795705

"MGAT5",1.0298406345129,2.77331827703871,11.4842867737611,2.73353965832184e-26,6.75170605089677e-25,48.9174318581007

"EPB41L2",1.02906751919249,2.82035203964617,10.5792407339122,5.23016320185272e-23,8.35640006170745e-22,41.4502687007787

"MS4A6A",1.02870298821566,2.93401838192173,9.46130530599075,3.74944889227912e-19,3.70809467655546e-18,32.6899614462975

"TMEM158",1.02831380525548,0.987077953764719,11.505210499419,2.28743257373918e-26,5.73602795397816e-25,49.0935769420676

"CALHM2",1.02800169097797,2.28267845636549,12.9069959004412,1.10132050627158e-31,5.94672879207592e-30,61.2054424730406

"RNF24",1.02798021957688,1.7884636158441,14.6611879829079,1.2346457751845e-38,2.57275834420489e-36,77.0501457117414

"TNFAIP8L3",1.0278043994167,1.89365564032178,9.08267635430234,6.656083937646e-18,5.66283851969365e-17,29.8553120698971

"GPD1L",1.02731655292272,2.2067928950691,10.2951819466581,5.25198380058753e-22,7.35824813728148e-21,39.1725031616752

"FABP3",1.02724347373823,2.96061175420483,7.86047736339272,4.29439644723381e-14,2.16771052326251e-13,21.2281280262539

"CYP1B1",1.02710333086584,2.63597845664959,6.72598794018363,6.70368825384951e-11,2.21040935403841e-10,14.0271953840005

"PLEK2",1.02696749991648,2.96535121510227,5.27077668876663,2.3229404034582e-07,4.84191367556552e-07,6.10836279029484

"SIPA1L2",1.02627066676842,3.05413231681541,7.66200042696027,1.6473540454668e-13,7.67641042604135e-13,19.9089069817078

"DOK1",1.02614659038328,2.00571510972227,13.4584766780276,7.71068438618802e-34,6.37316064210345e-32,66.1165992398116

"FUT8",1.02535419374808,1.23603050756656,14.5580794713672,3.21462094637697e-38,5.66194248829134e-36,76.1024811236519

"TP53I11",1.02502465077491,3.3375550701279,11.1697168644114,3.9094702324674e-25,8.1009260629349e-24,46.2876749497483

"RAI2",1.02488814248089,1.88454573680561,9.7061348548853,5.62679737215442e-20,6.13121643138862e-19,34.5601836358098

"MLLT3",1.02467845149885,0.948524469017631,12.3574870658711,1.43193061294201e-29,5.66454904237353e-28,56.3887689699535

"ARHGAP4",1.02446930421317,3.47628134737278,8.54561290058767,3.47507769012806e-16,2.32852239245673e-15,25.9617287622993

"PYGB",1.02381635498908,5.28665595802774,10.3934200741118,2.37401485295928e-22,3.47757918312203e-21,39.9564476316935

"SLC52A2",1.02358222917483,4.47994526421855,10.5996952592713,4.42414826324572e-23,7.13020409092815e-22,41.6155649078485

"LGALS2",1.02355278995871,2.56363803945793,6.52174959051959,2.30421719681836e-10,7.06107983159231e-10,12.8222293612252

"TNFRSF18",1.02311096649582,1.64542863889705,9.80581384489245,2.5789850755904e-20,2.9328273784289e-19,35.32969968753

"CEMIP",1.0229653508098,1.12244891239217,9.32408117875397,1.07212415046174e-18,1.00265972225546e-17,31.6543705973705

"PALD1",1.02242437246024,2.15900052610472,13.327801960461,2.51462510387816e-33,1.9078912006091e-31,64.9464114503451

"BICDL1",1.02208511481058,2.44789139744896,8.22344105316064,3.45993529971831e-15,2.04268726094702e-14,23.7019681726674

"SLC35F2",1.02208271428619,0.946851829277042,10.8800592892138,4.38917798086505e-24,8.01702323788869e-23,43.8979685074774

"KCNF1",1.02183274082656,0.848729420364094,8.91424884531381,2.33853739928078e-17,1.81811144626164e-16,28.6177674744963

"MMP1",1.02158972472368,1.16191482610929,7.97544411393067,1.95000211063554e-14,1.03517334864918e-13,22.0032373128501

"AP1M2",1.02095393345179,2.56962505980426,3.98400818759771,8.17373511453817e-05,0.000129212961875833,0.501957252165001

"C1orf198",1.01963138257961,4.60084423801776,10.5770891714799,5.32300231997636e-23,8.48518165588492e-22,41.4328914137696

"TACC1",1.01936215414655,3.50546125765255,8.95913986320908,1.67535768633087e-17,1.33336763664825e-16,28.9461668752858

"ZNF239",1.01914051378441,1.14365946763833,10.2297789904242,8.89065538707118e-22,1.20345147714289e-20,38.6528521163121

"MEDAG",1.01911754414954,0.782000374990774,15.1332981338805,1.51208478946189e-40,4.86332488262798e-38,81.4102622265922

"SH3BP1",1.01907272714446,1.82855611262474,10.1889661523189,1.23365632858207e-21,1.63401480585244e-20,38.3295076905367

"MAP7D1",1.0183930904444,4.10953260406716,14.4355090760345,1.00038688232454e-37,1.55797093936753e-35,74.9782356991053

"CCL26",1.01789892478087,0.775968621845126,10.5908117000994,4.75780305396407e-23,7.65127132428243e-22,41.5437547357576

"PMAIP1",1.01758046135472,0.848014145101623,12.4312923011055,7.48271428443656e-30,3.0837537002295e-28,57.0308835339835

"EDN1",1.01751681419953,1.82285200568302,9.74153324990061,4.26742904408227e-20,4.73643006055493e-19,34.8329295602658

"CRIM1",1.01750494111347,3.55258697421685,6.96012936457773,1.57254399217834e-11,5.60486349416492e-11,15.444052261115

"CCN4",1.01719504275034,0.923567292998875,12.344776808456,1.60101349232789e-29,6.23341963657661e-28,56.2783453554027

"CYTIP",1.01688605421987,1.71995839930149,10.1504004737507,1.68011458802062e-21,2.19007007310706e-20,38.0246279036827

"C6orf132",1.0167229143872,0.779448021820586,8.48293779478872,5.45865741358543e-16,3.56245418764872e-15,25.5175332658861

"B3GNT7",1.0161961672307,1.1711956473921,8.84894277518937,3.79175954026628e-17,2.85928044843219e-16,28.1419165802722

"DNM1",1.01520732391575,1.17839501242407,9.37961240063853,7.01577586049164e-19,6.70099443873298e-18,32.0723360707382

"ZG16B",1.01516951689495,0.774792988930705,8.55617802997768,3.21968159808165e-16,2.16917983805182e-15,26.0368214942653

"PLXNA3",1.01487967267625,1.90252895061559,10.4918902315872,1.06683871569604e-22,1.63563510867596e-21,40.7462871342433

"SPON1",1.014871624,1.04696988435464,9.97437913097482,6.82438265342965e-21,8.26241745969654e-20,36.6413710694285

"LCP2",1.01422325670305,2.14951747543892,11.471192468247,3.05573791141339e-26,7.46033702959753e-25,48.8072745661009

"CYP2S1",1.01353008758672,1.39927982753986,8.58814662452727,2.55470584111267e-16,1.74098907965278e-15,26.2644171630231

"A4GALT",1.01350455840022,2.44263522772457,10.1270324595128,2.02528638775299e-21,2.62382768010555e-20,37.8402071813862

"NOTCH1",1.01331340552664,2.86920579132731,10.81933526029,7.25834089873955e-24,1.28607369577068e-22,43.4010129439582

"ELK3",1.01284611807254,3.39518579403166,11.9490471822557,5.05026157413584e-28,1.5664280920197e-26,52.8642502194458

"ARHGEF16",1.01263978210685,2.49670390835391,7.11453423562951,5.92589961130282e-12,2.23828656495341e-11,16.3986876374711

"LIMA1",1.01262591102025,3.34426313287026,11.2924743618371,1.39003442874077e-25,3.07407464472642e-24,47.3097414846816

"TOP2A",1.01242357758513,3.64818932580216,6.04891054346495,3.59514418310196e-09,9.41418729008734e-09,10.1468018399155

"FMNL3",1.01237030650945,1.83520951259062,15.6747117915308,9.34567081882445e-43,5.53076799058031e-40,86.4480559719209

"ANLN",1.01226616980228,2.2494934996303,7.692323634387,1.3435304348504e-13,6.3324411543841e-13,20.1088881475588

"NBEAL2",1.0116330333635,2.47063210568557,12.7870366499261,3.20900880601046e-31,1.63714776844568e-29,60.1470604159535

"ANXA3",1.01108380650709,0.74754248289339,9.46416851424459,3.66779707267205e-19,3.63219930991854e-18,32.7116672056766

"CD300A",1.01105088652178,2.2831503438911,10.8188841009318,7.2854791564473e-24,1.28933808755548e-22,43.3973260602149

"SMIM10",1.01057730490812,1.53831709923189,11.3967408765218,5.75140282376662e-26,1.34026147438408e-24,48.1820666419573

"NCF4",1.01022062224736,2.84624479883605,10.1608055695914,1.54587213476934e-21,2.02220850874557e-20,38.1068215348706

"CCND2",1.0097074632346,1.84891385537048,11.0049631282127,1.55290664703402e-24,2.99546986217319e-23,44.9245900824668

"ALDH3B1",1.00933826666875,2.48450487531768,10.0814018980476,2.91506653907366e-21,3.6988344292963e-20,37.4807781040449

"CLGN",1.00930283605397,2.69427810553791,5.38869962144239,1.27160191737136e-07,2.73667322017256e-07,6.69018469011583

"GNA15",1.00868597854623,1.66688975977466,13.0296182136672,3.67721355924437e-32,2.18491464293255e-30,62.2911001377473

"C1QB",1.00761392038277,6.86635078961002,6.74027625045492,6.14244947391724e-11,2.03214534809046e-10,14.112581084393

"RHBDF2",1.00735221637329,3.04226596739076,10.6824260064351,2.24445323998888e-23,3.7649303498453e-22,42.2858387591431

"MTHFD1L",1.00641422808176,2.64732443689968,11.5018285506126,2.35428634219482e-26,5.87380546927021e-25,49.065096073666

"NRP1",1.00586393081912,4.05688580164553,9.72475447024254,4.86544092552836e-20,5.35198501808119e-19,34.703575697403

"BCAT1",1.00564327245889,1.08249798788186,12.688648817015,7.69312215559338e-31,3.64806866320526e-29,59.2818011631519

"GPBAR1",1.00475898327312,1.16336171640094,12.0909795657993,1.47245958142884e-28,4.90654042955851e-27,54.0833216755663

"ADCY3",1.00473502896282,1.67079734200448,13.0480281971587,3.1178540653579e-32,1.92202712070709e-30,62.4544194308071

"MSR1",1.00466057875477,1.91426200102472,11.0327664987687,1.23128205368354e-24,2.40644887506577e-23,45.1539148729851

"STAB1",1.00366195958736,3.52258257947478,11.5412543692305,1.68230948331893e-26,4.29875108906799e-25,49.3973601797624

"SPART",1.00362101790737,1.71404970761372,9.85209612346549,1.7925374359058e-20,2.08004638150795e-19,35.6885536049684

"PTPRM",1.00321916913528,2.98097763984194,9.37596176554842,7.21444545883711e-19,6.86416209411544e-18,32.0448122747323

"RGS19",1.00286477065631,3.3551811977821,11.3075666878478,1.22363702977676e-25,2.72646232764265e-24,47.4357709942053

"CSF2RA",1.00193259246727,1.48668930865439,10.886069092234,4.17570612275673e-24,7.64598664432993e-23,43.9472289124182

"PPP1R15A",1.00167487229201,4.75993584454969,11.4291811183296,4.36717314224598e-26,1.04045614556408e-24,48.4542479541548

"FJX1",1.00151007330262,2.00379776724916,11.316954602327,1.13029387924687e-25,2.53758694134407e-24,47.5142061726792

"APBB1IP",1.00109464165813,3.54051714711486,7.20158867865658,3.39491353640517e-12,1.32702102433592e-11,16.9438998039352

"MAPRE1",1.00099670972383,5.36765896888033,11.6340487664253,7.61191853755915e-27,2.00388496019907e-25,50.181464722229

"ITGBL1",1.00089151214919,1.08847875652397,9.29168516128385,1.37210007641289e-18,1.25931889767548e-17,31.4112408934259

"ASPHD1",1.00039792242568,1.94266848638758,5.50443166916326,6.96729789922873e-08,1.54475007371631e-07,7.27185216881485

"AR",-1.00147984433116,3.47717298473861,-6.3875695438278,5.10550849593462e-10,1.49103825892919e-09,12.046606938896

"LDHD",-1.00440786971333,6.00782564291025,-7.86960513424902,4.03463253507087e-14,2.04776632440389e-13,21.2893765288773

"GLYCTK",-1.00661025262541,6.79949306823849,-9.72982413453612,4.67647942799775e-20,5.15947152402883e-19,34.7426458213725

"FABP1",-1.00666861377521,8.9297866789744,-3.72455764783892,0.000226408156180686,0.000341702404436728,-0.456758168179729

"F11",-1.00858850388154,5.03306714087375,-7.49892064201974,4.88400767004187e-13,2.12964613846948e-12,18.8432464364494

"HGD",-1.01216990803376,7.76742608756773,-6.99569479944929,1.25768843889509e-11,4.53730808423624e-11,15.662526871007

"RORC",-1.01446489434404,5.26921160770676,-7.04007200158848,9.50602375568942e-12,3.48467843075879e-11,15.9363222017992

"CPB2",-1.01773095254172,9.06474581348998,-7.20886226628298,3.23981090582918e-12,1.27029358077599e-11,16.9896792334897

"SHD",-1.02398256235236,2.20684241060822,-6.90598266579768,2.2061365700815e-11,7.73578342766791e-11,15.1130738982894

"CFHR4",-1.02508748459357,3.99910002178637,-4.81871009854601,2.11828815928096e-06,3.93373582484773e-06,3.98142272867301

"SRD5A2",-1.03043964177666,2.41332009771786,-5.48680166199693,7.64101750591218e-08,1.6845306809711e-07,7.18256679882601

"SAA4",-1.03916581447591,7.2560371913496,-4.49184142139401,9.47206704762777e-06,1.6486968467018e-05,2.54831530516147

"SELENBP1",-1.04171997631603,7.05571048013561,-8.19568018361071,4.20622487073683e-15,2.45778424022715e-14,23.5100222191943

"NR1I2",-1.0444265430476,3.27686135264482,-6.04217700909319,3.73431770484281e-09,9.7579001135905e-09,10.1098811713753

"EPHX1",-1.04519124040814,10.9042861509353,-7.17510716960421,4.02388088504785e-12,1.5531781292534e-11,16.7775205798868

"LEAP2",-1.04520930459633,6.12179763549161,-6.69052623066999,8.32334123245444e-11,2.71122486865177e-10,13.8158900526883

"ABCG5",-1.04664212193966,4.50673836953968,-7.2107204364535,3.20131926843854e-12,1.25632675269359e-11,17.0013799839543

"LIPC",-1.0466435282566,5.22292875338432,-7.35771370835448,1.23506886038254e-12,5.09276582758072e-12,17.9341152512526

"F7",-1.04756314481581,6.36590795192032,-7.6443912229344,1.85392465056233e-13,8.59029602413705e-13,19.7930365068401

"FXYD1",-1.05077167667724,3.34177202775309,-4.86525618596813,1.6996285876032e-06,3.19192751378387e-06,4.19272632618945

"GPLD1",-1.05367176187559,3.20067133847149,-5.6452420341643,3.30564810539429e-08,7.65249002023291e-08,7.99364173216759

"THRSP",-1.05842173127968,4.12886471474806,-3.15889518801817,0.00171498983660986,0.00237911623372178,-2.3378456109439

"APOC2",-1.05931365386189,6.53707063956371,-6.91088097196857,2.13976517125201e-11,7.52503582331197e-11,15.1429337504912

"MLXIPL",-1.06474895611964,7.01331467261127,-9.82537183154653,2.21177266226375e-20,2.5406192964435e-19,35.48122460275

"SORD",-1.06567519701902,5.84507446178731,-8.1541030933693,5.63088858064565e-15,3.21904932575936e-14,23.2233844797961

"ABCB11",-1.06946157168208,3.13111943525434,-5.38421686540391,1.30131723234453e-07,2.79555516952771e-07,6.66786607312832

"ABCG8",-1.07001486599503,5.26119827360859,-5.99539777513855,4.85750890222519e-09,1.2515995159948e-08,9.85431577489271

"CYP2C8",-1.07085923168366,6.75353111215477,-4.06295370259715,5.9286999768651e-05,9.51563421107823e-05,0.805427932851416

"AMBP",-1.07251101956876,12.3793588993793,-7.53260150015733,3.90730815045855e-13,1.72563056973236e-12,19.0619675222393

"DPYS",-1.07284003822875,6.49471539018785,-5.34175289020099,1.61839453145249e-07,3.44123988112095e-07,6.45723430342403

"F10",-1.07314647420577,7.52625821864418,-8.64847267145954,1.64851937722777e-16,1.14613929445887e-15,26.695430375068

"CTH",-1.074034349351,5.03773613252116,-5.5003839864657,7.11667161456934e-08,1.57597899322786e-07,7.25133163686419

"ADH1C",-1.07845024239686,7.65548974856429,-3.4987849648693,0.000524786478583744,0.000763443062993755,-1.24226605880833

"MTTP",-1.08114167765835,5.67109567037834,-6.16189564643337,1.89162611268218e-09,5.14932996083402e-09,10.7713090654355

"TFR2",-1.08559610975334,8.04546973293264,-7.25998108161988,2.33009490452876e-12,9.31723084121703e-12,17.3123940831281

"PAH",-1.08563378832142,7.43395526751532,-6.55374831781964,1.90252267312522e-10,5.89483203118066e-10,13.009083244932

"SPDYC",-1.0866733476518,2.09710698486288,-9.2740871698719,1.56852998704981e-18,1.43111129781493e-17,31.2793880513852

"SLCO1B1",-1.08743521631047,6.35446376954705,-5.54594926173173,5.60094288856313e-08,1.25497425467653e-07,7.48307088559325

"RHBG",-1.09009542546606,2.75566445631724,-3.89438879467341,0.000116954092432937,0.000181987357756133,0.164077825633264

"IL27",-1.09253677116169,3.11120222963487,-8.4497493432082,6.92724322438297e-16,4.4715778143432e-15,25.2832051241746

"HAAO",-1.09353566798995,6.51589359002478,-9.54781324169977,1.92448466555459e-19,1.97179713482549e-18,33.3475185174404

"MST1",-1.0963223214705,6.45469527622352,-8.94982338562388,1.7955740768508e-17,1.42366122545592e-16,28.877925531048

"SARDH",-1.09817659617032,5.29355512353881,-9.65087423969382,8.65449631871996e-20,9.18531370412208e-19,34.1355697801911

"CYP1A2",-1.09891279738904,2.71109300948106,-3.51635170313287,0.000492318143852749,0.000718682480345478,-1.18279112087791

"UGT1A4",-1.11191376263141,4.14817610968493,-3.95976558236012,9.01147712492373e-05,0.000141849988363918,0.409862453941772

"PROZ",-1.11625702595055,4.29201482936744,-6.72018620600035,6.94568844282436e-11,2.28612815376166e-10,13.9925650331587

"SLC6A12",-1.11761036044351,3.89866415030397,-6.99758629130123,1.24280391014468e-11,4.48579747513797e-11,15.6741699232873

"FMO3",-1.12136459482478,7.07134799593196,-5.20112031896996,3.29945563434763e-07,6.72945217951106e-07,5.76987755931371

"HLF",-1.1234722964936,4.37045337689893,-6.6801806200245,8.86435417507372e-11,2.87731724484896e-10,13.754408183553

"SLC47A1",-1.13141986434413,5.00062937390204,-8.27246997104521,2.44790934028045e-15,1.47860956413452e-14,24.042054696846

"BAAT",-1.1319684105505,8.34836482263898,-7.11943525536194,5.74368480814708e-12,2.17445795129314e-11,16.4292492264312

"CYP27A1",-1.13238610404869,8.42239383029614,-8.67390796114496,1.36970209674588e-16,9.62238486294171e-16,26.8777551332419

"PKLR",-1.13716309585427,6.35229924024789,-5.75742390754036,1.80589343146395e-08,4.31008119350042e-08,8.5796224284216

"GPT",-1.13750900667454,6.00559253811597,-7.60630021841144,2.39216549208639e-13,1.08932251324771e-12,19.5430544640339

"CYP17A1",-1.13798660994625,3.2297208597327,-4.07034824663689,5.7515105722187e-05,9.24829898010822e-05,0.834131751577006

"GSTA2",-1.1387419420745,6.02712610707508,-3.49045915351969,0.000540865138273988,0.000786138100084846,-1.27035710329936

"CYP4F3",-1.14021414059681,5.91502214068608,-8.35271234629326,1.38544012145961e-15,8.57639606568826e-15,24.6016278479621

"CLDN14",-1.14686600348965,3.92957690606628,-9.03987999911145,9.17230900844038e-18,7.63241348593225e-17,29.5394657371753

"PRODH2",-1.14888069104745,5.58009456887058,-6.60050357652315,1.4362270984405e-10,4.53941036561146e-10,13.283403329666

"KHK",-1.15002534091451,6.91071759512861,-8.93965390101776,1.9365670701558e-17,1.53052936981598e-16,28.8034879309613

"RGN",-1.15070247039188,6.55302684017009,-8.75620934161045,7.50290891743809e-17,5.42540932203066e-16,27.4701122879146

"ABCB4",-1.15410367937523,4.68891327199451,-6.58512447283258,1.57564734787137e-10,4.94835544719953e-10,13.1930022839647

"BDH1",-1.15529481528479,5.11031319823251,-9.04764398103215,8.65454950259951e-18,7.23412767745535e-17,29.5966955249633

"CES2",-1.1558867948622,7.47834516708364,-7.34715361850649,1.32313797425451e-12,5.44529244202933e-12,17.8666399665921

"ACOT12",-1.15937472107589,4.44850112327019,-6.52444857793046,2.26734756886193e-10,6.9596280666623e-10,12.8379619496724

"PAGE4",-1.16067631630482,1.53990326011923,-4.3661927419859,1.64644947094845e-05,2.79509121315918e-05,2.02137281573402

"SERPINA11",-1.16739472120494,6.91722983258164,-5.26770939495737,2.35930017924143e-07,4.91562401800831e-07,6.09337624883388

"C8A",-1.16914570821121,7.13915182323008,-6.57794173423622,1.64523137930122e-10,5.14286884782624e-10,13.1508377901929

"SLC22A7",-1.17138543456847,6.43180242290785,-4.42972824770292,1.2469022519805e-05,2.14342130375184e-05,2.28614870215073

"PROC",-1.17450569133675,7.94877268616335,-9.297255464345,1.3151625045427e-18,1.21232581026225e-17,31.453008489288

"ANGPTL3",-1.17890980653073,7.40007145194048,-6.8017158454501,4.21097989426768e-11,1.42729547618993e-10,14.4813430643153

"FNDC5",-1.18195003838709,2.96047265764296,-4.96949539638603,1.03157321458978e-06,1.97978021920557e-06,4.67239454588545

"CYP4F12",-1.18502064995517,3.63596625495433,-9.91585306946916,1.08431573855672e-20,1.28032333215855e-19,36.1844984999056

"F13B",-1.18503431069142,6.30055041329221,-6.70576864085388,7.58483280196275e-11,2.48323968366981e-10,13.9066080227256

"TM7SF2",-1.18563375346072,6.59220093630259,-9.27268584739272,1.58532036410566e-18,1.44337321765805e-17,31.2688952888983

"SLC6A1",-1.1883206205229,5.35507563987532,-6.76939141992377,5.13785095543517e-11,1.72173283999237e-10,14.2870070529014

"DMGDH",-1.19422663604845,4.79765325755102,-7.9240839572023,2.77731243857036e-14,1.44328547694586e-13,21.6559831329369

"SLC25A47",-1.20094882737345,5.02174386071682,-3.84655989407443,0.000141210258078398,0.000217875249584931,-0.0133496295194231

"HSD11B1",-1.20134181312962,6.9342064511805,-3.71362145817165,0.000236051065019553,0.000355530439475138,-0.4958588376975

"ARG1",-1.22034175746943,7.79524683710777,-5.1372349639267,4.537245983849e-07,9.08616057539876e-07,5.46285091595872

"EHHADH",-1.22251514655724,6.24649999493917,-8.35328682002644,1.37978782426831e-15,8.54855982414141e-15,24.6056472055132

"ACOX2",-1.22525373719269,5.6460898510058,-9.68572080850531,6.59790035216785e-20,7.13045549381471e-19,34.4031585359047

"NAGS",-1.22551388183642,4.33083068298782,-7.57208174539726,3.0054379713485e-13,1.34825514815346e-12,19.3192604840349

"CYP2B6",-1.22815962372716,4.91001168693812,-5.65913611130375,3.06871913626807e-08,7.13863201589405e-08,8.06569334829787

"MAT1A",-1.23088514023707,8.61191206686154,-7.90033597462285,3.26899433362701e-14,1.68108346075814e-13,21.4959547600346

"ITIH3",-1.23135685974346,8.94429760878765,-7.34938372877093,1.30403832797455e-12,5.3726669625128e-12,17.8808836032636

"SLC13A5",-1.23211682621947,5.24744052715996,-5.65211681439051,3.18627058762307e-08,7.39464679904051e-08,8.02927425106383

"ANG",-1.23619094158934,8.82595185824757,-8.10475409150708,7.95032979252032e-15,4.44371474425153e-14,22.8844760343593

"SERPINF2",-1.23680160753334,9.75017994391723,-7.95174517199396,2.29608994410877e-14,1.20891995455834e-13,21.8428086160575

"A1BG",-1.24769441201223,5.19299763895918,-6.90914028180504,2.16312334328613e-11,7.59995484776021e-11,15.1323207184064

"SLC51A",-1.25372724930932,4.43826802254681,-6.52250823518802,2.29379470087881e-10,7.0349694443412e-10,12.8266510343124

"TMEM82",-1.26191080064684,3.63472542973607,-6.33423562999458,6.9801716672086e-10,2.00683326498933e-09,11.7418873800967

"CHAD",-1.26833175089876,4.21104812525545,-9.44259430119704,4.32926303482568e-19,4.24181765564543e-18,32.548212764105

"NOTUM",-1.27119572265181,3.50086398704255,-4.12668679959925,4.55763192842362e-05,7.39942259759273e-05,1.05438739216035

"MOGAT2",-1.27247160749772,2.97135512871231,-6.25721416467331,1.09267669224781e-09,3.06758096049455e-09,11.3054522309405

"F2",-1.27808564771946,9.95763331735402,-7.85955949022105,4.3214141748607e-14,2.18060445829004e-13,21.2219717735511

"AKR1C4",-1.28330757073753,7.29979965664784,-6.53731396224737,2.0994023769017e-10,6.46962261325986e-10,12.9130259281255

"SLC1A2",-1.28504516533043,2.68701223700036,-6.53021102389793,2.19055518851397e-10,6.73369291794394e-10,12.8715688848904

"ETNPPL",-1.28993878075987,4.70702389963489,-6.10625282665741,2.59820781605744e-09,6.93871563873103e-09,10.4625756769645

"GCGR",-1.29676591232251,3.18739719055937,-5.20695803937622,3.20429078305487e-07,6.54889931417278e-07,5.79809628653618

"APOC1",-1.29845286819557,12.5870336064071,-9.97807043777805,6.62762925544875e-21,8.03735859298067e-20,36.6702380961054

"HP",-1.2993003460167,10.6205565980727,-5.31057548551711,1.8977314421378e-07,4.00298498523364e-07,6.30349471884184

"APOM",-1.30025889294736,8.1883692581863,-7.44768156058622,6.84863852608594e-13,2.93187520235652e-12,18.5118828354319

"HPX",-1.30070630032561,9.86636474609666,-6.7212628931779,6.90013886415959e-11,2.27164117701916e-10,13.9989899811589

"CPN2",-1.3116919682432,7.03856847629418,-7.53780938269979,3.77457119725221e-13,1.66900122125961e-12,19.0958511090898

"RBP5",-1.31696776375737,6.83126302099869,-8.34429167446146,1.47096819286872e-15,9.08304441297689e-15,24.5427333923191

"PCK2",-1.31861719125895,7.4272972323415,-10.4146350964936,1.99887735921221e-22,2.96029935230677e-21,40.1262757403854

"SERPINA4",-1.33488361197705,7.29449503226509,-7.11806585267075,5.79403524875437e-12,2.19239773670897e-11,16.4207083562444

"CDHR5",-1.33550410205459,6.64663222541312,-7.1460049959757,4.84775888014495e-12,1.85186141574347e-11,16.5952084638894

"CYP1A1",-1.33639495527469,2.53953146810165,-4.83443726344126,1.96678132684905e-06,3.67127551485386e-06,4.05261894254945

"PON1",-1.33721188343502,7.15209963191393,-6.06236427579971,3.33208978684457e-09,8.77193387835684e-09,10.2206707345115

"PFKFB1",-1.34013615243077,3.70154125566521,-8.14082558032265,6.17935192233434e-15,3.51223633080817e-14,23.1320598939309

"ADH6",-1.34475849921845,6.32996947120205,-8.86111620512174,3.46567464363519e-17,2.62812180177256e-16,28.2304468812255

"CYP2E1",-1.34512712360301,6.78804091713766,-3.43498107435596,0.000660345226536625,0.000951295776690298,-1.45593856599762

"RDH16",-1.34924908508613,5.60980568538279,-5.73266241587343,2.06535618549926e-08,4.88911116231385e-08,8.44945244037198

"ALDH1L1",-1.35413280066615,5.55214661308428,-6.03067793197129,3.98425826422241e-09,1.03652366835186e-08,10.0469083388335

"F9",-1.36957237798481,6.24204746703261,-5.41589331999533,1.10497403027983e-07,2.39427222876247e-07,6.82591507461361

"HMGCS2",-1.37409495114819,9.55349026107272,-7.48125356669225,5.48885887863688e-13,2.37379909703106e-12,18.7288048971066

"GLYATL1",-1.37948150885374,4.46278206884822,-7.78418514934284,7.22028041744718e-14,3.53370985035167e-13,20.718177789697

"SEC14L2",-1.38816888621347,5.20762181747516,-8.43380600987067,7.76559791872546e-16,4.975834612713e-15,25.1708563007147

"SLC38A3",-1.40005137301482,7.97874080733925,-8.74444801990925,8.17862245594337e-17,5.89394638264404e-16,27.385237065161

"HAO1",-1.42133276509899,7.32712388408774,-7.89336486921194,3.42899338428873e-14,1.75908311791095e-13,21.4490437217351

"CYP4F2",-1.42274736441751,5.40145080838428,-6.91618372342982,2.07012561952071e-11,7.2887930844381e-11,15.1752774209963

"ETNK2",-1.42315567441384,5.65848996552766,-8.60320532970886,2.29050251187441e-16,1.57034219940602e-15,26.3718204990778

"SLC2A2",-1.4267770651522,7.44175879359694,-7.58102804732546,2.83154954893988e-13,1.27630319606696e-12,19.3776997816642

"SERPIND1",-1.43173869665618,9.29129777968674,-8.01248258451259,1.50958579522469e-14,8.15418833163537e-14,22.2546386290477

"OTC",-1.43543440990639,6.31858239001592,-6.13097516116693,2.25709605332687e-09,6.07269250936009e-09,10.5994660779068

"APOF",-1.43955590793342,5.21706327965227,-6.10642258804416,2.59570187732641e-09,6.93327482849688e-09,10.4635141296263

"ACSM5",-1.43964285805753,4.8107202136147,-7.04835693767915,9.02069457151368e-12,3.31745404388628e-11,15.9875840058321

"ITIH1",-1.44345051516454,9.24857833197013,-8.67169382995441,1.39199215229906e-16,9.76043786410642e-16,26.8618698344359

"IGSF23",-1.44347290027966,4.01194590693697,-8.01607154722161,1.47253786076767e-14,7.96570297991142e-14,22.2790423731548

"TF",-1.44386492063324,10.4398050074792,-8.03534651790646,1.2884117230918e-14,7.03137271971346e-14,22.4102365497194

"SLC39A5",-1.44406491060167,5.42242811387142,-6.17990475164074,1.70615283882287e-09,4.67194729786867e-09,10.8717193291476

"PRAP1",-1.4447719464446,8.98090140206835,-6.87618092650463,2.65581059569127e-11,9.20851131081611e-11,14.9317549400063

"GYS2",-1.46101789207359,3.95289010935633,-6.74983200377226,5.79312918714493e-11,1.92432299784035e-10,14.1697641473715

"PIPOX",-1.46167058663827,7.13026067011901,-10.3217408094563,4.23903416466108e-22,6.0304336986693e-21,39.3840427993907

"CYP2A7",-1.47313922243099,2.89200634621061,-4.95148429194001,1.12521220802539e-06,2.15056383125381e-06,4.58887865961229

"AFM",-1.47371629469217,6.43657157886296,-6.89077692012911,2.42532999383759e-11,8.45294635072489e-11,15.0204840932884

"RBP4",-1.48826268508714,12.542189127881,-9.85386167198204,1.76780107297757e-20,2.05456534758077e-19,35.702262352773

"APOH",-1.49732253740449,12.3380908937313,-7.87909946978223,3.78089712406721e-14,1.92558943031237e-13,21.3531382375813

"DAO",-1.50074270794999,4.42272024782181,-9.00133074419209,1.22341311818716e-17,9.96717901078144e-17,29.2557736228059

"AZGP1",-1.50387565711286,8.82490021329588,-8.94344691177829,1.88274114464469e-17,1.48958049385124e-16,28.8312453073925

"CYP2D6",-1.52709585182335,6.91869257991678,-7.61789622119687,2.2137669479724e-13,1.013701083109e-12,19.6190603472857

"PLG",-1.5345100938371,8.21209159577045,-7.94566861534215,2.39419148527739e-14,1.25432234506653e-13,21.8017278502686

"PCK1",-1.55198015801932,6.53644406794932,-5.80801163779368,1.37078551976335e-08,3.3138515955717e-08,8.84701156875916

"HSD17B6",-1.56097506183343,7.98966108260076,-7.33365609127549,1.44475988348591e-12,5.92439647344071e-12,17.7805002303611

"UGT2B10",-1.57687244079816,6.80304900713973,-6.72986773144993,6.5464956798714e-11,2.16195097285039e-10,14.0503667188499

"HRG",-1.58139375623214,8.66780602893131,-5.3870194185454,1.28266160498399e-07,2.75868272215992e-07,6.68181748851858

"FTCD",-1.58504701450576,7.11828879804193,-8.15922226747634,5.43252888585905e-15,3.11648952564113e-14,23.2586223644816

"FETUB",-1.61265533770593,5.49928655031217,-6.54513890612567,2.00328839311457e-10,6.19019460654345e-10,12.9587382581262

"HJV",-1.61273901094106,7.01993878360229,-7.73468663456655,1.00959981890937e-13,4.84496572194749e-13,20.3892238743883

"SLC22A1",-1.61628566847774,5.65399118494109,-5.23896016549889,2.72804657063871e-07,5.6370738844413e-07,5.95327434694303

"ALDOB",-1.62601578713021,10.6875710036543,-6.48366022659161,2.89170169250398e-10,8.75977201895913e-10,12.6007536673209

"G6PC",-1.63621727246446,7.60061826583377,-7.7309293201163,1.03555922579208e-13,4.95828438368732e-13,20.3643152399388

"CYP4A22",-1.65333662514482,4.84673880336895,-9.04853194372575,8.59721122136154e-18,7.19432918665407e-17,29.6032428494721

"KNG1",-1.66530707902099,10.052521938851,-9.40637940597901,5.71572916632282e-19,5.51625655680014e-18,32.2743443982803

"APOA5",-1.66717685574828,7.22126191136202,-7.87916415121333,3.77922372859015e-14,1.92539996778551e-13,21.3535728097338

"UPB1",-1.6718041567376,5.39441034854001,-9.42204516839179,5.06887205971326e-19,4.93056950187098e-18,32.3927352629401

"ADH1A",-1.67745209883719,7.84292865807465,-7.68901283540311,1.37380892714903e-13,6.47102931460358e-13,20.0870257605318

"ACSM2B",-1.69871643339022,5.68910873590941,-10.3445395739478,3.52600782405974e-22,5.07463869231166e-21,39.56587131276

"CYP2C9",-1.70066546230074,7.06670243093824,-7.08738140028069,7.04333226733855e-12,2.62947516768291e-11,16.2296576700688

"DCXR",-1.7087404632085,8.91395323655403,-12.6718440402814,8.92998634335766e-31,4.16779646529895e-29,59.1342706297831

"MASP2",-1.70882253732536,6.02803222791703,-8.7565369584044,7.48490030931738e-17,5.41511491815896e-16,27.4724775903606

"F12",-1.73747728747983,8.1621763475055,-9.80298212264657,2.63694480065649e-20,2.99184036240129e-19,35.3077755667807

"CYP4A11",-1.74862170614797,6.68301171959178,-8.63508003788722,1.81720111862757e-16,1.25809501872227e-15,26.5995714501598

"CDO1",-1.76215883955094,6.99172132147726,-10.0638208315014,3.35337880803059e-21,4.21522850168331e-20,37.3425371466392

"ACSM2A",-1.76568690796412,5.16952640373917,-10.2312125604263,8.78883361130317e-22,1.19184961759148e-20,38.6642227639369

"AGXT",-1.77086656812068,8.61075576875558,-8.77646884943196,6.46613673380445e-17,4.7406587203487e-16,27.6164888182884

"GNMT",-1.8000888131404,5.94387126275673,-7.52105605454218,4.21820601341547e-13,1.85518305494892e-12,18.9869114478996

"APOA1",-1.80097890874855,12.5317423165844,-7.25856997399118,2.35144478936422e-12,9.39498397478898e-12,17.3034628095967

"TTR",-1.81719983460395,10.2084943185726,-8.17272260085384,4.94193051606378e-15,2.85943926418317e-14,23.3516253174597

"APOA2",-1.82025056416295,14.0310971890247,-7.93748760985486,2.53283374104597e-14,1.32320887001324e-13,21.7464548545476

"TTC36",-1.82289881398626,3.29113222548401,-7.66462739516436,1.61855012958624e-13,7.55408491079762e-13,19.9262092377276

"ASPDH",-1.83697442866185,5.05698802285417,-9.73833529350674,4.37548448256534e-20,4.84908561195163e-19,34.8082652261863

"LECT2",-1.84703315903076,5.19071931428118,-7.43782208941801,7.30761562471105e-13,3.11394507971198e-12,18.448313230054

"ADH1B",-1.87491253302809,7.90364856531467,-7.66761694628577,1.58637472498008e-13,7.41327039042335e-13,19.9459048218423

"NR1I3",-1.87883407884692,4.718108394579,-10.9456735168066,2.54481273418133e-24,4.77798279215897e-23,44.4365337538319

"GSTA1",-1.89058015395073,9.32005290896941,-7.11131396277532,6.04871055418339e-12,2.28176115882568e-11,16.3786154498272

"CYP7A1",-1.90399923923985,4.41515077262661,-6.92168681201535,2.00021134766911e-11,7.06617165443278e-11,15.2088631778996

"HPR",-1.9082675399653,7.87645174715372,-8.84545763366478,3.89058700846585e-17,2.92932492571258e-16,28.1165856458527

"APOC3",-1.93660452298947,12.54632246072,-9.04142222039281,9.0670761329406e-18,7.55334410962028e-17,29.5508312666241

"CCL16",-1.93793218369054,5.96876023671781,-8.6936763897516,1.18572085694626e-16,8.40169543990415e-16,27.0197019850859

"CYP3A4",-1.95568029059048,5.9516211088207,-4.79006063785761,2.42368668961357e-06,4.47280086975587e-06,3.85225301196549

"CPS1",-1.96809369485917,6.98508541851021,-6.76817061093084,5.17652821277983e-11,1.73351595536617e-10,14.2796815745928

"GLYAT",-1.97924686226169,4.39635127479119,-7.86564311906474,4.1454233216394e-14,2.09752182091843e-13,21.2627847055529

"SULT2A1",-1.98873783694099,8.28925603844357,-8.38316158227998,1.11525023851685e-15,7.01237878404455e-15,24.8149269028019

"CYP2A6",-2.03827036946406,6.68674878932828,-5.32724476282205,1.74302250572858e-07,3.69423473122364e-07,6.38559715706395

"SLC27A5",-2.09009065419632,6.08430521074399,-11.6247122349754,8.24523879306665e-27,2.15908509634374e-25,50.1024412618418

"HPD",-2.11390148207329,8.62022428817109,-6.7125814907836,7.27592353998364e-11,2.3879167873571e-10,13.947208043612

"ADH4",-2.12100937663207,7.35906676814933,-6.60307599135544,1.41411959379076e-10,4.47240260584317e-10,13.2985406289064

"AHSG",-2.13826412209837,10.674536948043,-8.75538259529677,7.54854489880545e-17,5.45049886665821e-16,27.4641436653616

"TAT",-2.15586452899974,6.78700052491807,-7.21302103782641,3.1542861108692e-12,1.23951296176122e-11,17.0158698240474

"RTP3",-2.16592703979529,4.71861057946672,-10.122940126287,2.09260357406253e-21,2.70629981453279e-20,37.8079349264967

"SPP2",-2.21870892780011,5.88894140199157,-8.59364801241118,2.45486009388212e-16,1.6760339219652e-15,26.303640298746

"AQP9",-2.22044429093701,6.98559404736763,-8.69442476556934,1.17925893131625e-16,8.3599117818993e-16,27.025079853897

"SLC10A1",-2.23990285171138,5.79532492092301,-8.11410431023864,7.44813230323801e-15,4.18356558186812e-14,22.9485798909183

"BHMT",-2.25643833980908,6.30890188460779,-8.96555415348555,1.59726027202124e-17,1.27668642488137e-16,28.9931766440772

"SERPINC1",-2.34880293441762,11.0666223569118,-10.4178832195815,1.94689509916777e-22,2.89199929640434e-21,40.1522937938436

"CYP8B1",-2.4273807441705,5.66784002570375,-8.27421866122564,2.41782438192898e-15,1.46126273409474e-14,24.0542099590905
