# Supplementary material for: Identification and clinical validation of EMT-associated prognostic features based on hepatocellular carcinoma
Source: Cancer Cell Int. 2021 Nov 24;21:621. doi: 10.1186/s12935-021-02326-8 (PMC8613962; doi:10.1186/s12935-021-02326-8)
Supplement: Supplementary file 3 — Additional file 3: Table S3. 71 differentially expressed genes related to prognosis among molecular subtypes [file 12935_2021_2326_MOESM3_ESM.docx]

,p.value,HR,Low 95%CI,High 95%CI

CPB2,0.000208901043808292,0.764103886882618,0.662810649222691,0.880877141358303

CFHR4,0.000706855534114919,0.805756893929122,0.711091310839862,0.913025039424953

AMBP,0.00051919345231003,0.807872079726153,0.716170899870003,0.911315018970372

PROZ,0.00773436426853473,0.815492137377352,0.701836436474037,0.947553292424257

GPT,0.00163887151460416,0.764031116121458,0.646183753866682,0.903370818143838

BDH1,0.00574657766810672,0.787838523604292,0.665188319735398,0.933103484922722

C8A,0.000193800883166445,0.783933751798172,0.689734867883004,0.890997622165452

F13B,0.00635605754472892,0.83337654878478,0.731115392740137,0.949940979168092

HPX,0.000140928121456004,0.810697682703908,0.727663982647826,0.903206354050881

PON1,9.5416235160745e-05,0.833146190151746,0.760150005924017,0.913152099920859

HMGCS2,0.00728018116340958,0.850057346510067,0.754959538903552,0.957134064966163

ITIH1,0.0054553442772148,0.85209129719768,0.761126402279369,0.953927726834429

AFM,0.00895834704084961,0.860737861702391,0.769184827467723,0.96318809226524

RBP4,0.00189407121642562,0.822553076021222,0.727170771633401,0.930446587329386

FTCD,2.95798037355951e-05,0.815367705928441,0.740892001331891,0.897329833060504

G6PC,0.000982207642741425,0.83742226329871,0.753559565914035,0.930617934917617

CYP2C9,0.00178301592230886,0.860870957288999,0.783648379324297,0.945703232032064

MASP2,0.00321990506431744,0.843563599583819,0.753291416100164,0.944653730725892

CPS1,0.0010318467899687,0.87408265899417,0.806575927481991,0.947239396468818

SLC27A5,0.00671287491498812,0.84447335144366,0.747317832189896,0.95425963436301

SPP2,0.00281041612270413,0.870668830289146,0.79505382420419,0.953475335831324

MSC,0.00684399889216152,1.15371862044464,1.04014327238843,1.27969549051087

SLC1A5,7.36864324998825e-05,1.32938837135758,1.15481977656057,1.53034566758484

SLC38A1,0.0031381310320499,1.25030633139007,1.07806204138066,1.45007046191149

MMP14,0.00603132476339698,1.26951593178784,1.07070087466078,1.50524832771222

IMPDH1,0.000773169444217996,1.35747515810992,1.13594584424621,1.62220656400073

DAB2,0.00126762578634573,1.3384429693121,1.12103001216798,1.59802107227843

SMOX,0.00169519735285331,1.35269280914581,1.1201593916379,1.63349774110208

PNMA1,0.00279888191128968,1.36803714172756,1.11392291928549,1.68012129811151

ADAM9,0.00694299433963022,1.28402768425535,1.0708933152039,1.53958108667457

OLFML2B,0.00965553457592416,1.30945044604401,1.06760792354765,1.60607694344105

STK39,0.00341246032560445,1.27515058869202,1.08367218594833,1.50046208154607

ANXA5,0.00877384733186004,1.32230009031181,1.07297953791815,1.62955346961331

EGLN3,0.00306587525594167,1.24793213449336,1.07775688250762,1.44497765458727

HIF1A,0.00579321997064437,1.29241039570473,1.07713529554557,1.55071014554363

G6PD,3.21254413517117e-05,1.35172760113727,1.17270698290197,1.55807676965889

ATP1B3,0.00540634771204915,1.32578001943483,1.08687895395369,1.61719265382656

LHFPL2,4.31384246093401e-05,1.62697182345608,1.28850086227554,2.05435432122663

PFN2,6.40364268940631e-05,1.34958612709814,1.16509762473656,1.56328763855097

LAPTM4B,0.00173296159137516,1.29911706197668,1.10291270015978,1.53022550241232

PRNP,0.00782680456011263,1.33928881005602,1.07986729381086,1.66103235742173

LOX,0.00134442253946185,1.32606827921152,1.11594743582385,1.5757526068715

FZD7,5.67767748716045e-05,1.48016335658511,1.22291057046886,1.79153211615247

PAM,0.00871011820300592,1.32559378777435,1.07386715577063,1.63632799526768

RAB3IL1,0.00922496844635999,1.30157968487812,1.06732922597647,1.58724190704845

CSF1,0.00983369890026255,1.29849453451524,1.06491896475856,1.58330174592038

PLXNA1,0.00757561378928035,1.37100023789646,1.08757297876677,1.72829013685457

IGSF3,0.00122597545207505,1.32698180469581,1.117826283578,1.57527223671771

SLC25A24,0.000862338510949843,1.39839227969643,1.14806285972545,1.70330478975883

NT5DC2,0.00109793159255815,1.31757023456376,1.11649148753847,1.55486301721454

SLC2A1,0.000514724513709861,1.34121392106679,1.13643568018416,1.58289185514823

MYBL2,0.000644525791830572,1.27454374622047,1.10876168975869,1.46511353705163

SCPEP1,0.00424297658049065,1.31005212940774,1.08867102032081,1.57645105796974

MPZL1,0.00305586627580794,1.49777351431844,1.14645057827021,1.95675726691908

CEP55,2.50489749458958e-05,1.52244512251563,1.25212396186186,1.85112594413182

C5orf30,0.000522916234855372,1.40020227785617,1.15767738436997,1.69353435195644

RASSF3,0.0086873542857085,1.32338924838992,1.07349166213134,1.63146036856665

STX3,0.00151041021628344,1.48148054963828,1.16210719894335,1.88862492285752

ECT2,0.000123491362266457,1.48978098355827,1.21545399085597,1.8260233589005

B4GALT5,0.00233637959838476,1.4555265772629,1.1430019504628,1.85350306380567

TMEM45A,0.0046471922583355,1.19935984840703,1.05750349164014,1.36024519762099

POF1B,0.0094100511705786,1.21936150143485,1.0498389312437,1.41625770099807

EIF5A2,0.00229685497316771,1.36577142651116,1.11776637075252,1.66880274651539

SLC39A6,0.00984828686201409,1.38289163705017,1.0811372206907,1.76886822803265

RHOQ,0.00522390498923114,1.44434288748936,1.11588313733215,1.8694846322605

MTMR2,4.23899846392574e-05,1.83328387003194,1.37155287842898,2.45045583074348

OSBPL3,0.0058380088044176,1.44910731304669,1.11317329800749,1.88641966932204

EPB41L2,0.00678347863550613,1.36098464757787,1.08880262228756,1.70120752193914

GPD1L,0.00216024832078574,1.40193076110404,1.12971597490769,1.73973804264418

MMP1,4.76480557952818e-05,1.36449599027824,1.17469349664168,1.5849660467247

TOP2A,0.000242953910311306,1.35668617475789,1.15271134463281,1.59675480366292
